# Efficacy and Safety of Low-Dose Interleukin 2 for Primary Sjögren Syndrome: A Randomized Clinical Trial

**DOI:** 10.1001/jamanetworkopen.2022.41451

**Published:** 2022-11-10

**Authors:** Jing He, Jiali Chen, Miao Miao, Ruijun Zhang, Gong Cheng, Yifan Wang, Ruiling Feng, Bo Huang, Huijie Luan, Yuan Jia, Yuebo Jin, Xiaoying Zhang, Miao Shao, Yu Wang, Xia Zhang, Jing Li, Xiaozhen Zhao, Han Wang, Tian Liu, Xian Xiao, Xuewu Zhang, Yin Su, Rong Mu, Hua Ye, Ru Li, Xu Liu, Yanying Liu, Chun Li, Huixin Liu, Fanlei Hu, Jianping Guo, Wanli Liu, Wen-Bin Zhang, Alexander Jacob, Julian L. Ambrus, Changhai Ding, Di Yu, Xiaolin Sun, Zhanguo Li

**Affiliations:** 1 Department of Rheumatology and Immunology, Beijing Key Laboratory for Rheumatism Mechanism and Immune Diagnosis, Peking University People’s Hospital, Beijing, China; 2 Center for Applied Statistics and School of Statistics, Renmin University of China, Beijing, China; 3 Department of Clinical Epidemiology and Biostatistics, Peking University People’s Hospital, Beijing, China; 4 Institute for Immunology, School of Life Sciences, Tsinghua University, Beijing, China; 5 Beijing National Laboratory for Molecular Sciences, Key Laboratory of Polymer Chemistry & Physics of Ministry of Education, Center for Soft Matter Science and Engineering, College of Chemistry and Molecular Engineering, Peking University, Beijing, People’s Republic of China; 6 SUNY at Buffalo School of Medicine, Buffalo, New York; 7 Clinical Research Centre, Zhujiang Hospital, Southern Medical University, Guangzhou, China; 8 The University of Queensland Diamantina Institute, Faculty of Medicine, The University of Queensland, Brisbane, Queensland, Australia; 9 Ian Frazer Centre for Children’s Immunotherapy Research, Child Health Research Centre, Faculty of Medicine, The University of Queensland, Brisbane, Queensland, Australia; 10 State Key Laboratory of Natural and Biomimetic Drugs, School of Pharmaceutical Sciences, Peking University, Beijing, China

## Abstract

**Importance:**

Primary Sjögren syndrome (pSS) is a systemic autoimmune disease associated with dysregulated immune cells, with no efficient therapy. There is a need to study potential therapeutic approaches.

**Objective:**

To investigate the efficacy, safety, and immune response of low-dose interleukin 2 (LD-IL-2) in the treatment of pSS.

**Design, Setting, and Participants:**

A double-blind, placebo-controlled randomized clinical trial was conducted with a 2-group superiority design from June 2015 to August 2017. Sixty patients, aged 18 to 70 years, were recruited from Peking University People’s Hospital. Efficacy analyses were based on the intention-to-treat (ITT) principle. Data were analyzed from December 2018 to March 2020.

**Interventions:**

Patients with pSS were treated with LD-IL-2 or placebo for 12 weeks and accompanied by 12 weeks of follow-up.

**Main Outcomes and Measures:**

The primary end point was defined as a 3-point or greater improvement on the European League Against Rheumatism Sjögren’s Syndrome Disease Activity Index (ESSDAI) by week 24. The secondary end points included other clinical responses, safety, and changes of immune cell subsets at week 12 and 24.

**Results:**

Sixty patients with pSS were recruited, with 30 in the LD-IL-2 group (mean [SD] age, 47.6 [12.8] years; 30 [100%] women) and 30 in the placebo group (mean [SD] age, 51.0 [11.9] years; 30 [100%] women), and 57 completed the trial. More patients in the LD-IL-2 group (20 [66.7%]) achieved ESSDAI score reduction of at least 3 points than in the placebo group (8 [26.7%]) at week 24 (*P* = .004). There were greater resolutions of dryness, pain, and fatigue in the LD-IL-2 group than placebo group at week 12 (dryness: difference, −18.33 points; 95% CI, −28.46 to −8.21 points; *P* = .001; pain: difference, −10.33 points; 95% CI, −19.38 to −1.29 points; *P* = .03; fatigue: difference, −11.67 points; 95% CI, −20.65 to −2.68 points; *P* = .01). No severe adverse events were observed in either group. In addition, the LD-IL-2 group showed a significant decrease in infection compared with the placebo group (1 [3.3%] vs 9 [30.0%]; *P* = .006). Immunological analysis revealed that LD-IL-2 promoted an expansion of regulatory T cells and regulatory CD24^high^CD27^+^ B cells.

**Conclusions and Relevance:**

In this randomized clinical trial, LD-IL-2 was effective and well tolerated in patients with pSS, and it restored immune balance, with enhanced regulatory T cells and CD24^high^CD27^+^ B cells.

**Trial Registration:**

ClinicalTrials.gov Identifier: NCT02464319

## Key Points

**Question:**

Is low-dose interleukin 2 (LD-IL-2) effective in the treatment of primary Sjögren syndrome (pSS)?

**Findings:**

In this randomized clinical trial of 60 patients with pSS, patients receiving LD-IL-2 had significant improvement in their European League Against Rheumatism Sjögren’s Syndrome Disease Activity Index scores at week 24, and the immunological analysis found that LD-IL-2 induced expansion of regulatory lymphocytes, leading to the restoration of immune homeostasis in pSS. Additionally, there were no severe adverse events in the LD-IL-2 group.

**Meaning:**

These results suggest that LD-IL-2 could be an effective and safe treatment for patients with pSS.

## Teaser

This randomized clinical trial investigates the efficacy, safety, and immune response of low-dose interleukin 2 in the treatment of primary Sjögren syndrome.

## Introduction

Primary Sjögren syndrome (pSS) is a prevalent autoimmune disease characterized by xerostomia, xerophthalmia, and systemic involvement.^1^ Currently available treatments for pSS are symptomatic and empirical. Patients with systemic activity are generally treated with corticosteroids and immunosuppressive agents, which are associated with substantial adverse effects.^2,3^ Therefore, there is an unmet need for new therapies with better efficacy and lower adverse effects in patients with pSS.

Interleukin 2 (IL-2) is the key cytokine that regulates the homeostasis and activation of CD4^+^ T cells.^4^ It is required for the development, proliferation, and survival of regulatory T cells (Tregs). Although high-dose IL-2 in general enhances the activation of CD4^+^ T cells, downstream signaling induced by low-dose IL-2 (LD-IL-2) has been shown to selectively increase Tregs and suppress the differentiation of T follicular helper (Tf_h_)and T helper 17 (T_h _17) subsets.^4,5^

Recent studies have demonstrated that the dysregulation of T and B cells is functionally involved in the development of pSS. The production of multiple autoantibodies is indicative of the loss of B cell tolerance. As a hallmark feature of pSS, B cell hyperactivity results in hypergammaglobulinemia, autoantibody production, and increased risk of B cell lymphomas, especially B cell–derived non-Hodgkin lymphomas.^6,7^ These pathogenic B cell responses are initiated and reinforced by autoreactive effector T cells.^8,9,10^ Impairment of Treg cell function has been reported in patients with pSS, and it can be improved by LD-IL-2.^11^ It is possible that targeted therapies to pathogenic B and T cells may have potential significance in pSS. To evaluate the safety and potential efficacy of LD-IL-2 in pSS, we conducted a randomized, double-blind, placebo-controlled study in which we examined patient responses and clinical associations. We hypothesized that LD-IL-2 would improve clinical responses among patients with pSS, together with alterations in the profile of immunoregulatory cell subsets.

## Methods

### Study Design

**Figure 1.**
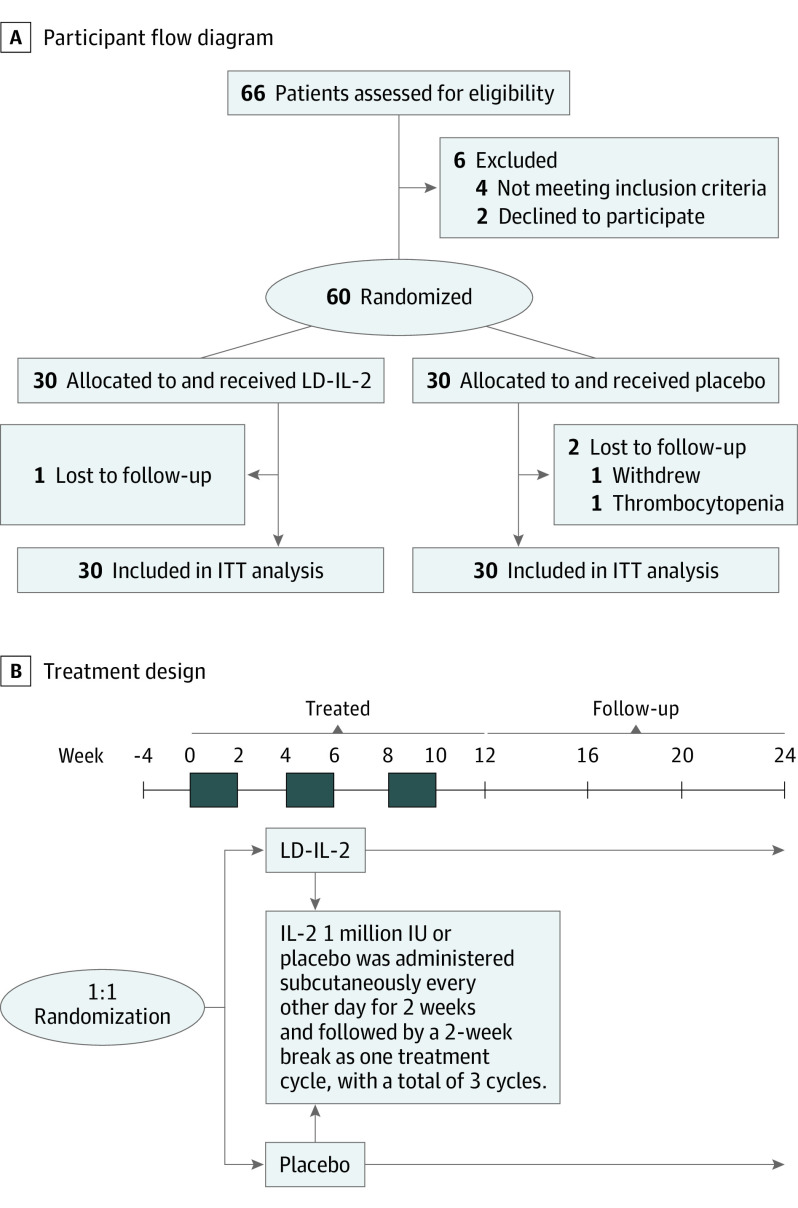
Study Flowchart and Treatment Design Flowchart shows recruitment, randomization, and study population. ITT indicates intention to treat; LD-IL-2, low-dose interleukin 2.

This study is a phase II, randomized, double-blind, placebo-controlled trial, with a 2-group, parallel-controlled, superiority design. Trial reporting was guided by the Consolidated Standards of Reporting Trials (CONSORT) reporting guideline. The study design is shown in Figure 1A. Ethics approval was obtained from the Peking University People’s Hospital Ethics Committee and was performed following the provisions of the Declaration of Helsinki^12^ and the International Council for Harmonisation guidelines for Good Clinical Practice. All participants provided written informed consent before study start. Full details of the trial can be found in the protocol (Supplement 1).

### Participants

Eligible participants were aged 18 to 70 years and fulfilled the 2002 American-European Consensus Group classification criteria for pSS.^13^ All patients had active disease at the time of enrollment, defined as a score of 5 or more points on the European League Against Rheumatism Sjögren’s Syndrome Disease Activity Index (ESSDAI),^14^ with scores of at least 50 mm on at least 2 of 3 visual analogue scales (VAS; a 100-mm VAS, with higher scores indicating more dryness, pain, or fatigue).^15^ Additional requirements include at least 1 of the 3 following characteristics: (1) hypergammaglobulinemia (defined as immunoglobulin G [IgG] level of ≥1680 mg/dL [to convert to grams per liter, multiply by 0.01]), (2) current parotid enlargement, and (3) systemic involvement. Interstitial lung disease (ILD) was diagnosed among patients with symptoms associated with ILD (such as cough or dyspnea) using high-resolution computed tomography (HRCT) showing interstitial pneumonia or an abnormal lung function test according to diffusing capacity for carbon monoxide of less than 70% or forced vital capacity of less than 80%. Patients had at least 4 weeks of stable background treatment with corticosteroids (≤7.5 mg/d prednisone or equivalent) and/or with antimalarials, nonsteroidal anti-inflammatory drugs, or immunosuppressants. Patients were excluded if they had secondary Sjögren syndrome or had a history of biologics usage, severe comorbidities, allergies to relevant reagents, active or chronic infection, or malignant neoplasm (eTable 1 in Supplement 2).

### Randomization and Blinding

Randomization was based on computer-generated random numbers prepared by a statistician who had no involvement in trial conduct. Eligible participants were randomly assigned in a 1:1 ratio to receive either recombinant human IL-2 or placebo in a blinded manner. The rheumatologists performing assessment of the safety and efficacy and the study participants were masked to the allocation sequence and the intervention (study drug containing IL-2 or matching placebo). The study drug was packaged, labeled, and randomly assigned by an independent third party (Beijing Stemexel Technology Co). The packaging and appearance of the placebo were identical to those of the active drug. At the study site, the study drug was matched to the independent randomization schedule and then distributed to each randomized study participant.

### Interventions and Procedures

After a 4-week screening period, patients were randomly assigned in a 1:1 ratio to receive IL-2 (recombinant human IL-2Ala^125^ [Beijing SL Pharma]) at a dose of 1 million IU or placebo subcutaneously every other day for 2 weeks, followed by a 2-week break as one treatment cycle. After the initiation of the therapy, patients could continue with concurrent medication but were prohibited from changing or adding disease-modifying therapy during the course of the study. Over 12 weeks (ie, 3 treatment cycles) and a 12-week observational follow-up, patients were assessed for clinical symptoms, and both routine laboratory tests and assays of immunological parameters were assessed at each visit (Figure 1B).

### Outcome Measures

The primary end point was defined as an improvement of ESSDAI to 3 or more points by week 24.^16^ The ESSDAI includes 12 domains, as follows: cutaneous, respiratory, kidney, articular, muscular, peripheral nervous system, central nervous system, hematological, glandular, constitutional, lymphadenopathic, and immunological, with a total score ranging from 0 to 123 points. Patients’ ESSDAI scores were assessed at weeks 4, 12, and 24.

Secondary end points were evaluated at week 12 and 24 and included values for the changes from baseline in clinical scores including European Alliance of Associations for Rheumatology SS Patient Reported Index (ESSPRI; including dryness VAS, pain VAS, and fatigue VAS), 20-Item Multidimensional Fatigue Inventory (MFI-20), and 36-Item Short Form Survey (SF-36); changes from baseline in clinical symptoms; special auxiliary examination, including ocular measures and salivary gland ultrasonography; immunological indexes, including IgA, IgG, and IgM, complement 3 (C3) and complement 4 (C4), and erythrocyte sedimentation rate (ESR). Safety outcomes included the frequency of injection site reaction, incidence of infection, and other adverse events (eTable 2 in Supplement 2). Changes in immune cells, including T and B cell subsets, were evaluated. Lung function was performed initially in all patients, but only those with impaired lung function underwent a repeated examination at week 24. Pulmonary CT was only performed at baseline. ILD improvement was based on the clinical symptoms and parameters, including persistent cough, breathlessness or dyspnea, and PFTs, including forced vital capacity and diffusing capacity for carbon. The diagnosis of type I renal tubular acidosis (RTA) was mainly based on the symptoms of hypokalemic weakness and persistent urine pH of greater than 5.3 even in the presence of metabolic acidosis induced by NH4+Cl loading. Kidney involvement was based on the improvement of symptoms, including weakness, hypokalemia, and urine pH.

For articular signs, pain and articular swelling were resolved according to the patients’ symptom and objective physical examination of rheumatologist. Parotid gland enlargement was confirmed by both symptoms and ultrasonography.

The treatment response was also measured by the Sjögren’s Tool for Assessing Response (STAR).^17^ STAR included 5 domains, as follows: (1) a 3-point or greater reduction in all clinical domains of the ESSDAI (3 points), (2) a 1-point or 15% or greater reduction in ESSPRI (3 points), (3) Schirmer test increase of at least 5 mm or ocular staining score decrease of at least 2 points (1 point), (4) unstimulated whole salivary flow increase of at least 25% or ultrasonography score decrease of at least 25% (Hocevar score) (1 point), and (5) IgG decrease of at least 10% or rheumatoid factor (RF) level decrease of at least 25% (1 point). A STAR response was defined as 5 or more points after treatment.

The study was conducted in compliance with Good Clinical Practice guidelines by the study investigators. Patients were assessed for clinical symptoms and given routine laboratory tests and assays for immunological parameters. Adverse events were coded using the Medical Dictionary for Regulatory Activities version 18.0.

### Immunological Analysis

Immunological analyses included enumeration of Tregs and CD24^high^CD27^+^ B cells. Additionally, protocol-specific immunophenotypic analyses of peripheral blood leukocyte subsets were performed at baseline and every 2 weeks thereafter until week 12 and every 4 weeks until week 24. In these immunophenotypic analyses, peripheral blood mononuclear cells (PBMCs) were incubated with the fluorophore-conjugated monoclonal antibodies (eTable 3 in Supplement 2). Relative proportions of Treg and CD24^high^CD27^+^ B subsets were analyzed by flow cytometry using a FACSAria II instrument (BD Biosciences) and FlowJo software (Tree Star). Tregs were defined as CD3^+^CD4^+^CD25^high^CD127^low^, and the CD24^high^CD27^+^ B cells were defined as CD3^-^CD4^-^CD19^+^CD24^high^CD27^+^ (eFigure 1 in Supplement 2).^18^

Measurements of multiple cytokines in serum samples were performed by flow cytometry using AimPlex human immunoassays kits (Quantobio), following the product instructions. Briefly, all samples were diluted 1:3 in the assay buffer. After coating antibody-conjugated beads onto each well, 45 μL of human standard and diluted sera were loaded, followed by the addition of secondary biotin antihuman antibody and Streptavidin-PE. Fluorescence intensity was detected using a BD Calibur flow cytometer, and serum cytokine concentrations were calculated using FCAP Array version 3.0. B cell activating factor (BAFF) was detected using an enzyme-linked immunosorbent assay kit (R&D, Inc).

Twenty healthy adult volunteers were recruited for serum BAFF detection. Five patients with pSS were recruited to examine IL-2 receptors in B cells, and 6 patients with pSS were recruited in an in vitro experiment. All autoimmune patients were not treated with immunomodulatory drugs or high-dose steroids (prednisone <10 mg/d) for 3 months preceding the study. PBMC were isolated from 6 patients with pSS, treated with IL-2 (50 IU/mL), CPG (0.1 μg/mL [Invivogen]) and anti-CD40 (3μg/mL [Biolegend]) for 72 hours and then were stained and analyzed using FlowJo software.

### Statistical Analysis

A sample size of 30 participants per group was estimated to provide at least 80% power to demonstrate the superiority of LD-IL-2 compared with placebo, with a 2-sided significance level of *P* < .05. We used an online software to conduct power analyses.^19^ Prior to unmasking, a detailed statistical plan was submitted to the institutional review board and a sealed envelope of the randomization list was provided to the statistician at the end of the study.

For clinical characteristics and laboratory parameters, the primary efficacy analysis was an intention-to-treat (ITT) analysis that included all patients who were randomly assigned to this trial and underwent at least 1 efficacy assessment. Binary efficacy was analyzed with a logistic regression model, adjusting for age, disease duration, and baseline ESSDAI score. Continuous variables were assessed with an analysis of covariance model, including treatment group and baseline ESSDAI score. For continuous variables, treatment differences across time points were evaluated using a mixed model for repeated-measures analysis, with a visit, treatment group, and treatment-by-visit interactions included in the model. The generalized estimation equations method in a logistic repeated-measures model was used for categorical variables, controlling for confounder variables including age, disease duration, and baseline ESSDAI score. Safety was assessed for patients who were randomly assigned and received at least 1 dose of study drug and analyzed by Fisher exact test. Statistical analysis was performed with the use of SPSS statistical software version 20.0. A 2-sided *P* < .05 was considered statistically significant.

## Results

### Patient Characteristics

A total of 66 patients were screened for eligibility, and 60 of them were randomly assigned to receive either LD-IL-2 (30 patients; mean [SD] age, 47.6 [12.8] years; 30 [100%] women) or placebo (30 patients; mean [SD] age, 51.0 [11.9] years; 30 [100%] women) (Figure 1A). Baseline characteristics did not differ between the groups (Table 1). Of the 60 patients who underwent randomization, 57 completed the trial (29 in the LD-IL-2 group and 28 in the placebo group) (Figure 1A). Three patients withdrew from the trial: 1 patient was unable to comply with visit schedules in the LD-IL-2 group; in the placebo group, 1 patient left the trial due to thrombocytopenia and another for personal reasons.

**Table 1.** Baseline Characteristics of Patients with Primary Sjögren Syndrome

Characteristics	Participants, No. (%)^a^	
	Low-dose IL-2 (n = 30)	Placebo (n = 30)
Age, y		
Mean (SD)	47.6 (12.8)	51.0 (11.9)
Median (IQR)	56 (45-61)	45 (33-59)
Female	30 (100)	30 (100)
Weight, median (IQR), kg	59.0 (52.8-64.3)	58.0 (52.5-67.5)
Height, median (IQR), cm	160.0 (157.0-164.3)	160.0 (157.0-165.0)
BSA, median (IQR), m^2^	1.59 (1.48-1.68)	1.56 (1.49-1.70)
Disease duration, median (IQR), y	4.5 (3.0-7.0)	3.0 (1.8-8.3)
Disease activity indexes, median (IQR)		
ESSDAI score	8.0 (6.0-12.0)	7.0 (6.0-11.0)
VAS score (range, 0-10)		
Dryness	7.0 (7.0-8.0)	7.5 (6.8-8.0)
Pain	4.0 (4.0-7.0)	5.0 (3.0-7.0)
Fatigue	7.0 (5.0-7.3)	6.0 (5.0-7.0)
ESSPRI score	6.3 (5.3-7.1)	5.8 (5.3-7.0)
MFI-20 score	51.5 (41.0-62.0)	51.0 (45.0-68.0)
SF-36 score		
PCS	50.3 (49.4-50.8)	50.4 (49.6-50.8)
MCS	50.1 (49.3-50.5)	49.6 (48.6-50.2)
Systemic signs		
Parotid gland enlargement	12 (40.0)	13 (43.3)
Articular	10 (33.3)	8 (26.7)
Leukopenia	12 (40.0)	7 (23.3)
Anemia	3 (10.0)	7 (23.3)
Thrombocytopenia	4 (13.3)	7 (23.3)
Pulmonary	12 (40.0)	11 (36.7)
Kidney	2 (6.7)	3 (10.0)
Neurologic	1 (3.3)	1 (3.3)
Cutaneous	1 (3.3)	0 (0.0)
Disease parameters, median (IQR)		
IgA, mg/dL	400 (300-510)	340 (260-510)
IgG, g/L	2290 (2070-2760)	2270 (2070-2550)
IgM, g/L	120 (90-150)	110 (80-170)
Hypergammaglobulinemia, No. (%)	28 (93.3)	29 (96.7)
ESR, mm/h	29.0 (19.0-40.0)	27.0 (14.0-44.0)
C3, g/L	100 (89-115)	97 (77-110)
C4, g/L	20 (14-25)	17 (15-21)
Anti-Ro/SSA positive, No. (%)	27 (90.0)	29 (96.7)
Anti-La/SSB, positive, No. (%)	14 (46.7)	14 (46.7)
ANA, ≥1:320 positive, No. (%)	14 (46.7)	20 (66.7)
RF, positive, No. (%)	24 (80.0)	27 (90.0)
DLCO, %	66.8 (61.2-70.1)	69.6 (59.4-73.6)
FVC, %	87.3 (84.7-94.2)	96.2 (90.0-99.3)
Background medication		
Glucocorticosteroids	1 (3.3)	3 (10.0)
Prednisone dose, median (range), mg/d	7.5(6.5-10)	5.0 (5.0-7.5)
Hydroxychloroquine	30 (100)	28 (93.3)
Cyclosporine	0 (0.0)	1 (3.6)
Tacrolimus	1 (3.3)	0 (0.0)
Azathioprine	0 (0.0)	1 (3.3)
Leflunomide	0 (0.0)	1 (3.3)

Abbreviations: ANA, antinuclear antibody; BSA, body surface area; C3, complement 3; C4, complement 4; ESR, erythrocyte sedimentation rate; ESSDAI, European League Against Rheumatism Sjögren’s Syndrome Disease Activity Index; ESSPRI, European League Against Rheumatism Sjögren’s Syndrome Patient Reported Index; IgA, immunoglobulin A; IgG, immunoglobulin G; IgM, immunoglobulin M; IL-2, interleukin 2; MCS, mental component scores; MFI-20, 20-Item Multidimensional Fatigue Inventory; PCS, physical component scores; RF, rheumatoid factor; SF-36, 36-Item Short Form Survey; VAS, visual analog scale.

^a^ This table included patients originally randomly assigned to IL-2 and placebo group.

### Outcomes

**Figure 2.**
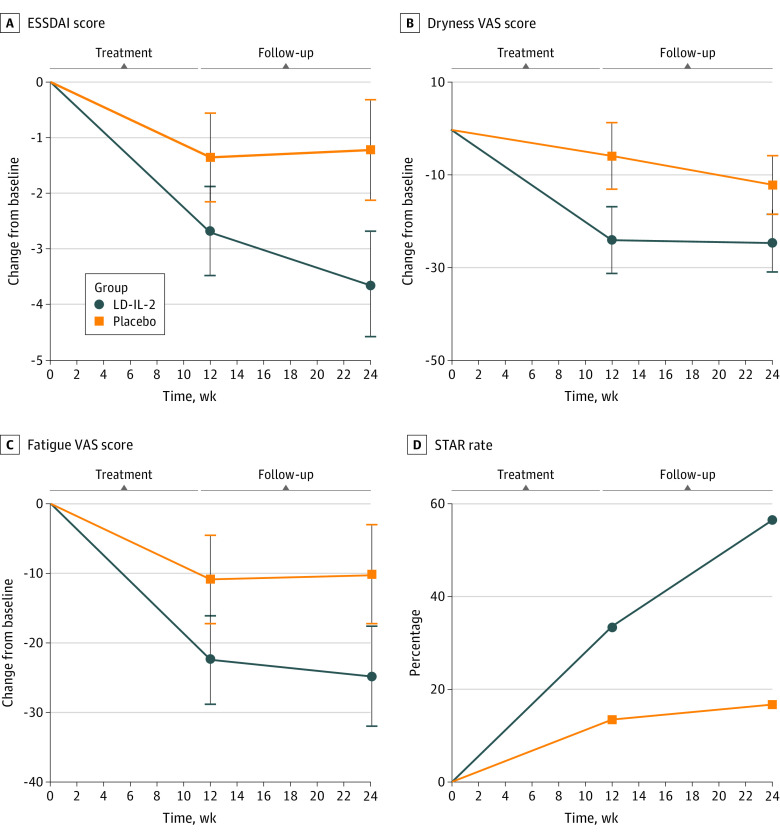
Clinical Responses to Low-Dose Interleukin 2 (LD-IL-2) Therapy Dots indicate least square means and whiskers indicate 95% CIs after adjustment for baseline characteristics in both groups. For continuous variables, treatment differences across time points were evaluated using a mixed model and a generalized estimating equations regression analysis for repeated-measures analysis; visit, treatment group, and treatment-by-visit interactions were included in the model. ESSDAI indicates European League Against Rheumatism Sjögren’s Syndrome Disease Activity Index; STAR, Sjögren’s Tool for Assessing Response; and VAS, visual analogue scale.

The rate of 3-point or greater improvement on ESSDAI scores in the LD-IL-2 and placebo groups at week 24 were 66.7% (20 of 30) and 26.7% (8 of 30), respectively (*P* = .004) (Table 2). At week 12 and 24, significant improvements in ESSDAI scores were also observed in the LD-IL-2 group compared with the placebo group (week 12, LD-IL-2 group: −2.68 points; 95% CI, −3.47 to −1.86 points; placebo group: −1.33 points; 95% CI, −2.14 to 0.53 points; *P* = .02; week 24, LD-IL-2 group: −3.67 points; −4.58 to −2.76 points; placebo group: −1.20 points; −2.11 to −0.29 points; *P* < .001) (Figure 2A). Patients treated with LD-IL-2 had greater improvements in changes of the ESSDAI scores than the placebo group from baseline over time (difference, −2.47 points; 95% CI, −3.75 to −1.18 points; *P* < .001) (Figure 2A and Table 2; eTable 4 in Supplement 2). At week 12, the STAR rates were 33.3% (10 of 30) in LD-IL-2 group and 13.3% (4 of 30) in placebo group, respectively (*P* = .07). At week 24, the STAR rate of the LD-IL-2 group was 56.7% (17 of 30), compared with 16.7% (5 of 30) of the placebo group (*P* = .001) (Figure 2D).

**Table 2.** Responses of Clinical Features at Week 12 and Week 24

Outcomes	Week 12				Week 24				*P* value^a^
	LD-IL-2 group	Placebo group	*P* value	Difference (95% CI)	LD-IL-2	Placebo	*P* value	Difference (95% CI)	
Patients with ≥3 points improvement, No. (%)									
ESSDAI	11 (36.7)	8 (26.7)	.34	1.59 (0.53 to 4.78)	20 (66.7)	8 (26.7)	.004	5.50 (1.8 to 16.68)	.02
STAR	10 (33.3)	4 (13.3)	.07	3.25 (0.89 to 11.90)	17 (56.7)	5 (16.7)	.001	6.54 (1.97 to 21.74)	.008
Change from baseline in disease scores and other parameters, LSM (95% CI)									
ESSDAI	−2.68 (−3.47 to −1.86)	−1.33 (−2.14 to 0.53)	.02	−1.33 (−2.27 to −0.20)	−3.67 (−4.58 to 2.76)	−1.20 (−2.11 to −0.29)	.000	−2.47 (−3.75 to −1.18)	<.001
VAS									
Dryness	−2.40 (−3.12 to −1.68)	−0.57 (−1.28 to 0.15)	.001	−18.33 (−28.46 to −8.21)	−2.47 (−3.10 to 1.83)	−1.20 (−1.83 to −0.57)	.006	−1.27 (−2.16 to −0.37)	<.001
Fatigue	−2.23 (−2.87 to −1.60)	−1.07 (−1.70 to −0.43)	.01	−11.67 (−20.65 to −2.68)	−2.48 (−3.18 to 1.75)	−1.00 (−1.72 to 0.28)	.005	−1.47 (−2.48 to −0.45)	<.001
Pain	−1.87 (−2.51 to −1.23)	−0.83 (−1.47 to 0.19)	.03	−10.33 (−19.38 to −1.29)	−1.93 (−2.78 to −1.09)	−1.40 (−2.27 to −0.56)	.38	−0.53 (−1.73 to 0.66)	.05
MFI-20	−1.37 (−5.18 to 2.45)	−2.87 (−6.68 to 0.95)	.58	1.50 (−3.89 to 6.89)	−0.90 (−4.80 to 3.00)	−3.37 (−7.27 to 0.54)	.38	2.47 (−3.05 to 7.98)	.29
SF-36									
PCS	0.00 (−0.26 to 0.26)	−0.26 (−0.51 to 0.00)	.16	0.26 (−0.11 to 0.62)	0.00 (−0.30 to 0.30)	−0.26 (−0.55 to 0.04)	.23	0.26 (−0.16 to 0.68)	.08
MCS	0.00 (−0.28 to 0.28)	0.58 (0.30 − 0.85)	.004	−0.58 (−0.97 to −0.19)	0.00 (−0.32 to 0.32)	0.58 (0.26 to 0.90)	.01	−0.58 (−1.03 to −0.13)	<.001
Patients with ≥1 point or 15% decrease in ESSPRI score, No. (%)									
≥1 point	25 (83.3)	14 (46.7)	.003	5.71 (1.72 to 18.94)	25 (83.3)	19 (63.3)	.08	2.90 (0.86 to 9.75)	
≥15%	26 (86.7)	14 (46.7)	.001	7.43 (2.08 to 26.55)	25 (83.3)	16 (53.3)	.01	4.38 (1.32 to 14.50)	
Systemic signs, No. (%)									
Parotid gland enlargement	3 (10.0)	11 (36.7)	.02	5.43 (1.27 to 23.11)	3 (10.0)	10 (33.3)	.04	4.54 (1.04 to 19.85)	.07
Articular	1 (3.3)	5 (16.7)	.14	5.56 (0.58 to 53.27)	0 (0.0)	6 (20.0)	.02	1.25 (1.05 to 1.50)	.30
Leukopenia	10 (33.3)	6 (20.0)	.27	0.51 (0.15 to 1.68)	8 (26.7)	8 (26.7)	.79	0.85 (0.25 to 2.85)	.19
Thrombocytopenia	2 (6.7)	4 (13.3)	.37	2.35 (0.36 to 15.31)	2 (6.7)	3 (10.0)	.47	0.89 (0.64 to 1.23)	.40
ILD	4 (13.3)	6 (20.0)	.23	3.76 (0.44 to 32.18)	3 (10.0)	5 (16.7)	.37	2.49 (0.34 to 18.38)	.33
Kidney	2 (6.7)	3 (10.0)	.70	1.51 (0.18 to 12.42)	2 (6.7)	3 (10.0)	.70	1.51 (0.18 to 12.43)	.66

Abbreviations: ESSDAI, European League Against Rheumatism (EULAR) Sjögren’s syndrome disease activity index; ESSPRI, EULAR Sjögren’s syndrome Patient Reported Index; LD-IL-2, low-dose interleukin 2; ILD, interstitial lung disease; LSM, least squares mean; MCS, mental component scores; MFI-20, 20-Item Multidimensional Fatigue Inventory; PSC, physical component scores; SF-36; 36-Item Short Form Survey; VAS, visual analog scale.

^a^ For continuous variables, treatment differences across time points were evaluated using a mixed model for repeated measures analysis. For categorical variables, Generalized Estimation Equations (GEE) was used for analysis.

The ESSPRI score, including dryness, pain, and fatigue, recovered more obviously in the LD-IL-2 group than in the placebo group at the end of week 12 (dryness: difference, −18.33 points; 95% CI, −28.46 to −8.21 points; *P* = .001; pain: difference, −10.33 points; 95% CI, −19.38 to −1.29 points; *P* = .03; fatigue: difference, −11.67 points; 95% CI, −20.65 to −2.68 points; *P* = .01) (Table 2 and Figure 2B and C). Quality of life and mental conditions of patients in the LD-IL-2 group were also improved, which was quantified by the SF-36 mental component summary (MCS) scoring system by week 24 (difference, −0.58 points; 95% CI, −1.03 to −0.13 points; *P* < .001) (Table 2).

In this study, pulmonary, articular, glandular, and hematological domains mostly contributed to the decreased ESSDAI score. Impaired lung function is one of the major risk factors for mortality in patients with pSS.^20^ Among 12 patients in the LD-IL-2 group with ILD, there was a significant improvement in diffusing capacity for carbon monoxide at week 12 (median [IQR],72.4% [70.3%-79.8%]; *P* = .01) and week 24 (median [IQR], 76.4% [71.9%-80.2%]; *P* = .003) compared with baseline (median [IQR], 66.8% [61.2%-70.1%]). Meanwhile, there was a significant improvement in the percentage of forced vital capacity at week 24 (median [IQR], 102.3% [75.3%-120.3%]) compared with baseline (median [IQR], 87.3% [77.7%-95.8%]; *P* = .03), but no significant change was observed in the placebo group (eTable 5 in Supplement 2).

Additionally, higher proportions of patients achieved recovery of thrombocytopenia and leukopenia at week 12 in the LD-IL-2 group but not in the placebo group (eTable 6 in Supplement 2). Increased resolution of clinical activity was observed in multiple disease manifestations in patients receiving LD-IL-2 treatment, including thrombocytopenia (2 of 4 [50.0%]), leukopenia (4 of 12 [33.3%]) and arthritis (10 of 10 [100%]), and persistent parotid gland swelling (9 of 12 [66.7%]) (Table 2 and eTable 6 in Supplement 2). There were reductions of IgG, anti-SSA, anti-SSB, and RF titers in the LD-IL-2 group, but some changes did not reach statistical significance (eTable 5 in Supplement 2). LD-IL-2 treatment was also associated with slight but not statistically significant improvements in the individual components of ocular parameters (eTable 7 in Supplement 2) and salivary gland ultrasonography scan scores (eTable 8 in Supplement 2).

### Safety

Notably, LD-IL-2 was well tolerated in this cohort of patients. There were fewer infections in the LD-IL-2 group (1 patient [3.3%]) compared with the placebo group (9 patients [30.0%]; *P* = .006). One case of upper respiratory tract infection was reported in the LD-IL-2 group, whereas 5 patients ([16.7%]) had upper respiratory tract infections, 3 (10.0%) had urinary tract infections, and 1 (3.3%) had herpes zoster in the placebo group during the trial (eTable 2 in Supplement 2). No injection site reactions were reported for the placebo group, whereas the LD-IL-2 group had injection site reactions in 3 patients (10.0%).

### Immunological Response

**Figure 3.**
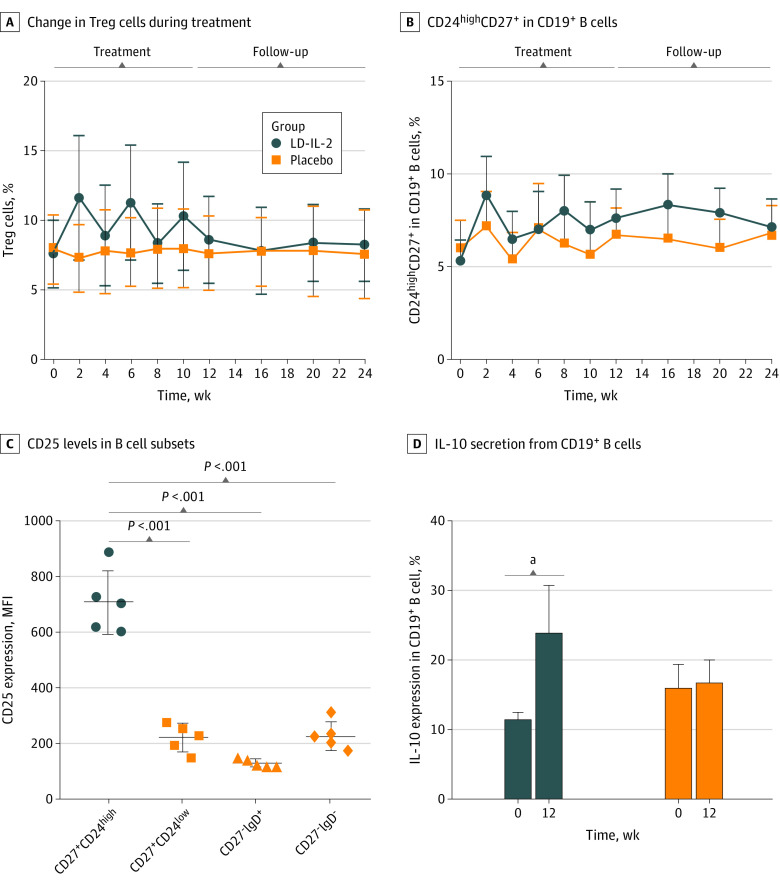
Immunological Responses to Low-Dose Interleukin 2 (LD-IL-2) Therapy

LD-IL-2 treatment induced the expansion of CD3^+^CD4^+^CD25^high^CD127^low^ Tregs (median [IQR] at baseline: 7.22% [5.18%-9.20%]; week 10: 9.65% [7.40%-12.70%]; week 24: 8.63% [6.46%-9.36%]) (Figure 3A and eFigures 1A and B in Supplement 2), while the ratio of Tregs to effector T cells (Tf_h_ and T_h_17) was rapidly increased following each cycle of LD-IL-2 administration (eTable 9 in Supplement 2). As expected, an increase in serum LD-IL-2 level resulted from each cycle of LD-IL-2 administration, accompanied by a significant reduction in the levels of pro-inflammatory cytokines, including IL-17A and interferon α (IFN-α) (eTable 10 in Supplement 2). These results established that LD-IL-2 altered the T cell immunoregulatory milieu of patients with pSS by potentiating immunoregulatory cells and downregulating pro-inflammatory cytokines.

In this study, a significant expansion of the CD19^+^CD27^+^CD24^high^ B cell population was observed during LD-IL-2 treatment periods, while these levels decreased in the interval period without LD-IL-2 administration (Figure 3B; eFigures 1C and D and eTable 11 in Supplement 2). This group oscillated between a median of 4.31% of CD24^high^CD27^+^ B cells at baseline (range, 1.28%-10.10%) to 6.03% (range, 2.80%-12.25%; *P* = .01) at week 12. No significant correlation between CD24^high^CD27^+^ B cells and Treg cells was observed (eFigure 3A in Supplement 2).

The median (IQR) BAFF level in the LD-IL-2 group at week 12 was 378.44 (208.72-595.38) pg/mL, while it was 351.58 (208.80-625.65) pg/mL in the placebo group (eTable 10 in Supplement 2). There was a slight increase in BAFF induced by LD-IL-2. However, there was a much higher median (IQR) BAFF level in patients with SS (363.8 [208.0-588.8] pg/mL) than in healthy control participants (181.5 [151.4-217.4] pg/mL) (eFigure 4 in Supplement 2).

To explore the expression of IL-2 receptors in different B cell subsets, we recruited 5 patients and found that the CD19^+^CD27^+^CD24^high^ B cells expressed the highest levels of CD25 among all B cell subsets (mean fluorescence intensity [MFI], 198). The CD19^+^IgD^-^CD27^-^ B cells and CD19^+^CD27^+^CD24^low^ B cells expressed intermediate levels of CD25 (MFIs, 87 and 96, respectively), while the expression of CD25 in naive B cells (CD19^+^IgD^+^CD27^-^) was barely detectable (MFI, 27) (Figure 3C and eFigure 2A in Supplement 2). Seeking to further understand the effects of LD-IL-2 on B cells generally and CD24^high^CD27^+^ B cells in particular, we next isolated PBMCs from 6 patients with pSS and costimulated with IL-2, CPG, and anti-CD40. The CD3^-^CD19^+^CD27^+^CD24^high^ B subset was dramatically expanded in both of the LD-IL-2 treated conditions. In accord with the observations from our clinical trial (Figure 3D), we found that LD-IL-2 treatment also induced increased IL-10 expression in B cells in vitro from 11.6% to 24.0% (eFigures 3B and C in Supplement 2). In contrast, the levels of tumor necrosis factorα and IL-6 remained unchanged on exposure to LD-IL-2 (eFigures 2B and C in Supplement 2). Collectively, these results strongly suggest that the anti-inflammatory effects we observed following IL-2 treatment can be attributed at least partially to a skewing of the B cell toward enrichment for the anti-inflammatory B cell subsets.

## Discussion

The management of pSS is challenging due to the heterogeneous nature of the disease and lack of safe, effective, and specifically targeted therapies. We have shown that LD-IL-2 confers benefits for the treatment of patients with pSS and resulted in a significant clinical improvement in ESSDAI, ESSPRI, and SF-36 MCS scores, as well as organ involvement including pulmonary lesions among patients with pSS.

In this study, we also assessed the efficacy of LD-IL-2 treatment with STAR, which was significantly higher in the LD-IL-2 group at week 24, supporting the efficacy of LD-IL-2 in pSS treatment. However, among the 5 STAR domains, we used the Schirmer I test and ultrasonography but not ocular staining score and nonstimulated salivary flow, which could influence the sensitivity of the response rate evaluation.

Our previous trials in patients with lupus showed that 1 million IU of IL-2 was efficient and safe.^5,21^ A dose of 1 million IU was chosen because it falls within the range used in recent clinical studies to treat hepatitis C virus–induced vasculitis (1.5-3.0 million IU), graft-vs-host disease (0.3-3.0 million IU per square meter of body surface area), and type 1 diabetes (0.33-3.00 million IU).^22,23,24^

We observed improvement of ESSDAI and some clinical parameters through week 24. LD-IL-2 treatment was discontinued at week 12, and expanded Tregs and Breg cells declined afterwards. There are a few explanations for the sustained clinical benefit after LD-IL-2 treatment. First, as shown in our previous report^5^ and another study,^25^ IL-2 treatment significantly improved the suppressive function of Treg cells,^5^ and such functional improvement of Tregs might be still present when IL-2 treatment was discontinued. In addition, IL-2 decreased inflammatory cytokines and autoantibodies, which can last in sera for a longer period of time after IL-2 discontinuation. Further studies are required to characterize the immunological changes with IL-2, which can help to improve the regimen of IL-2 therapy.

Besides the enhancement of CD4^+^ Tregs, IL-2 might induce the clinical improvement by other mechanisms. Many immune cells, including B cells, CD8^+^ T cells, natural killer (NK) cells, express IL-2Rs at various levels.^26^ Endothelial cells and smooth muscle cells were also reported to express IL-2Rs.^27,28^ The effects of LD-IL-2 on these IL-2Rs–expressing cells might also contribute to the clinical efficacy of LD-IL-2 in autoimmune disease treatment. Recent studies have found that LD-IL-2 shaped a tolerogenic gut microbiota that improves autoimmunity and gut inflammation, which are involved in the immunoregulatory effects of LD-IL-2.^29,30^ These emerging data suggest that other mechanisms could induce clinical improvement by LD-IL-2.

IL-2 is a fundamental immunoregulatory cytokine, inducing immune tolerance at low doses.^4^ Previous studies have reported that patients with pSS exhibit deficiencies in Treg cell function and have reduced IL-2 levels.^31,32,33^ In the present study, we found that LD-IL-2 treatment led to expanded Treg population, consistent with previous clinical trials of other autoimmune diseases.^5,22,23,24^ Decreased pro-inflammatory cytokines, including IL-17 and IFN-α, were also observed after IL-2 treatment. IFN-α is critical for pSS pathogenesis.^34^ IFN-α production from plasmacytoid dendritic cells (pDCs) can be induced by immune complexes composed of autoantibodies and autoantigens.^35^ We have previously demonstrated that IL-2 therapy inhibits Tfh differentiation and promotes Treg cells and T follicular regulatory subset, which resulted in reduced autoantibody production and decreased immune complex levels.^36,37^ This might alleviate the activation of pDCs or monocytes to lower IFN-α levels.

BAFF is another pathogenic cytokine in systemic lupus erythematosus. We observed a slight increase in BAFF levels in the group that received IL-2 compared with the placebo group at week 12 (eTable 10 in Supplement 2). It has been shown that IL-2 dose-dependently stimulated BAFF synthesis in PBMCs, likely through T and NK cells,^38^ and mediated by Erk1/2 and S6K1 signaling pathways.^39^ Notably, BAFF concentration in patients with SS was only slightly increased (4%) after LD-IL-2 treatment. The difference in BAFF levels between the LD-IL-2 and placebo groups at week 12 was smaller than the difference between patients with pSS and healthy controls. Therefore, the modest increase of BAFF concentration associated with LD-IL-2 treatment was unlikely to affect B cell homeostasis and differentiation or IgG production.

The identification of IL-10–producing regulatory B cells has expanded our understanding of the scope of immunoregulatory cells in the prevention of autoimmune diseases.^18^ The CD24^high^CD27^+^ B cell subset was shown to be enriched with IL-10 producing B cells and to be able to suppress the activation of effector T cells. In the present study, LD-IL-2 treatment effectively expanded the CD24^high^CD27^+^ B cell subset, which expressed higher levels of IL-2 receptors. Thus, it seems plausible that IL-2 may induce immune tolerance by targeting the CD24^high^CD27^+^ B cell subset.

We found that the LD-IL-2 group exhibited improvements for tear break-up time, meibomian gland area, and salivary gland ultrasonography scan scores. However, no statistical significance was achieved in these changes, which may be due to the irreversible damages in salivary and lacrimal glands of the long disease duration.^40^ Fatigue is a common clinical feature of pSS.^1^ This study showed that LD-IL-2 significantly alleviated this symptom, associated with reductions of IL-17 and IFN-α, which could contribute to the amelioration of fatigue (eFigure 5 in Supplement 2). These findings were consistent with previous reports characterizing the fatigue-promoting effects of these inflammatory cytokines.^41^ Certain pulmonary function parameters were also improved, which is notoriously difficult to treat clinically.

In general, LD-IL-2 was well tolerated in our trial. No serious adverse events were observed in this study. Different from many immunosuppressants or biologics, LD-IL-2 showed no increase in infection complications, which are the main cause of mortality in autoimmune diseases. This finding is pivotal for clinical practice. We found that fewer infections were recorded in patients receiving LD-IL-2 treatment than in the placebo group. It has been shown previously that LD-IL-2 can enhance the functions of CD8^+^ T cells and NK cells, both of which are required to mount immune responses against infections.^21,42,43^ The effects of LD-IL-2 therapy on CD8^+^ T cells and NK cells should be specifically addressed in future studies. In addition, LD-IL-2 treatment might also enhance other subsets of immune cells. Previous studies have evaluated the immune related adverse events (irAEs) related to high-dose IL-2.^44^ However, no irAEs have been reported from studies of LD-IL-2 therapy for autoimmune and inflammatory diseases.^21,22,23,24^

### Limitations

There are some limitations in this study. First, the cohort size is limited, leading to lack of clinical stratification and insufficient statistical power on the changes of some disease markers, such as autoantibodies. Therefore, optimizing the stratification and prolonging the course in a larger sample size would help deepen our understanding of the particular clinical features likely to benefit from LD-IL-2 therapy. Second, the improvement of immune function, including Treg and CD24^high^CD27^+^ B cells after the discontinuation of LD-IL-2 needs further characterization. Third, dosage and interval of LD-IL-2 administration have not been thoroughly compared.

## Conclusions

This study found that LD-IL-2 was effective and well-tolerated in patients with pSS. The clinical benefits might result from the effect of IL-2 to restore the balance of T and B cell subsets.

## Notes

Supplement 1.
Trial Protocol and Statistical Analysis Plan

Supplement 2. **eFigure 1.** Phenotypic Characterization of Treg and CD24^hi^CD27^+^B Cells After LD-IL-2 Therapy **eFigure 2.** Flow Cytometry Results Showing CD25 Expression on Different B Cell Subsets and the Changes of CD24^hi^CD27^+^ B Cells and Cytokines After Co-Stimulated With IL-2 In Vitro **eFigure 3.** Immunological Responses to LD-IL-2 Therapy **eFigure 4.** Serum BAFF Expression on SS and HC **eFigure 5.** Changes of Serum IFN-α in Patients With Fatigue **eTable 1.** Inclusion and Exclusion Criteria **eTable 2.** Adverse Events in Participants Who Received LD-IL-2 or Placebo Treatment **eTable 3.** Monoclonal Antibodies Used in Flow Cytometric Analysis in Human **eTable 4.** Decreased Scores From Baseline at Week 12 and Week 24 in Every Domain of ESSDAI **eTable 5.** Responses of Laboratory Parameters in Participants **eTable 6.** Percentage of Clinical Manifestations in Different Time Points of Both Groups **eTable 7.** Change in Ocular Parameters after LD-IL-2 Therapy **eTable 8.** Change in Salivary Gland Ultrasonography Scan Score After LD-IL-2 Therapy **eTable 9.** Immunological Responses of T Cell Subtypes to LD-IL-2 Therapy **eTable 10.** Change in Cytokines After LD-IL-2 Therapy **eTable 11.** Changes of B Cell Subsets After LD-IL-2 Therapy

Supplement 3.
Data Sharing Statement
