# Supplementary material for: Efficacy and Safety of Low-Dose Interleukin 2 for Primary Sjögren Syndrome: A Randomized Clinical Trial
Source: JAMA Netw Open. 2022 Nov 10;5(11):e2241451. doi: 10.1001/jamanetworkopen.2022.41451 (PMC9650609; doi:10.1001/jamanetworkopen.2022.41451)
Supplement: Supplement 1. — Trial Protocol and Statistical Analysis Plan [file jamanetwopen-e2241451-s001.pdf]

This supplement contains the following items:

1. Original protocol (NCT02464319) in English
2. Final protocol (NCT02464319) in English
3. Summary of changes (NCT02464319)
4. Original protocol (NCT02464319) in Chinese
5. Final protocol (NCT02464319) in -Chinese

Clinical Protocol

Low-dose Interleukin-2 treatment of active Sjögren's syndrome: a randomized, double-blind and placebo-controlled clinical trial

Research Institution： Peking University People's Hospital

2014/11/29

Contents

1. Title 4
2. Abstract 4
3. Background 4
4. Objectives 5
5. Study Design 5
5.1. Sjögren's Syndrome (SS) 5
5.2. Procedures for Blinding 5
5.3. Emergency Unblinding 5
5.4. Drug Allocation 5
5.5. Concomitant Therapy Requirement. 6
5.6. Study Population 6
5.7. Inclusion and Exclusion Criteria 6
5.8 Subject withdrawal or discontinuation 8
6. Study Drug Information 9
6.1. Drugs 9
6.2. Dose and administration 9
6.3. Concomitant therapy 9
7. Study Procedures 11
7.1. Screening Phase 11
7.2. Double-Blind Treatment Phase 11
7.3. Follow-up Phase 12
7.4. Subjects Withdrawing from Study Participation 12
7.5. Efficacy Evaluations 12
7.6. Follow-up 12
7.7. Biomarkers 14
8. Study drug information 15
8.1. Physical Description of Study Drug 15
8.2. Subcutaneous administration 15
8.3. Packaging The investigational supplies will be uniquely packaged to assure that they are appropriately managed throughout the supply chain process. 15
8.4. Labeling 15
8.5. Preparation, Handling, and Storage 15
8.6. Drug Accountability 15
9. Safety Evaluations 16
9.1 Adverse Event 16
10. Statistical analysis 18
10.1. Subject Information 18
10.2. Sample Size Determination 19
10.3. Efficacy Analyses 19
10.4 Safety analyses 20

11. Data Quality Assurance/Quality Control 21
12. Data management 21
13. Ethics 21
14. Summary and data preservation 21
ATTACHMENT 22
1. Time and Events Schedule for SS Design 22
2. VAS score 25
3. EULAR Sjögren’s syndrome disease activity index (ESSDAI): Domain and item definitions and weights. 26
4. MOS 36-Item Short Form Survey Instrument (SF-36) 31
5.Multidimensional fatigue inventory-20, MFI-20 35

1. Title

Low-dose Interleukin-2 treatment of active Sjögren’ssyndrome: a randomized, double-blind and placebo-controlled clinical trial.

2. Abstract

Low-dose recombinant human interleukin-2 (rhIL-2, trade name: Xinjier) has been reported to be effective in the treatment of various autoimmune or inflammation-related diseases in a series of pilot open-label clinical studies, but its real effects on autoimmune diseases has not been confirmed in clinical trials with strict control groups. This study aims to confirm and clarify the safety and efficacy of rhIL-2 for Sjögren’s syndrome(SS) in a randomized, double-blind and placebo-controlled clinical trial, and further evaluate the effect of the treatment on immune cell subsets to explore the immune mechanism of treatment.

3. Background

Primary Sjögren’s syndrome (pSS) is a chronic autoimmune disease characterized by xerostomia, xerophthalmia and multiple organ involvement, including peripheral neuropathy, lymphadenopathy, renal tubular acidosis and interstitial lung disease. Recent studies have demonstrated that dysregulation of T cells and B cells is critical in the development of pSS[1]. The production of multiple autoantibodies is indicative of the loss of B cell tolerance. Currently available treatment for SS is symptomatic andempirical. Sicca manifestations are treated through administration of topical therapies, such as saliva substitutes and artificial tears. Patients with systemic damage are generally treated with corticosteroids in combination with immunosuppressive agents[2].

Low-dose rhIL-2 has been used to treat autoimmune diseases such as HCV-induced vasculitis and type 1 diabetes (T1D)[3, 4]. This project aims to confirm the efficacy and safety of rhIL-2 in the treatment of SS.

4. Objectives

The study aims to clarify the safety and efficacy of low-dose rhIL-2 in the treatment of SS, and further explore the immune mechanism of low-dose IL-2 treatment.

5. Study Design

5.1. Sjögren's Syndrome (SS)

Adjust the drug treatment for active SS based on the original treatment as follows:
Experimental group（n=30）：original treatment plan + rhIL-2
Control group（n=30）：original treatment plan + placebo

5.2. Procedures for Blinding

We designed a double-blind study. There was no difference in the drug packaging or dosage between rhIL-2 and placebo. Independent third party handle the blind bottom generation and on-site blinding. Using SAS 9.3 statistical software to generate one random coding table in a ratio of 1:1, namely SS 01-60. The selected subjects were randomly assigned to the experimental group or the control group.

5.3. Emergency Unblinding

Each subject will have an emergency unblinded envelope in which the specific medication used by the subject is recorded and can only be opened when necessary. Emergency envelopes are kept by researchers responsible for this trial. The main investigator must be notified before opening the envelope, and the emergency envelope can be opened after approval. After unblinding, the subject will be discontinued from further administration of study agent and will be treated as a shedding case.

5.4. Drug Allocation

Subjects who meet the inclusion criteria, according to their indications, will be provided with the drugs by the drug administrators in the order of enrollment time. The drug will also be provided in the order of the drug number from small to large.

5.5. Concomitant Therapy Requirement.

The original treatment regimen included glucocorticosteroid with or without immunosuppressive agents, in which prednisone acetate was initiated in the dosage of ≤ 7.5 mg/d.

5.6. Study Population

Sixty cases of SS, either outpatient or inpatient.

5.7. Inclusion and Exclusion Criteria

5.7.1 Inclusion Criteria Applicable to All Subjects

Male or female 18-70 years of age at time of screening.

Diagnosis of primary Sjögren's syndrome (according to the 2002 AECG classification criteria)[5] for ≥3 months before screening.

European League Against Rheumatism Sjögren's Syndrome Disease Activity Index (ESSDAI) ≥5.

Positive tests for RF, anti-SSA or anti-SSB antibodies*.

Patients with a diagnosis of primary Sjögren's syndrome with more than 10 years disease duration must have at least one systemic feature of following items,

Hypergamaglobulinaemia* (IgG over 16.8g/L [Upper limit of normal level]) or Low complement C3 / C4*, or Cryoglobulinaemia*

OR

Active/past history since diagnosis of the following (ascribed to Sjögren's syndrome)

Purpura / cutaneous vasculitis,

Lymphadenopathy,

Persistent parotid salivary gland swelling not due to infection,

Peripheral neuropathy (previously documented by nerve conduction tests),

Interstitial lung disease confirmed by HRCT,

Renal tubular acidosis requiring treatment,

CNS disease ascribed to Sjögren's syndrome (confirmed by MRI),

Myositis (CK>2 times the ULN) and EMG or biopsy evidence of myositis,

Inflammatory arthritis.

An unstimulated salivary flow rate greater than 0 ml in 15 minutes.

Symptomatic oral dryness (≥ 50/100 on VAS).

Symptomatic fatigue (≥ 50/100 on VAS).

Symptomatic pain (≥ 50/100 on VAS).

Patients on corticosteroids (≤7.5 mg/d prednisone or equivalent), DMARDs (e.g. methotrexate, hydroxychloroquine, azathioprine, MMF, leflunomide, ciclosporin etc.), or pilocarpine*** must have been on a stable dose for 4 weeks prior to receiving the first infusion of study medication and expected to remain on this dose throughout the study. If they have stopped any of these drugs they should have been off it for at least 4 weeks prior to receiving study medication.

Given their written informed consent to participate in the trial and expected to be able to adhere to the study visit schedule and other protocol requirements.

*Anti-Ro antibody test, IgG, RF, C3/C4 and Cryoglobulinaemia assays performed within 3 months of screening may be used to confirm eligibility. If greater than 3 months repeats should be performed locally at screening to confirm eligibility.

VAS range 0-100mm with 100mm corresponding to worst severity.

***Pilocarpine or drugs with similar pharmacological action should not be used within 12 hours of the assessment visits at screening, baseline, week 12, week 24 (end of study).

5.7.2 Exclusion Criteria

Patients will be excluded from the trial for the following reasons,

Diagnosis of Secondary Sjögren's syndrome.

Any AECG exclusion criteria not covered elsewhere (graft versus host disease, primary lymphoma excluding PSS, sarcoidosis).

Stable disease activity (IgG <16.8 g/L) at the time of enrolment.

Prior rituximab or monoclonal antibody usage.

Severe comorbidities: including

Heart failure (≥ grade III NYHA)

Renal insufficiency (creatinine clearance ≤30 ml/min);

Hepatic insufficiency (serum ALT or AST >3 times the ULN, or total bilirubin >ULN for the central laboratory conducting the test).

Other severe, progressive or uncontrolled hematologic, gastrointestinal, endocrine, pulmonary, cardiac, neurologic, or cerebral disease (including demyelinating diseases such as multiple sclerosis).

Known allergies, hypersensitivity, or intolerance to IL-2 or its excipients.

History of severe allergic reaction to monoclonal antibodies or to murine, chimeric, or human proteins or their excipients.

Had a severe infection (including, but not limited to hepatitis, pneumonia, sepsis, or pyelonephritis); had been hospitalized for an infection; or had been treated with IV

antibiotics for an infection, within 2 months prior to the first administration of study agent.

Chest radiograph within 3 months prior to the first administration of study agent that showed an abnormality suggestive of a malignancy or current active infection, including TB.

Had a nontuberculous mycobacterial infection or opportunistic infection (e.g., cytomegalovirus, pneumocystosis, aspergillosis) within 6 months prior to screening.

Infected with HIV (positive serology for HIV antibody) or hepatitis C (positive serology for Hep C antibody). If seropositive, consultation with a physician with expertise in the treatment of HIV or hepatitis C virus infection was recommended.

Infected with hepatitis B virus. For patients who were not eligible for this study due to hepatitis B virus test results, consultation with a physician with expertise in the treatment of hepatitis B virus infection was recommended.

Had any known malignancy or has a history of malignancy within the previous 5 years (with the exception of a non-melanoma skin cancer that had been treated with no evidence of recurrence for ≥3 months before the first study agent administration or cervical neoplasia with surgical cure).

Had uncontrolled psychiatric or emotional disorder, including a history of drug and alcohol abuse within the past 3 years that might prevent the successful completion of the study.

Received, or was expected to receive, any live virus or bacterial vaccination within 3 months before the first administration of study agent, during the study, or within 4 months after the last administration of study agent. Had a BCG vaccination within 12 months of screening.

Pregnancy, lactation or women of child-bearing potential (WCBP) unwilling to use medically approved contraception whilst receiving treatment and for 12 months after treatment has finished.

Men whose partners are of child-bearing potential but who are unwilling to use appropriate medically approved contraception whilst receiving treatment and for 12 months after treatment has finished.

5.8 Subject withdrawal or discontinuation

Discontinuation of Study Treatment

A subject will be withdrawn from the study for any of the following reasons:

An AE temporally associated with study agent infusion or injection

Withdrawal of consent

Participation being deemed unlikely compliant with medical follow-up or study visit schedule

Malignancy

Pregnancy or planning to become pregnant within the study period

The initiation of prohibited medications or treatments

Lost to follow-up

Death

If a subject is lost to follow-up, every reasonable effort must be made by the study site personnel to contact the subject and determine the reason for withdrawal. The measures taken to follow-up must be documented. When a subject withdraws before completing the study, the reason for withdrawal is to be documented in the CRF and in the source document. Study drug assigned to the withdrawn subject may not be assigned to another subject. Subjects who withdraw from this study will not be replaced. A subject who withdraws from the study will have the following options regarding the optional research samples:

The collected samples will be retained and used in accordance with the subject's original informed consent for optional research samples.

The subject may withdraw consent for optional research samples, in which case the samples will be destroyed and no further testing will be needed.

6. Study Drug Information

6.1. Drugs

Recombinant human Interleukin-2, placebo

6.2. Dose and administration

The patients received low-dose IL-2 or placebo for 3 cycles. The method is 1 million units subcutaneously administrated every other day (rhIL-2 1×10^6 IU, SC, Qod) for a period of 14 days. After a 14-day break, another cycle started. All the patients will be treated for the first 12 weeks which included three treatment cycles with IL-2 or placebo and followed up for further 12 weeks without study medicine.

6.3. Concomitant therapy

All pre-study therapies administered up to 30 days before entry into screening must be recorded at screening. All concomitant therapies must be recorded throughout the study and any changes must be recorded throughout the study. Every

reasonable effort should be made to keep concomitant medications stable through Week 24 or early termination.

6.3.1 Immunomodulators

If receiving immunomodulators, subjects should be receiving stable dosing from screening through Week 24 or early termination. A reduction in immunomodulators is allowed only if the subject develops unacceptable side effects, with the implication that this may affect interpretation of the subjects' clinical data.

6.3.2 Antimalarial Medications

Stable treatment with hydroxychloroquine is permitted through the trial.

6.3.3 Corticosteroid Therapy

Unnecessary dose changes are discouraged, and any dose adjustments should be made in increments. Changes in corticosteroids are allowed for medical necessity, but the degree and timing of the adjustment should be carefully considered as this may have an impact on the study results.

The original treatment regimen included glucocorticosteroid with or without immunosuppressive agents, in which prednisone acetate was initiated in the dosage of <7.5mg/d.

If subjects experience a worsening in their disease activity while tapering corticosteroids, further dose decreases may be suspended, and/or their oral corticosteroid dose may be temporarily increased if deemed necessary by the investigator.

6.3.4 Nonsteroidal Anti-inflammatory Drugs

Subjects treated with NSAIDs, including aspirin and selective cyclooxygenase-2 (COX-2) inhibitors, and other analgesics should receive the usual marketed doses approved in the country in which the study is being conducted. Prescriptions of NSAIDs and other regularly administered analgesics should not be adjusted for at least 2 weeks prior to the first administration of the study drug and through Week 24, and may be changed only if the subject develops unacceptable side effects. The addition of new NSAIDs to the treatment regimen is not permitted during the period between 0 and 12 weeks. Minor adjustments in NSAID therapy are allowed after Week 12 although it is recommended that the use of any NSAIDS remain as stable as possible, and any notable changes should be recorded.

6.3.5 Topical Medications

Topical medications are permitted; however, topical compounds cannot include a prohibited medication. Topical ointments or creams of cyclosporine A are prohibited through Week 24; however ophthalmic use is permitted. Topical ophthalmic drugs can be used, such as sodium hyaluronate eye drops for dry eyes and deproteinized calf blood extract eye gel. Low potency topical steroids are allowed except on day of study visit. Medium to high potency topical corticosteroids are not allowed during the study. For 72 hours prior to study visit, topical medications should not be applied to lesions under evaluation.

7. Study Procedures

7.1. Screening Phase

Written informed consent must be obtained and reviewed by investigator before any screening data is collected. The screening visit must be performed no more than 4 weeks prior to the randomization visit (Week 0). In addition, to be eligible for study participation, subjects must have received approval for study randomization following review and adjudication of screening SS.

Repeat or unscheduled samples may be taken for safety reasons or for technical issues with the collection or analysis of specific samples.

All screening evaluations establishing subject eligibility will be performed and reviewed by investigator before subject can be randomized. All enrollment subjects must meet the classification criteria for these diseases.

7.2. Double-Blind Treatment Phase

7.2.1. Week 0/Day of Randomization

At Week 0, eligible subjects will be randomly assigned in a 1:1 ratio to receive either recombinant human Interleukin-2 or placebo in a blinded manner.

7.2.2. Placebo-controlled Treatment Period

The patients were treated with low-dose rhIL-2 or placebo in the same way.

Low-dose IL-2 treated with a 12-week treatment including 3 treatment cycles. IL-2 at a dose of 1 million IU or placebo was administered subcutaneously every other day for 2 weeks, followed by a 2-week break in each treatment cycle.

7.3. Follow-up Phase

The observation period was 12-week treatment and the 12-week follow-up. The clinical manifestations, autoantibodies and other related laboratory tests were collected, the patients with SS were assessed with ESSDAI score assess the degree of remission of their condition. Adverse drug reactions were monitored before and after different courses of treatment.

7.4. Subjects Withdrawing from Study Participation

Subjects who withdraw from study participation will not be required to return for any follow-up assessments.

7.5. Efficacy Evaluations

Efficacy evaluations will be done based on the primary and secondary outcome measures (see section 7.5.3). A complete list describing all efficacy evaluations and endpoints, and which evaluations are included in the composite endpoints is provided in Attachmenttable 1 & 2. All efficacy evaluations should be consistently performed by the study investigator to achieve comparable measures over time. These data will be reviewed at every visit that these data are collected and may require reconciliation of inconsistencies across assessments.

7.5.1. Endpoints

Primary Endpoint

The primary endpoint was defined as an improvement of ESSDAI to ≥ 3 points by week 24

Major Secondary Endpoints

The secondary endpoints includedother clinical responses, safety and changes of immune cell subsets including Treg, Tfh, Th17 cell and CD8+T cells.

7.6. Follow-up

7.6.1. Baseline follow-up

(1) General information: including name, sex, age, contact number, course of disease.

(2) Clinical symptoms and signs: including rash, fever, oral ulcer, foamy urine, disease activity score and so on.

(3) Laboratory examination: blood routine examination, liver and kidney function, ANA, anti-SSA, anti-SSB, RF, immunoglobulin, ESR, CRP, T cell and B cell subsets, IL-2, TGF-12β and other cytokines.

7.6.2. Follow up visits

Follow up once every 2 weeks in the first 3 months (6 times), and then follow up once a month:

(1) According to disease activity score, including ESSDAI score, to observe whether the clinical symptoms are relieved.

(2) Monitoring of changes in serological indicators： Every 4 weeks, blood routine examination, liver and kidney function, according to the disease related autoantibodies such as anti-SSA, anti-SSB, RF, immunoglobulin, ESR, CRP to evaluate the condition.

(3) Laboratory flow detection: At week 0, 6, 12 and 24, CD4+T and CD8+T cells subtypes were detected by flow cytometry, to evaluate the changes of cell group ratio before and after treatment.

(4) Cytokines: the levels of IL-2, IL-6,TGF-13β and other cytokines were detected by ELISA at 0, 6, 12 and 24 weeks.

7.6.3. Evaluations

A complete list describing all efficacy evaluations and endpoints, and which evaluations are included in the composite endpoints is provided in Attachment2.

7.6.3.1. ESSDAI

The European League Against Rheumatism (EULAR) organized an international collaboration of global SS experts in 2010 to develop two recognized disease activity indicators[7]. That is, the SS patient reporting index (EULAR Sjögren’s syndrome patient reported index) based on the patient's subjective symptoms and the SS disease activity index based on the patient's objective systemic symptoms. (EULAR Sjögren’s syndrome disease activity index, ESSDAI).

At screening (Attachment 6), ESSDAI is an index of disease activity that covers 12 aspects of the body, including systemic conditions, lymphadenopathy, glands, skin, joints, lungs, kidneys, muscles, peripheral nervous systems, the central nervous system, blood system and biochemistry. Each aspect is divided into 3 ~ 4 levels according to its activity degree, and the weight of each aspect is obtained by multivariate linear regression model. Score only the symptoms associated with the disease to avoid the effects of long-term clinical symptoms. The final score ranges from 0 to 123, and 0 indicates disease-free activity.

7.6.3.2. Fatigue, pain and dryness VAS score

Visual analogue scale VASs (scores range from 0 [none] to 100mm [worst] for dryness, pain, and fatigue).

7.6.3.3. MFI-20

The questionnaire comprises 20 items for which the person must specify the extent to which the particular statements relates to him/her on a five-point scale, ranging from “Yes”, that is true or “No”, that is not true. An equal number of items is worded in a positive and in a negative direction to counteract response tendencies.

Higher scores indicate a higher degree of fatigue.

7.6.3.4. Short-Form-36

The RAND short-form (SF)-36 questionnaire is a self-administered multi-domain scale with 36 items9. Eight health domains cover a range of functioning (Attachment 3):

-Limitations in physical function

-Limitations in usual role activities

-Bodily pain

-General mental health (psychological distress and well-being)

-Vitality (energy and fatigue)

-Limitations in social functioning due to physical or mental health problems

-Limitations in usual role activities due to personal or emotional problems General health perception

-The subscales are scored from 0 to 100

The scoring yields a Physical Component Summary score and a Mental Component Summary score, a total score, and subscale scores. Higher scores represent better outcomes. It is appropriate for persons over the age of 14 and may be completed in 5 to 10 minutes. Translations are available in most languages; the instrument has undergone extensive linguistic and cultural validation.

The concepts measured by the SF-36 are not specific to any age, disease10, or treatment group, allowing comparison of relative burden of different diseases and the benefit of different treatments. A change of 3 points in any of the subscales or 5 points for the component score is associated with clinically meaningful change.

7.7. Biomarkers

The collection, preparation, storage and shipment of blood, serum and urine are detailed in the Time and Events schedule (Table 1) and the Laboratory Manual. Biomarkers may include, but are not limited to, ESR, CRP and other inflammatory markers, cell surface markers, auto-antibodies such as anti-SSA, anti-SSB, RF, CD4+ T and CD19+ B cell subsets, cytokines, (including IL-2, IL-4, IL-6, IL-7, IL-10, IL12p70, IL-15, IL-17A, IL-21, IFN-γ, TGF-β, IFN- α) and other categories of biomarkers potentially involved in the development and the progression of SS.

7.8. Sample Collection and Handling

The actual dates and times of sample collection must be recorded on the laboratory requisition form.

Refer to the Time and Events Schedule (Attachment Table 2) for the timing and frequency of all sample collections. Instructions for the collection, handling, and shipment of samples are found in the laboratory manual that will be provided for sample collection and handling.

8. Study drug information

8.1. Physical Description of Study Drug

Recombinant human interleukin-2, the main ingredient of which is recombinant human interleukin-2, from recombinant Escherichia coli. The excipients are mannitol, sodium dodecyl sulfate, disodium hydrogen phosphate, sodium dihydrogen phosphate, human albumin. The placebo is the same excipients as IL-2.

8.2. Subcutaneous administration

The patients received low-dose IL-2 or placebo for 3 cycles. The specific method is 1 million units with 2 ml for injection and dissolve every other day subcutaneously (rhIL-2, 1×10^6IU, SC, Qod) for a period of 14 days. After a 14-day break, another cycle started.

8.3. Packaging

The investigational supplies will be uniquely packaged to assure that they are appropriately managed throughout the supply chain process.

8.4. Labeling

Study drug labels will contain information to meet the applicable regulatory requirements.

8.5. Preparation, Handling, and Storage

All study agent must be stored at controlled temperatures ranging from 2℃ to 8℃, not frozen, and protected from light. Vigorous shaking of the product should be avoided. Prior to administration, the product should be inspected visually for particulate matter and discoloration. If discoloration (other than a slight yellow color), visible opaque particles, or other foreign particles are observed in the solution, the product should not be used.

Aseptic procedures must be used during the preparation and administration of the study material. Exposure to direct sunlight should be avoided during preparation and administration.

8.6. Drug Accountability

The investigator is responsible for ensuring that all study drug received at the site is inventoried and accounted for throughout the study. The study drug administered

to the subject must be documented on the drug accountability form. Study-site personnel must not combine contents of the study drug containers.

Study drug must be handled in strict accordance with the protocol and the container label, and must be stored at the study site in a limited-access area or in a locked cabinet under appropriate environmental conditions. Unused study drug, and study drug returned by the subject, must be available for verification by the Sponsor's study site monitor during on-site monitoring visits. The return to the Sponsor of unused study drug, or used returned study drug for destruction, will be documented on the drug return form. When the study site is an authorized destruction unit and study drug supplies are destroyed on-site, this must also be documented on the drug return form.

Potentially hazardous materials such as used infusion bags, needles, syringes and vials should be disposed of immediately in a safe manner and therefore will not be retained for drug accountability purposes.

Study drug should be dispensed under the supervision of the investigator or a qualified member of the study-site personnel, or by a hospital/clinic pharmacist. Study drug will be supplied only to subjects participating in the study. Returned study drug must not be dispensed again, even to the same subject. Study drug may not be relabeled or reassigned for use by other subjects. The investigator agrees neither to dispense the study drug from, nor store it at, any site other than the study sites agreed upon with the Sponsor.

9. Safety Evaluations

9.1 Adverse Event

Adverse drug reactions include any symptoms, syndromes or diseases that may affect the health of patients treated with IL-2, as well as clinically relevant findings from laboratory tests or other diagnostic procedures.

The adverse reactions may be: new diseases; the deterioration of the symptoms or signs of the treatment state; accompanied by the deterioration of the disease, etc.

Severe adverse reactions refer to the following adverse events that occur at any time during the course of medication or during observation. A consequence that may endanger the patient or require action to prevent.

9.1.1 Assessment for seriousness

An adverse event is any untoward medical occurrence in a clinical study subject administered a medicinal (investigational or non-investigational) product. An adverse event does not necessarily have a causal relationship with the treatment. An adverse event can therefore be any unfavorable and unintended sign (including an abnormal finding), symptom, or disease temporally associated with the use of a medicinal

(investigational or non-investigational) product, whether or not related to that medicinal (investigational or non-investigational) product. (Definition per International Conference on Harmonisation [ICH])

This includes any occurrence that is new in onset or aggravated in severity or frequency from the baseline condition, or abnormal results of diagnostic procedures, including laboratory test abnormalities.

Note: All Adverse Events, for time of last adverse event recording.

Serious Adverse Event

A serious adverse event based on ICH and EU Guidelines on Pharmacovigilance for Medicinal Products for Human Use is any untoward medical occurrence that at any dose:

Results in death

Is life-threatening (The subject was at risk of death at the time of the event. It does not refer to an event that hypothetically might have caused death if it were more severe.)

Requires inpatient hospitalization or prolongation of existing hospitalization

Results in persistent or significant disability/incapacity

Is a congenital anomaly/birth defects.

Is a suspected transmission of any infectious agent via a medicinal product.

Is Medically Important

Medical and scientific judgment should be exercised in deciding whether expedited reporting is also appropriate in other situations, such as important medical events that may not be immediately life threatening or result in death or hospitalization but may jeopardize the subject or may require intervention to prevent one of the other outcomes listed in the definition above. These should usually be considered serious.

If a serious and unexpected adverse event occurs for which there is evidence suggesting a causal relationship between the study drug and the event (eg, death from anaphylaxis), the event must be reported as a serious and unexpected suspected adverse reaction.

Unlisted (Unexpected) Adverse Event/Reference Safety Information

An adverse event is considered unlisted if the nature or severity is not consistent with the applicable product reference safety information. For rhIL-2, the expectedness of an adverse event will be determined by whether or not it is listed in the Investigator's Brochure.

Adverse Event Associated with the Use of the Drug

An adverse event is considered associated with the use of the drug if the attribution is possible, probable, or very likely by the definitions.

9.1.2 Severity criteria

An assessment of severity grade will be made using the following general categorical descriptors:

Mild: Awareness of symptoms that are easily tolerated, causing minimal discomfort and not interfering with everyday activities.

Moderate: Sufficient discomfort is present to cause interference with normal activity.

Severe: Extreme distress, causing significant impairment of functioning or incapacitation. Prevents normal everyday activities.

The investigator should use clinical judgment in assessing the severity of events not directly experienced by the subject (eg, laboratory abnormalities).

9.1.3 Treatment measures for adverse drug reactions and related cost

The common adverse reactions of rhIL-2 in low dose injection can recover spontaneously after discontinuation.

Whole body: fever and chills are more common when IL-2 is applied at high doses, which is related to dose. Nausea, vomiting and flu-like symptoms are relatively rare. Once they appear, they can be continued to be observed and usually recover by themselves.

Injection site local: swelling, callus, pain, can continue to observe, can recover, if sustained no relief, if necessary to reduce the dose or stop medicine.

For larger doses: capillary leakage syndrome, hypotension and other manifestations, should be stopped immediately, and medication should be used when necessary to improve capillary permeability and hypotension.

Note: this study is a low-dose treatment study of rhIL-2, and the clinical application and previous research results show that no adverse reactions caused by over-dose IL-2 have occurred yet.

10. Statistical analysis

10.1. Subject Information

For all subjects who receive at least 1 dose of study drug, descriptive statistics will be provided for demographic and baseline characteristics. Efficacy analyses are done in a modified intention-to-treat(mITT) population of subjects who receive at least 1 dose of study drug. The data are also analyzed in a per-protocol population of subjects who are sufficiently compliant with treatment and do not have significant protocol violations. All subjects who are randomized and receive at least 1 dose of study agent will be included in the safety analysis.

10.2. Sample Size Determination

Sample size and power of the study was calculated based on the proportion of patients achieving primary endpoint in either group. Assuming that the proportion of patients receiving placebo group at week 12 would be 30% and the proportion of patients in low-dose IL-2 group would be 75%, 30 patients were required for each group to have 90% or higher power to detect this difference at a 5% significance level.

10.3. Efficacy Analyses

For clinical characteristics and laboratory parameters, the primary efficacy analysis is a modified intention-to-treat analysis that included all patients who are randomly assigned to this trial and underwent at least one efficacy assessment. All efficacy analyses will be performed on the per-protocol population.

10.3.1 Primary endpoint analysis

The primary endpoint was was defined as an improvement of ESSDAI to ≥ 3 points by week 24.

Logistic regression, adjusting for baseline stratifications will be used to analyzed the binary efficacy variables. Continuous variables were assessed with an analysis of covariance (ANCOVA) model, including treatment group, baseline score. For continuous variables, treatment differences across time points were evaluated using a mixed model for repeated-measures analysis, with visit, treatment group, treatment-by-visit interactions included in the model. Generalized Estimation Equations (GEE) method in a logistic repeated measures model for categorical variables, controlling for confounder variables.

A nominal significance level of 0.05 (two-sided) was applied to all the analyses.

10.3.2 Major secondary analyses

For SS, Key secondary endpoints were: dynamics of immune cell subsets including Treg and B10 cells and serum cytokines; a 30-mm or greater improvement at week 12 versus baseline on dryness VAS, pain VAS, fatigue VAS; at least 2 of 3 VAS scores, change from baseline in disease scores, including ESSDAI, dryness VAS, pain VAS, fatigue VAS, multiple fatigue index-20 (MFI-20), SF-36 physical component summary (PCS), or MCS scores, systemic manifestations, immunoglobulin A (IgA), IgG, and IgM, complement 3 and complement 4, erythrocyte sedimentation rate, ocular

parameters, including Break-Up Time, Schirmer I test, salivary gland ultrasound scan scores (SGUS)(*38*) and safety.

Continuous responses will be analyzed using an analysis of covariance model with treatment group as a fixed factor and baseline stratifications as a covariate. Nonparametric methods would be adopted when the normality assumption is violated. Logistic regression, adjusting for baseline stratifications will be used to analyzed the binary efficacy variables. Continuous variables were assessed with an analysis of covariance (ANCOVA) model, including treatment group, baseline score. For continuous variables, treatment differences across time points were evaluated using a mixed model for repeated-measures analysis, with visit, treatment group, treatment-by-visit interactions included in the model. Generalized Estimation Equations (GEE) method in a logistic repeated measures model for categorical variables, controlling for confounder variables.

10.3.2 Other planned analyses

Statistical analyses for baseline demographic and disease characteristics were done using t tests, Mann-Whitney U for comparisons of continuous variables and Chi square test for comparison of categorical variables.

Fisher's exact test will be used for discontinuations. For patients who discontinue the study at any time for any reason, the baseline data and the clinical data of each visit will be recorded.

A nominal significance level of 0.05 (two-sided) was applied to all the analyses. All statistical analyses were carried out by SPSS (version 17, IBM) and Graph Pad Prism (Version 5.0, Graph Pad Software).

10.4 Safety analyses

Safety analyses will be based on the population of subjects who receive at least 1 dose of either study agent. All reported adverse events with onset during the treatment phase will be included in the analysis. For each AE, the percentage of subjects who experience at least 1 occurrence of the given event will be summarized by treatment group. Routine safety evaluations will be performed. The incidence and types of infections and inject site reactions will be analyzed for this study.

Special attention will be given to those subjects who discontinue treatment due to an adverse event or who experience a serious adverse event.

11. Data Quality Assurance/Quality Control

Steps to be taken to ensure the accuracy and reliability of data include the selection of qualified investigators and appropriate study sites, review of protocol procedures with the investigator and study-site personnel before the study, periodic monitoring visits by the Sponsor, and direct transmission of clinical laboratory data from a central laboratory into the Sponsor's data base. Written instructions will be provided for collection, handling, storage, and shipment of samples.

Guidelines for CRF completion will be provided and reviewed with study-site personnel before the start of the study. The Sponsor will review CRFs for accuracy and completeness during on-site monitoring visits and after transmission to the Sponsor; any discrepancies will be resolved with the investigator or designee, as appropriate. After upload of the data into the study database they will be verified for accuracy and consistency with the data sources.

The unit of experimental data should be unified, such as g/l for immunoglobulin units. All data was arranged before the study.

12. Data management

The researchers should record the cases and data of the paper and electronic database in detail, accurately and timely.

13. Ethics

Approved by the ethics committee of Peking University people's hospital. All patients obtained informed consent.

14. Summary and data preservation

Keep the original records.

Make data summary according to database, statistic unit is in charge of statistic processing. The paper informed consent form and CRF form will been keep until the 5 years after the study finished.

ATTACHMENT

1. Time and Events Schedule for SS Design

		Blinded Study and follow-up									
Visit	V0	V1	V2	V3	V4	V5	V6	V7	V8	V9	V10
Week	Screening -4-0w	0w	2w±3d	4w±3d	6w ±3d	8w±3d	10w ±3d	12w ±3d	16w ±3d	20w ±3d	24w ±3d
Informed consent[a]	√										
Medical history	√										
Inclusion criteria		√									
Exclusion criteria		√									
Complete blood count[b]	√		√	√	√	√	√	√	√	√	√
Urinalysis[c]	√							√			√
Renal tubular function [d]	√							√			√
Urine HCG [e]	√										
HBV	√										
HCV	√										
Liver function [f]	√							√			√
Renal function[g]	√							√			√
Serum lipid[h]	√										
Serum glucose	√										
Electrolyte[i]	√							√			√
ESR	√	√	√	√	√	√	√	√	√	√	√
CRP	√	√	√	√	√	√	√	√	√	√	√
Ig isotype profile +C3, C4[g]	√		√	√	√	√	√	√	√	√	√
Autoantibodies[k]	√				√			√			√
Cytokines[l]		√			√			√			√
ECG	√										
Ocular examinations	√							√			√
Pulmonary function [m]	√							√			√

**Labial gland biopsy [n]**	√										
**Salivary gland ultrasonography**	√							√			√
**Collect and distribute study drug**		√	√	√	√	√	√				
**Safety assessments**		√	√	√	√	√	√	√	√	√	√
**ESSDAI score**		√						√			√
**Multidimensional Fatigue Inventory-20**		√						√			√
**VAS score**		√						√			√
**Short Form-36 Health Survey**		√						√			√
**CD4⁺T cell**		√			√			√			√
**CD8+T cell**		√			√			√			√
**Diary card （Collect, review）**		√									√

Note：

a. Screening visit must be performed no more than 4 weeks prior to the randomization visit (Week 0). Medical history included demographics, diagnosis, vital signs, whether initial treatment, family history, smoking, drinking, allergic history, major medical history, medication history, physical examination.

b. Complete blood count: hemoglobin, hematocrit, white blood cell (WBC) count with differential (basophils, eosinophils, lymphocytes, monocytes, neutrophils), platelet count.

c. Urinalysis: Red blood cells, WBC, epithelial cells, crystals or hemegranular casts, bacteria.

d. Renal tubular function: including urinarybeta-2-microglobulin (β2-MG), urinary N-acetyl-β-glucosaminidase (NAG) and retinol-binding protein(RBP).

e. Urine HCG: Urine pregnancy test.

f. Liver function: aspartate aminotransferase，alanine aminotransferase，albumin，total protein.

g. Renal function: creatinine, blood urea nitrogen (BUN), glomerular filtration rate (eGFR).

h. Serum lipid: total cholesterol, triglyceride, low density lipoprotein, high density lipoprotein.

i. Electrolyte: sodium, potassium, calcium, chloride, carbon dioxide-combining power (CO_2-CP).
j. Ig isotype profile + C3, C4: IgA, IgM, IgG, γ-globulin, C3, C4.
k. Autoantibodies: ANA, anti-SSA, anti-SSB, RF.
l. Cytokines: IL-2, TNF- α, IFN- γ, IL-6, IL-21, IL-17, TGF-β, sIL-2Rα, et al.

2. VAS score

(1) How severe has your dryness been during the last 2 weeks?

No dryness	0	1	2	3	4	5	6	7	8	9	10 Maximal imaginable dryness

(2)How severe has your fatigue been during the last 2 weeks?

No fatigue	0	1	2	3	4	5	6	7	8	9	10 Maximal imaginable fatigue

(3)How severe has your pain (joint or muscular pains in your arms or legs) been during the last 2 weeks?

No pain	0	1	2	3	4	5	6	7	8	9	10 Maximal imaginable pain

The total score is the meanscore of the 3 scales.

3. EULAR Sjögren's syndrome disease activity index (ESSDAI): Domain and item definitions and weights.

Domain [Weight]

**Constitutional [3]**<br>*Exclusion of fever of infectious origin and voluntary weight loss*		
**No = 0**	Absence of the following symptoms	☐
**Low = 1**	Mild or intermittent fever (37.5°–38.5°C)/night sweats and/or involuntary weight loss of 5 to 10% of body weight	☐
**Moderate = 2**	Severe fever (>38.5°C)/night sweats and/or involuntaryweight loss of >10% of body weight	☐

**Lymphadenopathy [4]**<br>*Exclusion of infection*		
**No = 0**	Absence of the following features	☐
**Low = 1**	Lymphadenopathy ⩾ 1 cm in any nodal region or ⩾ 2 cm in inguinal region	☐
**Moderate = 2**	Lymphadenopathy ⩾ 2 cm in any nodal region or ⩾ 3 cm in inguinal region, and/or splenomegaly (clinically palpable orassessed by imaging)	☐
**High = 3**	Current malignant B-cell proliferative disorder	☐

**Glandular [2]**<br>*Exclusion of stone or infection*		
**No = 0**	Absence of glandular swelling	☐
**Low = 1**	Small glandular swelling with enlarged parotid (⩽ 3 cm), or limited submandibular or lachrymal swelling	☐
**Moderate = 2**	Major glandular swelling with enlarged parotid (> 3 cm), orimportant submandibular or lachrymal swelling	☐

**Articular [2]**<br>*Exclusion of osteoarthritis*		
**No = 0**	Absence of currently active articular involvement	☐

**Low = 1**	Arthralgias in hands, wrists, ankles and feet accompanied bymorning stiffness (>30 min)	☐
**Moderate = 2**	1 to 5 (of 28 total count) synovitis	☐
**High = 3**	⩾ 6 (of 28 total count) synovitis	☐

**Cutaneous [3]**<br>*Rate as "No activity" stable long-lasting features related todamage*		
**No = 0**	Absence of currently active cutaneous involvement	☐
**Low = 1**	Erythema multiforma	☐
**Moderate = 2**	Limited cutaneous vasculitis, including urticarial vasculitis,or purpura limited to feet and ankle, or subacute cutaneouslupus	☐
**High = 3**	Diffuse cutaneous vasculitis, including urticarial vasculitis,or diffuse purpura, or ulcers related to vasculitis	☐

**Pulmonary [5]**<br>*Rate as "No activity" stable long-lasting features related todamage, or respiratory involvement not related to the disease(tobacco use etc.)*		
**No = 0**	Absence of currently active pulmonary involvement	☐
**Low = 1**	Persistent cough or bronchial involvement with noradiographic abnormalities on radiography<br>Or radiological or HRCT evidence of interstitial lung diseasewith: No breathlessness and normal lung function test.	☐
**Moderate = 2**	Moderately active pulmonary involvement, such asinterstitial lung disease shown by HRCT with shortness ofbreath on exercise (NHYA II) or abnormal lung functiontests restricted to: 70% >DL_{CO}≥ 40% or 80%>FVC≥60%	☐
**High = 3**	Highly active pulmonary involvement, such as interstitiallung disease shown by HRCT with shortness of breath at rest(NHYA III, IV) or with abnormal lung function tests:DL_{CO}< 40% or FVC< 60%	☐

**Renal [5]**<br>*Rate as "No activity" stable long-lasting features related todamage, and renal involvement not related to the disease.If biopsy has been performed, please rate activity basedonhistologicalfeatures first*		
**No = 0**	Absence of currently active renal involvement withproteinuria< 0.5 g/d, no hematuria, no leucocyturia, noacidosis, or long-lasting stable proteinuria due to damage	☐
**Low = 1**	Evidence of mild active renal involvement, limited to tubularacidosis without renal failure or glomerular involvementwith proteinuria (between 0.5 and 1 g/d) and without hematuria or renal failure (GFR ≥60 ml/min)	☐
**Moderate = 2**	Moderately active renal involvement, such as tubularacidosis with renal failure (GFR <60 ml/min) or glomerularinvolvement with proteinuria between 1 and 1.5 g/d and without hematuria or renal failure (GFR ≥60 ml/min) or histological evidence of extra-membranousglomerulonephritis or important interstitial lymphoidinfiltrate	☐
**High = 3**	Highly active renal involvement, such as glomerularinvolvement with proteinuria >1.5 g/d or hematuria or renalfailure (GFR <60 ml/min), or histological evidence ofproliferative glomerulonephritis or cryoglobulinemia relatedrenal involvement	☐

**Muscular [6]**<br>*Exclusion of weakness due to corticosteroids*		
**No = 0**	Absence of currently active muscular involvement	☐
**Low = 1**	Mild active myositis shown by abnormal EMG or biopsy with no weakness and creatine kinase (N <CK ≤ 2N)	☐
**Moderate = 2**	Moderately active myositis proven by abnormal EMG orbiopsy with weakness (maximal deficit of 4/5), or elevated creatine kinase (2N<CK ≤4N)	☐
**High = 3**	Highly active myositis shown by abnormal EMG or biopsy with weakness (deficit ≤ 3/5) or elevated creatine kinase (>4N)	☐

**PNS[5]**<br>*Rate as "No activity" stable long-lasting features related todamage or PNS involvement not related to the disease*

**No = 0**	Absence of currently active PNS involvement	☐
**Low = 1**	Mild active peripheral nervous system involvement, such aspure sensory axonal polyneuropathy shown by NCS ortrigeminal (V) neuralgia	☐
**Moderate = 2**	Moderately active peripheral nervous system involvementshown by NCS, such as axonal sensory-motor neuropathywith maximal motor deficit of 4/5, pure sensory neuropathywith presence of cryoglobulinemic vasculitis,ganglionopathy with symptoms restricted to mild/moderateataxia, inflammatory demyelinating polyneuropathy (CIDP)with mild functional impairment (maximal motor deficit of4/5or mild ataxia),<br>Or cranial nerve involvement of peripheral origin (except trigeminal (V) neralgia)	☐
**High = 3**	Highly active PNS involvement shown by NCS, such as axonal sensory-motor neuropathy with motor deficit ≤3/5, peripheral nerve involvement due to vasculitis(mononeuritis multiplex etc.), severe ataxia due toganglionopathy, inflammatory demyelinatingpolyneuropathy (CIDP) with severe functional impairment: motor deficit ≤3/5 or severe ataxia	☐

**<u>CNS[5]</u>**<br>*Rate as “No activity” stable long-lasting features related todamage or CNS involvement not related to the disease*		
**No = 0**	Absence of currently active CNS involvement	☐
**Low = 1**	Moderately active CNS features, such as cranial nerveinvolvement of central origin, optic neuritis or multiplesclerosis-like syndrome with symptoms restricted to puresensory impairment or proven cognitive impairment	☐
**High = 3**	Highly active CNS features, such as cerebral vasculitis withcerebrovascular accident or transient ischemic attack,seizures, transverse myelitis, lymphocytic meningitis,multiple sclerosis-like syndrome with motor deficit.	☐

**Hematological [2]**<br>*For anemia, neutropenia, and thrombopenia, only auto-immunecytopenia must be considered*<br>*Exclusion of vitamin or iron deficiency, drug-induced cytopenia*		
**No = 0**	Absence of auto-immune cytopenia	☐
**Low = 1**	Cytopenia of auto-immune origin with neutropenia (1000 <neutrophils < 1500/mm3), and/or anemia (10 < hemoglobin< 12 g/dl), and/or thrombocytopenia (100,000 < platelets <150,000/mm3)<br>Or lymphopenia (500 < lymphocytes < 1000/mm3)	☐
**Moderate = 2**	Cytopenia of auto-immune origin with neutropenia (500 ⩽ neutrophils ⩽ 1000/mm3), and/or anemia (8 ⩽ hemoglobin ⩽ 10 g/dl), and/or thrombocytopenia (50,000 ⩽ platelets ⩽ 100,000/mm3)<br>Or lymphopenia (⩽500/mm3)	☐
**High = 3**	Cytopenia of auto-immune origin with neutropenia(neutrophils < 500/mm3), and/or or anemia (hemoglobin <8 g/dl) and/or thrombocytopenia (platelets <50,000/mm3)	☐

**Biological [1]**		
**No = 0**	Absence of any of the following biological feature	☐
**Low = 1**	Clonal component and/or hypocomplementemia (low C4 orC3 or CH50) and/or hypergammaglobulinemia or high IgGlevel between 16 and 20 g/L	☐
**Moderate = 2**	Presence of cryoglobulinemia and/orhypergammaglobulinemia or high IgG level > 20 g/L, and/or recent onset hypogammaglobulinemia or recent decreaseof IgG level (<5 g/L)	☐

CIDP= chronic inflammatory demyelinating polyneuropathy; CK= creatine kinase; CNS= central nervous system; DLCO= diffusing CO capacity;EMG= electromyogram; FVC= forced vital capacity; GFR= glomerular filtration rate; Hb= hemoglobin; HRCT= high-resolution computedtomography; IgG= immunoglobulin G; NCS= nerve conduction studies; NHYA= New York heart association

4. MOS 36-Item Short Form Survey Instrument (SF-36)

Choose one option for each questionnaire item.

1. In general, would you say your health is:

① Excellent ② Very good ③ Good ④ Fair ⑤ Poor

2. Compared to one year ago, how would you rate your health in general now?

① Much better now than one year ago

② Somewhat better now than one year ago

③ About the same

④ Somewhat worse now than one year ago

⑤ Much worse now than one year ago

(The score is 1, 2, 3, 4, 5 for ①-⑤)

3. The following items are about activities you might do during a typical day. Does your health now limit you in these activities? If so, how much?

(1) Vigorous activities, such as running, lifting heavy objects, participating in strenuous sports

①Yes, limited a lot ②Yes, limited a little ③No, not limited at all

(The score is 1, 2, 3 for①-③the same below)

(2) Moderate activities, such as moving a table, pushing a vacuum cleaner, bowling, or playing golf

①Yes, limited a lot ②Yes, limited a little ③No, not limited at all

(3) Lifting or carrying groceries

①Yes, limited a lot ②Yes, limited a little ③No, not limited at all

(4) Climbing several flights of stairs

①Yes, limited a lot ②Yes, limited a little ③No, not limited at all

(5) Climbing one flight of stairs

①Yes, limited a lot ②Yes, limited a little ③No, not limited at all

(6) Bending, kneeling, or stooping

①Yes, limited a lot ②Yes, limited a little ③No, not limited at all

(7) Walking more than a mile

①Yes, limited a lot ②Yes, limited a little ③No, not limited at all

(8) Walking several blocks

①Yes, limited a lot ②Yes, limited a little ③No, not limited at all

(9) Walking one block

①Yes, limited a lot ②Yes, limited a little ③No, not limited at all

(10) Bathing or dressing yourself

①Yes, limited a lot ②Yes, limited a little ③No, not limited at all

4. During the past 4 weeks, have you had any of the following problems with your work or other regular daily activities as a result of your physical health?

(1) Cut down the amount of time you spent on work or other activities

①Yes ②No

(The score is 1, 2 for ①-②the same below)

(2) Accomplished less than you would like

①Yes ②No

(3) Were limited in the kind of work or other activities

①Yes ②No

(4) Had difficulty performing the work or other activities (for example, it took extra effort)

①Yes ②No

5. During the past 4 weeks, have you had any of the following problems with your work or other regular daily activities as a result of any emotional problems (such as feeling depressed or anxious)?

(1) Cut down the amount of time you spent on work or other activities

①Yes ②No

(The score is 1, 2 for ①-② the same below)

(2) Accomplished less than you would like

①Yes ②No

(3) Didn't do work or other activities as carefully as usual

①Yes ②No

6. During the past 4 weeks, to what extent has your physical health or emotional problems interfered with your normal social activities with family, friends, neighbors, or groups?

①Not at all ②Slightly③Moderately ④ Quite a bit ⑤Extremely

(The score is 5, 4, 3, 2, 1 for ①-⑤)

7. How much bodily pain have you had during the past 4 weeks?

①None ②Very mild ③Mild ④Moderate ⑤ sever ⑥Very severe

(The score is 6, 5.4, 4.2, 3.1, 2.2, 1 for ①-⑤)

8. During the past 4 weeks, how much did pain interfere with your normal work (including both work outside the home and housework)?

①Not at all ② A little bit ③Moderately ④Quite a bit ⑤Extremely

(If neither 7 nor 8 is yes, the score is 6, 4.75, 3.5, 2.25, 1.0 for ①-⑤;if 7 is yes and 8 is no, the score is 5, 4, 3, 2, 1 for ①-⑤)

9. These questions are about how you feel and how things have been with you during the past 4 weeks. For each question, please give the one answer that comes closest to the way you have been feeling.

How much of the time during the past 4 weeks...

(1) Did you feel full of pep?

①All of the time ② Most of the time ③A good bit of the time ④Some of the time ⑤A little of the time ⑥None of the time

(The score is 6, 5, 4, 3, 2, 1 for ①-⑥)

(2) Have you been a very nervous person?

①All of the time ② Most of the time ③A good bit of the time ④Some of the time ⑤A little of the time ⑥None of the time

(The score is 1, 2, 3, 4, 5, 6 for ①-⑥)

(3) Have you felt so down in the dumps that nothing could cheer you up?

①All of the time ② Most of the time ③A good bit of the time ④Some of the time ⑤A little of the time ⑥None of the time

(The score is 1, 2, 3, 4, 5, 6 for ①-⑥)

(4) Have you felt calm and peaceful?

①All of the time ② Most of the time ③A good bit of the time ④Some of the time ⑤A little of the time ⑥None of the time

(The score is 6, 5, 4, 3, 2, 1 for ①-⑥)

(5) Did you have a lot of energy?

①All of the time ② Most of the time ③A good bit of the time ④Some of the time ⑤A little of the time ⑥None of the time

(The score is 6, 5, 4, 3, 2, 1 for ①-⑥)

(6) Have you felt downhearted and blue?

①All of the time ② Most of the time ③A good bit of the time ④Some of the time ⑤A little of the time ⑥None of the time

(7) Did you feel worn out?

①All of the time ② Most of the time ③A good bit of the time ④Some of the time ⑤A little of the time ⑥None of the time

(The score is 1, 2, 3, 4, 5, 6 for ①-⑥)

(8) Have you been a happy person?

①All of the time ② Most of the time ③A good bit of the time ④Some of the time ⑤A little of the time ⑥None of the time

(The score is 6, 5, 4, 3, 2, 1 for ①-⑥)

(9) Did you feel tired?

①All of the time ② Most of the time ③A good bit of the time ④Some of the time ⑤A little of the time ⑥None of the time

(The score is 1, 2, 3, 4, 5, 6 for ①-⑥)

10. During the past 4 weeks, how much of the time has your physical health or emotional problems interfered with your social activities (like visiting with friends, relatives, etc.)?

①All of the time ② Most of the time ③A good bit of the time ④Some of the time ⑤A little of the time ⑥None of the time

(The score is 1, 2, 3, 4, 5, 6 for ①-⑥)

11. How TRUE or FALSE is each of the following statements for you.

(1) I seem to get sick a little easier than other people

① Definitely true ② Mostly true ③ Don't know ④ Mostly false ⑤Definitely false

(The score is 1, 2, 3, 4, 5 for ①-⑤)

(2) I am as healthy as anybody I know

①Definitely true ②Mostly true ③Don't know ④Mostly false ⑤ Definitely false

(The score is 5, 4, 3, 2, 1 for ①-⑤)

(3) I expect my health to get worse

①Definitely true ②Mostly true ③Don't know ④Mostly false ⑤ Definitely false

(The score is 1, 2, 3, 4, 5 for ①-⑤)

(4) My health is excellent
①Definitely true ②Mostly true ③Don't know ④Mostly false ⑤ Definitely false
(The score is 5, 4, 3, 2, 1 for ①-⑤)

SF-36 score calculation

Item	Score	Item	Score	Item	Score	Item	Score
1		3-(9)		7		9-(9)	
2		3-(10)		8		10	
3-(1)		4-(1)		9-(1)		11-(1)	
3-(2)		4-(2)		9-(2)		11-(2)	
3-(3)		4-(3)		9-(3)		11-(3)	
3-(4)		4-(4)		9-(4)		11-(4)	
3-(5)		5-(1)		9-(5)			
3-(6)		5-(2)		9-(6)			
3-(7)		5-(3)		9-(7)			
3-(8)		6		9-(8)		Sum	

5.Multidimensional fatigue inventory-20, MFI-20

No.	Item	Yes 1 2 3 4 5 No
1	I feel fit	□□□□□
2	Physically I feel only able to doa little	□□□□□
3	I feel very active	□□□□□
4	I feel like doing all sorts of nicethings	□□□□□
5	I feel tired	□□□□□
6	I think I do a lot in a day	□□□□□
7	When I am doing something, Ican keep my thoughts on it	□□□□□
8	Physically I can take on a lot	□□□□□
9	I dread having to do things	□□□□□
10	I think I do very little in a day	□□□□□
11	I can concentrate well	□□□□□
12	I am rested	□□□□□
13	It takes a lot of effort toconcentrate on things	□□□□□
14	Physically I feel I am in a badcondition	□□□□□
15	I have a lot of plans	□□□□□
16	I tire easily	□□□□□
17	I get little done	□□□□□
18	I don’t feel like doing anything	□□□□□
19	My thoughts easily wander	□□□□□
20	Physically I feel I am in anexcellent condition	□□□□□

- Item 2, 5, 9, 10, 13, 14, 16, 17, 18, 19 were positively formulated.
- Item 1, 3, 4, 6, 7, 8, 11, 12, 15, 20 were negatively formulated.

Higher scores indicate a higher degree of fatigue

Reference

1. Reksten TR, Jonsson MV, Szyszko EA, Brun JG, Jonsson R, Brokstad KA. Cytokine and autoantibody profiling related to histopathological features in primary Sjogren's syndrome. Rheumatology (Oxford). 2009; **48**(9): 1102-6.

2. Ramos-Casals M, Brito-Zeron P, Siso-Almirall A, Bosch X, Tzioufas AG. Topical and systemic medications for the treatment of primary Sjogren's syndrome. Nature reviews Rheumatology. 2012; **8**(7): 399-411.

3. Saadoun D, Rosenzwajg M, Joly F, Six A, Carrat F, Thibault V, et al. Regulatory T-cell responses to low-dose interleukin-2 in HCV-induced vasculitis. The New England journal of medicine. 2011; **365**(22): 2067-77.

4. Hartemann A, Bensimon G, Payan CA, Jacqueminet S, Bourron O, Nicolas N, et al. Low-dose interleukin 2 in patients with type 1 diabetes: a phase 1/2 randomised, double-blind, placebo-controlled trial. The lancet Diabetes & endocrinology. 2013; **1**(4): 295-305.

5. Vitali C, Bombardieri S, Jonsson R, Moutsopoulos HM, Alexander EL, Carsons SE, et al. Classification criteria for Sjogren's syndrome: a revised version of the European criteria proposed by the American-European Consensus Group. Annals of the rheumatic diseases. 2002; **61**(6): 554-8.

6. Seror R, Ravaud P, Mariette X, Bootsma H, Theander E, Hansen A, et al. EULAR Sjogren's Syndrome Patient Reported Index (ESSPRI): development of a consensus patient index for primary Sjogren's syndrome. Annals of the rheumatic diseases. 2011; **70**(6): 968-72.

7. Seror R, Ravaud P, Bowman SJ, Baron G, Tzioufas A, Theander E, et al. EULAR Sjogren's syndrome disease activity index: development of a consensus systemic disease activity index for primary Sjogren's syndrome. Annals of the rheumatic diseases. 2010; **69**(6): 1103-9.

8. Smets EM, Garssen B, Bonke B, De Haes JC. The Multidimensional Fatigue Inventory (MFI) psychometric qualities of an instrument to assess fatigue. Journal of psychosomatic research. 1995; **39**(3): 315-25.

9. Ware JE, Jr., Sherbourne CD. The MOS 36-item short-form health survey (SF-36). I. Conceptual framework and item selection. Medical care. 1992; **30**(6): 473-83.

Clinical Protocol

Low-dose Interleukin-2 treatment of active Sjögren's syndrome: a randomized, double-blind and placebo-controlled clinical trial

Research Institution： Peking University People's Hospital

2015/12/21

Contents

1. Title....4
2. Abstract....4
3. Background....4
4. Objectives....4
5. Study Design....5
5.1. Sjögren's Syndrome (SS)....5
Experimental group（n=30）： original treatment plan + rhIL-2....5
Control group（n=30）： original treatment plan + placebo....5
5.2. Procedures for Blinding....5
5.3. Emergency Unblinding....5
5.4. Drug Allocation....5
5.5. Concomitant Therapy Requirement. 5
5.6. Study Population....5
5.7. Inclusion and Exclusion Criteria:....6
5.8 Subject withdrawal or discontinuation....8
6. Study Drug Information....9
6.1. Drugs....9
6.2. Dose and administration....9
6.3. Concomitant therapy....9
6.3.5. Topical Medications....10
7. Study Procedures....10
7.1. Screening Phase....10
7.2. Double-Blind Treatment Phase....11
7.3. Follow-up Phase....11
7.4. Subjects Withdrawing from Study Participation....11
7.5. Efficacy Evaluations....11
7.6. Follow-up....12
7.7. Biomarkers....13
8. Study drug information....14
8.1. Physical Description of Study Drug....14
8.2. Subcutaneous administration....14
8.5. Preparation, Handling, and Storage....14
8.6. Drug Accountability....15
9. Safety Evaluations....15
9.1 Adverse Event....15
9.1.1 Assessment for seriousness....16
10. Statistical analysis....18
10.1. Subject Information....18
10.2. Sample Size Determination....18
10.3. Efficacy Analyses....18

10.4 Safety analyses 20
11. Data Quality Assurance/Quality Control........ 20
12. Data management........ 20
13. Ethics 21
14. Summary and data preservation 21
ATTACHMENT 22
1.Time and Events Schedule for SS Design 22
2. VAS score........ 25
3. EULAR Sjögren's syndrome disease activity index (ESSDAI): Domain and item definitions and weights. 26
4. MOS 36-Item Short Form Survey Instrument (SF-36)........ 31
5. Multidimensional fatigue inventory-20, MFI-20 35

1. Title

Low-dose Interleukin-2 treatment of active Sjögren’s syndrome: a randomized, double-blind and placebo-controlled clinical trial.

2. Abstract

Low-dose recombinant human interleukin-2 (rhIL-2, trade name: Xinjier) has been reported to be effective in the treatment of various autoimmune or inflammation-related diseases in a series of pilot open-label clinical studies, but its real effects on autoimmune diseases has not been confirmed in clinical trials with strict control groups. This study aims to confirm and clarify the safety and efficacy of rhIL-2 for Sjögren’s syndrome (SS) in a randomized, double-blind and placebo-controlled clinical trial, and further evaluate the effect of the treatment on immune cell subsets to explore the immune mechanism of treatment.

3. Background

Primary Sjögren’s syndrome (pSS) is a chronic autoimmune disease characterized by xerostomia, xerophthalmia and multiple organ involvement, including peripheral neuropathy, lymphadenopathy, renal tubular acidosis and interstitial lung disease. Recent studies have demonstrated that dysregulation of T cells and B cells is critical in the development of pSS. The production of multiple autoantibodies is indicative of the loss of B cell tolerance. Currently available treatment for SS is symptomatic and empirical. Sicca manifestations are treated through administration of topical therapies, such as saliva substitutes and artificial tears. Patients with systemic damage are generally treated with corticosteroids in combination with immunosuppressive agents This project aims to confirm the efficacy and safety of rhIL-2 in the treatment of SS.

4. Objectives

The study aims to clarify the safety and efficacy of low-dose rhIL-2 in the treatment of SS, and further explore the immune mechanism of low-dose IL-2 treatment.

5. Study Design

5.1. Sjögren's Syndrome (SS)

Adjust the drug treatment for active SS based on the original treatment as follows:

Experimental group（n=30）： original treatment plan + rhIL-2

Control group（n=30）： original treatment plan + placebo

5.2. Procedures for Blinding

We designed a double-blind study. There was no difference in the drug packaging or dosage between recombinant human rhIL-2 and placebo. Independent third party handle the blind bottom generation and on-site blinding. Using SAS 9.3 statistical software to generate one random coding tables in a ratio of 1:1, namely SS01-60. The selected subjects were randomly assigned to the experimental group or the control group.

5.3. Emergency Unblinding

Each subject will have an emergency unblinded envelope in which the specific medication used by the subject is recorded and can only be opened when necessary. Emergency envelopes are kept by researchers responsible for this trial. The main investigator must be notified before opening the envelope, and the emergency envelope can be opened after approval. After unblinding, the subject will be discontinued from further administration of study agent and will be treated as a shedding case.

5.4. Drug Allocation

Subjects who meet the inclusion criteria, according to their indications, will be provided with the drugs by the drug administrators in the order of enrollment time. The drug will also be provided in the order of the drug number from small to large.

5.5. Concomitant Therapy Requirement.

The original treatment regimen included glucocorticosteroid with or without immunosuppressive agents, in which prednisone acetate was initiated in the dosage of <10mg/d.

5.6. Study Population

Sixty cases of SS, either outpatient or inpatient.

5.7. Inclusion and Exclusion Criteria:

5.7.1 Inclusion Criteria Applicable to All Subjects

Male or female 18-70 years of age at time of screening.

Diagnosis of primary Sjögren's syndrome (according to the 2002 AECG classification criteria)[5] for ≥3 months before screening.

European League Against Rheumatism Sjögren's Syndrome Disease Activity Index (ESSDAI) ≥5.

Positive tests for RF, anti-SSA or anti-SSB antibodies*.

Patients with a diagnosis of primary Sjögren's syndrome with more than 10 years disease duration must have at least one systemic feature of following items,

Hypergamaglobulinaemia* (IgG over 16.8g/L [Upper limit of normal level]) or Low complement C3 / C4*, or Cryoglobulinaemia*

OR

Active/past history since diagnosis of the following (ascribed to Sjögren's syndrome)

Purpura / cutaneous vasculitis,

Lymphadenopathy,

Persistent parotid salivary gland swelling not due to infection,

Peripheral neuropathy

(previously documented by nerve conduction tests),

Interstitial lung disease confirmed by HRCT,

Renal tubular acidosis requiring treatment,

CNS disease ascribed to Sjögren's syndrome (confirmed by MRI),

Myositis (CK>2 times the ULN) and EMG or biopsy evidence of myositis,

Inflammatory arthritis.

An unstimulated salivary flow rate greater than 0 ml in 15 minutes.

Symptomatic oral dryness (≥ 50/100 on VAS).

Symptomatic fatigue (≥ 50/100 on VAS).

Symptomatic pain (≥ 50/100 on VAS).

Patients on corticosteroids (≤7.5 mg/d prednisone or equivalent), DMARDs (e.g. methotrexate, hydroxychloroquine, azathioprine, MMF, leflunomide, ciclosporin etc.), or pilocarpine*** must have been on a stable dose for 4 weeks prior to receiving the first infusion of study medication and expected to remain on this dose throughout the study. If they have stopped any of these drugs they should have been off it for at least 4 weeks prior to receiving study medication.

Given their written informed consent to participate in the trial and expected to be able to adhere to the study visit schedule and other protocol requirements.

*Anti-Ro antibody test, IgG, RF, C3/C4 and Cryoglobulinaemia assays performed within 3 months of screening may be used to confirm eligibility. If greater than 3 months repeats should be performed locally at screening to confirm eligibility.

VAS range 0-100mm with 100mm corresponding to worst severity.

***Pilocarpine or drugs with similar pharmacological action should not be used within 12 hours of the assessment visits at screening, baseline, week 12, week 24 (end of study).

5.7.2 Exclusion Criteria

Patients will be excluded from the trial for the following reasons:

Diagnosis of Secondary Sjögren’s syndrome.

Any AECG exclusion criteria not covered elsewhere (graft versus host disease, primary lymphoma excluding PSS, sarcoidosis).

Stable disease activity (IgG ＜16.8 g/L) at the time of enrolment.

Prior rituximab or monoclonal antibody usage.

Severe comorbidities: including

Heart failure (≥ grade III NYHA)

Renal insufficiency (creatinine clearance ≤30 ml/min);

Hepatic insufficiency (serum ALT or AST >3 times the ULN, or total bilirubin >ULN for the central laboratory conducting the test).

Other severe, progressive or uncontrolled hematologic, gastrointestinal, endocrine, pulmonary, cardiac, neurologic, or cerebral disease (including demyelinating diseases such as multiple sclerosis).

Known allergies, hypersensitivity, or intolerance to IL-2 or its excipients.

History of severe allergic reaction to monoclonal antibodies or to murine, chimeric, or human proteins or their excipients.

Had a severe infection (including, but not limited to hepatitis, pneumonia, sepsis, or pyelonephritis); had been hospitalized for an infection; or had been treated with IV antibiotics for an infection, within 2 months prior to the first administration of study agent.

Chest radiograph within 3 months prior to the first administration of study agent that showed an abnormality suggestive of a malignancy or current active infection, including TB.

Had a nontuberculous mycobacterial infection or opportunistic infection (e.g., cytomegalovirus, pneumocystosis, aspergillosis) within 6 months prior to screening.

Infected with HIV (positive serology for HIV antibody) or hepatitis C (positive serology for Hep C antibody). If seropositive, consultation with a physician with expertise in the treatment of HIV or hepatitis C virus infection was recommended.

Infected with hepatitis B virus. For patients who were not eligible for this study due to hepatitis B virus test results, consultation with a physician with expertise in the treatment of hepatitis B virus infection was recommended.

Had any known malignancy or has a history of malignancy within the previous 5 years (with the exception of a nonmelanoma skin cancer that had been treated with no evidence of recurrence for ≥3 months before the first study agent administration or cervical neoplasia with surgical cure).

Had uncontrolled psychiatric or emotional disorder, including a history of drug and alcohol abuse within the past 3 years that might prevent the successful completion of the study.

Received, or was expected to receive, any live virus or bacterial vaccination within 3 months before the first administration of study agent, during the study, or within 4 months after the last administration of study agent. Had a BCG vaccination within 12 months of screening.

Pregnancy, lactation or women of child-bearing potential (WCBP) unwilling to use medically approved contraception whilst receiving treatment and for 12 months after treatment has finished.

Men whose partners are of child-bearing potential but who are unwilling to use appropriate medically approved contraception whilst receiving treatment and for 12 months after treatment has finished.

5.8 Subject withdrawal or discontinuation

Discontinuation of Study Treatment

A subject will be withdrawn from the study for any of the following reasons:

An AE temporally associated with study agent infusion or injection

Withdrawal of consent

Participation being deemed unlikely compliant with medical follow-up or study visit schedule

Malignancy

Pregnancy or planning to become pregnant within the study period

The initiation of prohibited medications or treatments

Lost to follow-up

Death

If a subject is lost to follow-up, every reasonable effort must be made by the study site personnel to contact the subject and determine the reason for withdrawal. The measures taken to follow-up must be documented. When a subject withdraws before completing the study, the reason for withdrawal is to be documented in the CRF and in the source document. Study drug assigned to the withdrawn subject may not be assigned to another subject. Subjects who withdraw from this study will not be replaced. A subject who withdraws from the study will have the following options regarding the optional research samples:

The collected samples will be retained and used in accordance with the subject's original informed consent for optional research samples.

The subject may withdraw consent for optional research samples, in which case the samples will be destroyed and no further testing will be needed.

6. Study Drug Information

6.1. Drugs

Recombinant human Interleukin-2, placebo

6.2. Dose and administration

The patients received low-dose IL-2 or placebo for 3 cycles. The method is 1 million units subcutaneously administrated every other day (rhIL-2 1×106U, SC, Qod) for a period of 14 days. After a 14-day break, another cycle started. All the patients will be treated for the first 12 weeks which included three treatment cycles with IL-2 or placebo and followed up for further 12 weeks without study medicine.

6.3. Concomitant therapy

All pre-study therapies administered up to 30 days before entry into screening must be recorded at screening. All concomitant therapies must be recorded throughout the study and any changes must be recorded throughout the study. Every reasonable effort should be made to keep concomitant medications stable through Week 24 or early termination.

6.3.1 Immunomodulators

If receiving immunomodulators, subjects should be receiving stable dosing from screening through Week 24 or early termination. A reduction in immunomodulators is allowed only if the subject develops unacceptable side effects, with the implication that this may affect interpretation of the subjects' clinical data.

6.3.2 Antimalarial Medications

Stable treatment with hydroxychloroquine is permitted through the trial.

6.3.3 Corticosteroid Therapy

Unnecessary dose changes are discouraged, and any dose adjustments should be made in increments. Changes in corticosteroids are allowed for medical necessity, but the degree and timing of the adjustment should be carefully considered as this may have an impact on the study results.

The original treatment regimen included glucocorticosteroid with or without immunosuppressive agents, in which prednisone acetate was initiated in the dosage of <7.5mg/d.

If subjects experience a worsening in their disease activity while tapering corticosteroids, further dose decreases may be suspended, and/or their oral corticosteroid dose may be temporarily increased if deemed necessary by the investigator.

6.3.4 Nonsteroidal Anti-inflammatory Drugs

Subjects treated with NSAIDs, including aspirin and selective cyclooxygenase-2 (COX-2) inhibitors, and other analgesics should receive the usual marketed doses approved in the country in which the study is being conducted. Prescriptions of NSAIDs and other regularly administered analgesics should not be adjusted for at least 2 weeks prior to the first administration of the study drug and through Week 24, and may be changed only if the subject develops unacceptable side effects. The addition of new NSAIDs to the treatment regimen is not permitted during the period between 0 and 12 weeks. Minor adjustments in NSAID therapy are allowed after Week 12 although it is recommended that the use of any NSAIDS remain as stable as possible, and any notable changes should be recorded.

6.3.5. Topical Medications

Topical medications are permitted; however, topical compounds cannot include a prohibited medication. Topical ointments or creams of cyclosporine A are prohibited through Week 24; however ophthalmic use is permitted. Topical ophthalmic drugs can be used, such as sodium hyaluronate eye drops for dry eyes and deproteinized calf blood extract eye gel. Low potency topical steroids are allowed except on day of study visit. Medium to high potency topical corticosteroids are not allowed during the study. For 72 hours prior to study visit, topical medications should not be applied to lesions under evaluation.

7. Study Procedures

7.1. Screening Phase

Written informed consent must be obtained and reviewed by investigator before any screening data is collected. The screening visit must be performed no more than 4 weeks prior to the randomization visit (Week 0). In addition, to be eligible for study participation, subjects must have received approval for study randomization following review and adjudication of screening SS.

Repeat or unscheduled samples may be taken for safety reasons or for technical issues with the collection or analysis of specific samples.

All screening evaluations establishing subject eligibility will be performed and reviewed by investigator before subject can be randomized. All enrollment subjects must meet the classification criteria for these diseases.

7.2. Double-Blind Treatment Phase

7.2.1. Week 0/Day of Randomization

At Week 0, eligible subjects will be randomly assigned in a 1:1 ratio to receive either recombinant human Interleukin-2 or placebo in a blinded manner.

7.2.2. Placebo-controlled Treatment Period

The patients were treated with low-dose rhIL-2 or placebo in the same way.

Low-dose IL-2 treated with a 12-week treatment including 3 treatment cycles. IL-2 at a dose of 1 million IU or placebo was administered subcutaneously every other day for 2 weeks, followed by a 2-week break in each treatment cycle.

7.3. Follow-up Phase

The observation period was 12-week treatment and the 12-week follow-up. The clinical manifestations, autoantibodies and other related laboratory tests were collected, the patients with SS were assessed with ESSDAI score assess the degree of remission of their condition. And adverse drug reactions were monitored before and after different courses of treatment.

7.4. Subjects Withdrawing from Study Participation

Subjects who withdraw from study participation will not be required to return for any follow-up assessments.

7.5. Efficacy Evaluations

Efficacy evaluations will be done based on the primary and secondary outcome measures (see section 7.5.3). A complete list describing all efficacy evaluations and endpoints, and which evaluations are included in the composite endpoints is provided in Attachment table 1 & 2. All efficacy evaluations should be consistently performed by the study investigator to achieve comparable measures over time. These data will be reviewed at every visit that these data are collected and may require reconciliation of inconsistencies across assessments.

7.5.1. Endpoints

Primary Endpoint

The primary endpoint was defined as an improvement of ESSDAI to ≥ 3 points by week 24

Major Secondary Endpoints

The secondary endpoints included other clinical responses, safety and changes of

immune cell subsets including T cell and B cell subsets.

7.6. Follow-up

7.6.1. Baseline follow-up

(1) General information: including name, sex, age, contact number, course of disease.

(2) Clinical symptoms and signs: including rash, fever, oral ulcer, foamy urine, disease activity score and so on.

(3) Laboratory examination: blood routine examination, liver and kidney function, ANA, anti-SSA, anti-SSB, RF, immunoglobulin, ESR, CRP, T cell and B cell subsets, IL-2, TGF-12β and other cytokines.

7.6.2. Follow up visits

Follow up once every 2 weeks in the first 3 months (6 times), and then follow up once a month:

(1) According to disease activity score, including ESSDAI score, to observe whether the clinical symptoms are relieved.

(2) Monitoring of changes in serological indicators： Every 4 weeks, blood routine examination, liver and kidney function, according to the disease related autoantibodies such as anti-SSA, anti-SSB, RF, immunoglobulin, ESR, CRP to evaluate the condition.

(3) Laboratory flow detection: At week 0, 2, 4, 6, 8, 12 16, 20 and 24, CD4+ T and CD19+ B cells were detected by flow cytometry, to evaluate the changes of cell group ratio before and after treatment.

(4) Cytokines: the levels of IL-2, IL-6, TGF-β and other cytokines were detected by ELISA at 0 and 12 weeks.

7.6.3. Evaluations

A complete list describing all efficacy evaluations and endpoints, and which evaluations are included in the composite endpoints is provided in Attachment 2.

7.6.3.1. ESSDAI

The European League Against Rheumatism (EULAR) organized an international collaboration of global SS experts in 2010 to develop two recognized disease activity indicators. That is, the SS patient reporting index (EULAR Sjögren’s syndrome patient reported index) based on the patient's subjective symptoms and the SS disease activity index based on the patient's objective systemic symptoms. (EULAR Sjögren’s syndrome syndrome disease activity index, ESSDAI).

At screening (Attachment 6), ESSDAI is an index of disease activity that covers 12 aspects of the body, including systemic conditions, lymphadenopathy, glands, skin, joints, lungs, kidneys, muscles, peripheral nervous systems, the central nervous system, blood system and biochemistry. Each aspect is divided into 3 ~ 4 levels according to its

activity degree, and the weight of each aspect is obtained by multivariate linear regression model. Score only the symptoms associated with the disease to avoid the effects of long-term clinical symptoms. The final score ranges from 0 to 123, and 0 indicates disease-free activity.

7.6.3.2. Fatigue, pain and dryness VAS score

Visual analogue scale VASs (scores range from 0 [none] to 100mm [worst] for dryness, pain, and fatigue).

7.6.3.3. MFI-20

The questionnaire comprises 20 items for which the person must specify the extent to which the particular statements relates to him/her on a five-point scale, ranging from “Yes”, that is true or “No”, that is not true. An equal number of items is worded in a positive and in a negative direction to counteract response tendencies. Higher scores indicate a higher degree of fatigue.

7.6.3.4. Short-Form-36

The RAND short-form (SF)-36 questionnaire is a self-administered multi-domain scale with 36 items. Eight health domains cover a range of functioning (Attachment 3):

-Limitations in physical function

-Limitations in usual role activities

-Bodily pain

-General mental health (psychological distress and well-being)

-Vitality (energy and fatigue)

-Limitations in social functioning due to physical or mental health problems

-Limitations in usual role activities due to personal or emotional problems General health perception

-The subscales are scored from 0 to 100

The scoring yields a Physical Component Summary score and a Mental Component Summary score, a total score, and subscale scores. Higher scores represent better outcomes. It is appropriate for persons over the age of 14 and may be completed in 5 to 10 minutes. Translations are available in most languages; the instrument has undergone extensive linguistic and cultural validation.

The concepts measured by the SF-36 are not specific to any age, disease[10], or treatment group, allowing comparison of relative burden of different diseases and the benefit of different treatments. A change of 3 points in any of the subscales or 5 points for the component score is associated with clinically meaningful change.

7.7. Biomarkers

The collection, preparation, storage and shipment of blood, serum and urine are detailed in the Time and Events schedule (Table 1) and the Laboratory Manual. Biomarkers may include, but are not limited to, ESR, CRP and other inflammatory

markers, cell surface markers, auto-antibodies such as anti-SSA, anti-SSB, RF, CD4+ T and CD19+ B cell subsets, cytokines, (including IL-2, IL-4, IL-6, IL-7, IL-10, IL12p70, IL-15, IL-17A, IL-21, IFN-γ, TGF-β, IFN- α) and other categories of biomarkers potentially involved in the development and the progression of SS.

7.8. Sample Collection and Handling

The actual dates and times of sample collection must be recorded on the laboratory requisition form.

Refer to the Time and Events Schedule (Attachment Table 2) for the timing and frequency of all sample collections. Instructions for the collection, handling, and shipment of samples are found in the laboratory manual that will be provided for sample collection and handling.

8. Study drug information

8.1. Physical Description of Study Drug

Recombinant human interleukin-2, the main ingredient of which is recombinant human interleukin-2, from recombinant Escherichia coli. The excipients are mannitol, sodium dodecyl sulfate, disodium hydrogen phosphate, sodium dihydrogen phosphate, human albumin. The placebo is the same excipients as IL-2.

8.2. Subcutaneous administration

The patients received low-dose IL-2 or placebo for 3 cycles. The specific method is 1 million international units with 2 ml for injection and dissolve every other day subcutaneously (rhIL-2, 1×10^6IU, SC, Qod) for a period of 14 days. After a 14-day break, another cycle started.

8.3. Packaging

The investigational supplies will be uniquely packaged to assure that they are appropriately managed throughout the supply chain process.

8.4. Labeling

Study drug labels will contain information to meet the applicable regulatory requirements.

8.5. Preparation, Handling, and Storage

All study agent must be stored at controlled temperatures ranging from 2℃ to 8℃, not frozen, and protected from light. Vigorous shaking of the product should be

avoided. Prior to administration, the product should be inspected visually for particulate matter and discoloration. If discoloration (other than a slight yellow color), visible opaque particles, or other foreign particles are observed in the solution, the product should not be used.

Aseptic procedures must be used during the preparation and administration of the study material. Exposure to direct sunlight should be avoided during preparation and administration.

8.6. Drug Accountability

The investigator is responsible for ensuring that all study drug received at the site is inventoried and accounted for throughout the study. The study drug administered to the subject must be documented on the drug accountability form. Study-site personnel must not combine contents of the study drug containers.

Study drug must be handled in strict accordance with the protocol and the container label, and must be stored at the study site in a limited-access area or in a locked cabinet under appropriate environmental conditions. Unused study drug, and study drug returned by the subject, must be available for verification by the Sponsor's study site monitor during on-site monitoring visits. The return to the Sponsor of unused study drug, or used returned study drug for destruction, will be documented on the drug return form. When the study site is an authorized destruction unit and study drug supplies are destroyed on-site, this must also be documented on the drug return form.

Potentially hazardous materials such as used infusion bags, needles, syringes and vials should be disposed of immediately in a safe manner and therefore will not be retained for drug accountability purposes.

Study drug should be dispensed under the supervision of the investigator or a qualified member of the study-site personnel, or by a hospital/clinic pharmacist. Study drug will be supplied only to subjects participating in the study. Returned study drug must not be dispensed again, even to the same subject. Study drug may not be relabeled or reassigned for use by other subjects. The investigator agrees neither to dispense the study drug from, nor store it at, any site other than the study sites agreed upon with the Sponsor.

9. Safety Evaluations

9.1 Adverse Event

Adverse drug reactions include any symptoms, syndromes or diseases that may affect the health of patients treated with IL-2, as well as clinically relevant findings from laboratory tests or other diagnostic procedures.

The adverse reactions may be: new diseases; the deterioration of the symptoms or signs of the treatment state; accompanied by the deterioration of the disease, etc.

Severe adverse reactions refer to the following adverse events that occur at any time during the course of medication or during observation. A consequence that may endanger the patient or require action to prevent.

9.1.1 Assessment for seriousness

An adverse event is any untoward medical occurrence in a clinical study subject administered a medicinal (investigational or non-investigational) product. An adverse event does not necessarily have a causal relationship with the treatment. An adverse event can therefore be any unfavorable and unintended sign (including an abnormal finding), symptom, or disease temporally associated with the use of a medicinal (investigational or non-investigational) product, whether or not related to that medicinal (investigational or non-investigational) product. (Definition per International Conference on Harmonisation [ICH])

This includes any occurrence that is new in onset or aggravated in severity or frequency from the baseline condition, or abnormal results of diagnostic procedures, including laboratory test abnormalities.

Note: All Adverse Events, for time of last adverse event recording.

Serious Adverse Event

A serious adverse event based on ICH and EU Guidelines on Pharmacovigilance for Medicinal Products for Human Use is any untoward medical occurrence that at any dose:

Results in death

Is life-threatening (The subject was at risk of death at the time of the event. It does not refer to an event that hypothetically might have caused death if it were more severe.)

Requires inpatient hospitalization or prolongation of existing hospitalization

Results in persistent or significant disability/incapacity

Is a congenital anomaly/birth defects.

Is a suspected transmission of any infectious agent via a medicinal product.

Is Medically Important

Medical and scientific judgment should be exercised in deciding whether expedited reporting is also appropriate in other situations, such as important medical events that may not be immediately life threatening or result in death or hospitalization but may jeopardize the subject or may require intervention to prevent one of the other outcomes listed in the definition above. These should usually be considered serious.

If a serious and unexpected adverse event occurs for which there is evidence suggesting a causal relationship between the study drug and the event (eg, death from anaphylaxis), the event must be reported as a serious and unexpected suspected adverse reaction.

Unlisted (Unexpected) Adverse Event/Reference Safety Information

An adverse event is considered unlisted if the nature or severity is not consistent with the applicable product reference safety information. For rhIL-2, the expectedness of an adverse event will be determined by whether or not it is listed in the Investigator's Brochure.

Adverse Event Associated with the Use of the Drug

An adverse event is considered associated with the use of the drug if the attribution is possible, probable, or very likely by the definitions.

9.1.2 Severity criteria

An assessment of severity grade will be made using the following general categorical descriptors:

Mild: Awareness of symptoms that are easily tolerated, causing minimal discomfort and not interfering with everyday activities.

Moderate: Sufficient discomfort is present to cause interference with normal activity.

Severe: Extreme distress, causing significant impairment of functioning or incapacitation. Prevents normal everyday activities.

The investigator should use clinical judgment in assessing the severity of events not directly experienced by the subject (eg, laboratory abnormalities).

9.1.3 Treatment measures for adverse drug reactions and related cost

The common adverse reactions of rhIL-2 in low dose injection can recover spontaneously after discontinuation.

Whole body: fever and chills are more common when IL-2 is applied at high doses, which is related to dose. Nausea, vomiting and flu-like symptoms are relatively rare. Once they appear, they can be continued to be observed and usually recover by themselves.

Injection site local: swelling, callus, pain, can continue to observe, can recover, if sustained no relief, if necessary to reduce the dose or stop medicine.

For larger doses: capillary leakage syndrome, hypotension and other manifestations, should be stopped immediately, and medication should be used when necessary to improve capillary permeability and hypotension.

Note: this study is a low-dose treatment study of rhIL-2, and the clinical application and previous research results show that no adverse reactions caused by over-dose IL-2 have occurred yet.

10. Statistical analysis

10.1. Subject Information

For all subjects who receive at least 1 dose of study drug, descriptive statistics will be provided for demographic and baseline characteristics. Efficacy analyses are done in a modified intention-to-treat(mITT) population of subjects who receive at least 1 dose of study drug. The data are also analyzed in a per-protocol population of subjects who are sufficiently compliant with treatment and do not have significant protocol violations. All subjects who are randomized and receive at least 1 dose of study agent will be included in the safety analysis.

10.2. Sample Size Determination

Sample size and power of the study was calculated based on the proportion of patients achieving primary endpoint in either group. Assuming that the proportion of patients receiving placebo group at week 12 would be 30% and the proportion of patients in low-dose IL-2 group would be 75%, 30 patients were required for each group to have 90% or higher power to detect this difference at a 5% significance level.

10.3. Efficacy Analyses

For clinical characteristics and laboratory parameters, the primary efficacy analysis is a modified intention-to-treat analysis that included all patients who are randomly assigned to this trial and underwent at least one efficacy assessment. All efficacy analyses will be performed on the per-protocol population.

10.3.1 Primary endpoint analysis

The primary endpoint was was defined as an improvement of ESSDAI to ≥ 3 points by week 24.

Logistic regression, adjusting for baseline stratifications will be used to analyzed the binary efficacy variables. Continuous variables were assessed with an analysis of covariance (ANCOVA) model, including treatment group, baseline score. For continuous variables, treatment differences across time points were evaluated using a mixed model for repeated-measures analysis, with visit, treatment group, treatment-by-visit interactions included in the model. Generalized Estimation Equations (GEE) method in a logistic repeated measures model for categorical variables, controlling for confounder variables.

A nominal significance level of 0.05 (two-sided) was applied to all the analyses.

10.3.2 Major secondary analyses

For SS, Key secondary endpoints were: dynamics of immune cell subsets including Treg and B10 cells and serum cytokines; a 30-mm or greater improvement at week 12 versus baseline on dryness VAS, pain VAS, fatigue VAS; at least 2 of 3 VAS scores, change from baseline in disease scores, including ESSDAI, dryness VAS, pain VAS, fatigue VAS, multiple fatigue index-20 (MFI-20), SF-36 physical component summary (PCS), or MCS scores, systemic manifestations, immunoglobulin A (IgA), IgG, and IgM, complement 3 and complement 4, erythrocyte sedimentation rate, ocular parameters, including Break-Up Time, Schirmer I test, salivary gland ultrasound scan scores (SGUS)(*38*) and safety.

Continuous responses will be analyzed using an analysis of covariance model with treatment group as a fixed factor and baseline stratifications as a covariate. Nonparametric methods would be adopted when the normality assumption is violated. Logistic regression, adjusting for baseline stratifications will be used to analyzed the binary efficacy variables. Continuous variables were assessed with an analysis of covariance (ANCOVA) model, including treatment group, baseline score. For continuous variables, treatment differences across time points were evaluated using a mixed model for repeated-measures analysis, with visit, treatment group, treatment-by-visit interactions included in the model. Generalized Estimation Equations (GEE) method in a logistic repeated measures model for categorical variables, controlling for confounder variables.

10.3.2 Other planned analyses

Statistical analyses for baseline demographic and disease characteristics were done using t tests, Mann-Whitney U for comparisons of continuous variables and Chi square test for comparison of categorical variables.

Fisher's exact test will be used for discontinuations. For patients who discontinue the study at any time for any reason, the baseline data and the clinical data of each visit will be recorded.

A nominal significance level of 0.05 (two-sided) was applied to all the analyses. All statistical analyses were carried out by SPSS (version 17, IBM) and Graph Pad Prism (Version 5.0, Graph Pad Software).

10.4 Safety analyses

Safety analyses will be based on the population of subjects who receive at least 1 dose of either study agent. All reported adverse events with onset during the treatment phase will be included in the analysis. For each AE, the percentage of subjects who experience at least 1 occurrence of the given event will be summarized by treatment group. Routine safety evaluations will be performed. The incidence and types of infections and inject site reactions will be analyzed for this study.

Special attention will be given to those subjects who discontinue treatment due to an adverse event or who experience a serious adverse event.

11. Data Quality Assurance/Quality Control

Steps to be taken to ensure the accuracy and reliability of data include the selection of qualified investigators and appropriate study sites, review of protocol procedures with the investigator and study-site personnel before the study, periodic monitoring visits by the Sponsor, and direct transmission of clinical laboratory data from a central laboratory into the Sponsor's data base. Written instructions will be provided for collection, handling, storage, and shipment of samples.

Guidelines for CRF completion will be provided and reviewed with study-site personnel before the start of the study. The Sponsor will review CRFs for accuracy and completeness during on-site monitoring visits and after transmission to the Sponsor; any discrepancies will be resolved with the investigator or designee, as appropriate. After upload of the data into the study database they will be verified for accuracy and consistency with the data sources.

The unit of experimental data should be unified, such as g/l for immunoglobulin units. All data was arranged before the study.

12. Data management

The researchers should record the cases and data of the paper and electronic database in detail, accurately and timely.

13. Ethics

Approved by the ethics committee of Peking University people's hospital. All patients obtained informed consent.

14. Summary and data preservation

Keep the original records.

Make data summary according to database, statistic unit is in charge of statistic processing. The paper informed consent form and CRF form will been keep until the 5 years after the study finished.

ATTACHMENT

1.Time and Events Schedule for SS Design

		Blinded Study and follow-up									
**Visit**	**V0**	**V1**	**V2**	**V3**	**V4**	**V5**	**V6**	**V7**	**V8**	**V9**	**V10**
**Week**	**Screening -4-0w**	**0w**	**2w±3d**	**4w±3d**	**6w ±3d**	**8w±3d**	**10w ±3d**	**12w ±3d**	**16w ±3d**	**20w ±3d**	**24w ±3d**
**Informed consent**[a]	√										
**Medical history**	√										
**Inclusion criteria**		√									
**Exclusion criteria**		√									
**Complete blood count** [b]	√		√	√	√	√	√	√	√	√	√
**Urinalysis** [c]	√							√			√
**Renal tubular function** [d]	√							√			√
**Urine HCG** [e]	√										
**HBV**	√										
**HCV**	√										
**Liver function** [f]	√							√			√
**Renal function** [g]	√							√			√
**Serum lipid** [h]	√										
**Serum glucose**	√										
**Electrolyte** [i]	√							√			√
**ESR**	√							√			√
**CRP**	√							√			√
**Ig isotype profile +C3, C4** [g]	√		√	√	√	√	√	√	√	√	√
**Autoantibodies** [k]	√				√			√			√
**Cytokines** [l]		√						√			
**ECG**	√										
**Ocular examinations**	√							√			√
**Pulmonary function** [m]	√							√			√

**Labial gland biopsy** [n]	√										
**Salivary gland ultrasonography**	√							√			√
**Collect and distribute study drug**		√	√	√	√	√	√				
**Safety assessments**		√	√	√	√	√	√	√	√	√	√
**ESSDAI score**		√						√			√
**Multidimensional Fatigue Inventory-20**		√						√			√
**VAS score**		√						√			√
**Short Form-36 Health Survey**		√						√			√
**$CD4^+$T cell**		√	√	√	√	√	√	√	√	√	√
**CD19+B cell**		√	√	√	√	√	√	√	√	√	√
**Diary card （Collect, review）**		√									√

Note：

a. Screening visit must be performed no more than 4 weeks prior to the randomization visit (Week 0). Medical history included demographics, diagnosis, vital signs, whether initial treatment, family history, smoking, drinking, allergic history, major medical history, medication history, physical examination.

b. Complete blood count: hemoglobin, hematocrit, white blood cell (WBC) count with differential (basophils, eosinophils, lymphocytes, monocytes, neutrophils), platelet count.

c. Urinalysis: Red blood cells, WBC, epithelial cells, crystals or hemegranular casts, bacteria.

d. Renal tubular function: including urinary beta-2-microglobulin (β2-MG), urinary N-acetyl-β-glucosaminidase (NAG) and retinol-binding protein (RBP), measure when necessary.

e. Urine HCG: Urine pregnancy test: If a woman is capable of pregnancy, she must check it.

f. Liver function: aspartate aminotransferase，alanine aminotransferase，albumin，total protein.
g. Renal function: creatinine, blood urea nitrogen (BUN), glomerular filtration rate (eGFR).
h. Serum lipid: total cholesterol, triglyceride, low density lipoprotein, high density lipoprotein.
i. Electrolyte: sodium, potassium, calcium, chloride, carbon dioxide-combining power (CO_2-CP).
j. Ig isotype profile + C3, C4: IgA, IgM, IgG, γ-globulin, C3, C4.
k. Autoantibodies: ANA, anti-SSA, anti-SSB, RF.
l. Cytokines: IL-2, TNF-α, IFN-γ, IL-6, IL-21, IL-17, TGF-β, sIL-2Rα, et al.
m. Pulmonary function: when necessary.
n. Labial gland biopsy: when necessary.

2. VAS score

(1) How severe has your dryness been during the last 2 weeks?

No dryness	0	1	2	3	4	5	6	7	8	9	10	Maximal imaginable dryness

(2)How severe has your fatigue been during the last 2 weeks?

No fatigue	0	1	2	3	4	5	6	7	8	9	10	Maximal imaginable fatigue

(3)How severe has your pain (joint or muscular pains in your arms or legs) been during the last 2 weeks?

No pain	0	1	2	3	4	5	6	7	8	9	10	Maximal imaginable pain

The total score is the mean score of the 3 scales.

3. EULAR Sjögren's syndrome disease activity index (ESSDAI): Domain and item definitions and weights.

Domain [Weight]

**Constitutional [3]**<br>*Exclusion of fever of infectious origin and voluntary weight loss*		
**No = 0**	Absence of the following symptoms	☐
**Low = 1**	Mild or intermittent fever (37.5°–38.5°C)/night sweats and/or involuntary weight loss of 5 to 10% of body weight	☐
**Moderate = 2**	Severe fever (>38.5°C)/night sweats and/or involuntary weight loss of >10% of body weight	☐

**Lymphadenopathy [4]**<br>*Exclusion of infection*		
**No = 0**	Absence of the following features	☐
**Low = 1**	Lymphadenopathy ≥ 1 cm in any nodal region or ≥ 2 cm in inguinal region	☐
**Moderate = 2**	Lymphadenopathy ≥ 2 cm in any nodal region or ≥ 3 cm in inguinal region, and/or splenomegaly (clinically palpable or assessed by imaging)	☐
**High = 3**	Current malignant B-cell proliferative disorder	☐

**Glandular [2]**<br>*Exclusion of stone or infection*		
**No = 0**	Absence of glandular swelling	☐
**Low = 1**	Small glandular swelling with enlarged parotid (≤ 3 cm), or limited submandibular or lachrymal swelling	☐
**Moderate = 2**	Major glandular swelling with enlarged parotid (> 3 cm), or important submandibular or lachrymal swelling	☐

**Articular [2]**<br>*Exclusion of osteoarthritis*		
**No = 0**	Absence of currently active articular involvement	☐

**Low = 1**	Arthralgias in hands, wrists, ankles and feet accompanied by morning stiffness (>30 min)	☐
**Moderate = 2**	1 to 5 (of 28 total count) synovitis	☐
**High = 3**	⩾ 6 (of 28 total count) synovitis	☐

**Cutaneous [3]**<br>*Rate as "No activity" stable long-lasting features related to damage*		
**No = 0**	Absence of currently active cutaneous involvement	☐
**Low = 1**	Erythema multiforma	☐
**Moderate = 2**	Limited cutaneous vasculitis, including urticarial vasculitis, or purpura limited to feet and ankle, or subacute cutaneous lupus	☐
**High = 3**	Diffuse cutaneous vasculitis, including urticarial vasculitis, or diffuse purpura, or ulcers related to vasculitis	☐

**Pulmonary [5]**<br>*Rate as "No activity" stable long-lasting features related to damage, or respiratory involvement not related to the disease (tobacco use etc.)*		
**No = 0**	Absence of currently active pulmonary involvement	☐
**Low = 1**	Persistent cough or bronchial involvement with no radiographic abnormalities on radiography<br>Or radiological or HRCT evidence of interstitial lung disease with: No breathlessness and normal lung function test.	☐
**Moderate = 2**	Moderately active pulmonary involvement, such as interstitial lung disease shown by HRCT with shortness of breath on exercise (NHYA II) or abnormal lung function tests restricted to: 70% >DL_{CO}≥ 40% or 80%>FVC≥60%	☐
**High = 3**	Highly active pulmonary involvement, such as interstitial lung disease shown by HRCT with shortness of breath at rest (NHYA III, IV) or with abnormal lung function tests: DL_{CO}< 40% or FVC< 60%	☐

**Renal [5]**<br>*Rate as "No activity" stable long-lasting features related to damage, and renal involvement not related to the disease. If biopsy has been performed, please rate activity based onhistologicalfeatures first*		
**No = 0**	Absence of currently active renal involvement with proteinuria< 0.5 g/d, no hematuria, no leucocyturia, no acidosis, or long-lasting stable proteinuria due to damage	☐
**Low = 1**	Evidence of mild active renal involvement, limited to tubular acidosis without renal failure or glomerular involvement with proteinuria (between 0.5 and 1 g/d) and without hematuria or renal failure (GFR ⩾60 ml/min)	☐
**Moderate = 2**	Moderately active renal involvement, such as tubular acidosis with renal failure (GFR <60 ml/min) or glomerular involvement with proteinuria between 1 and 1.5 g/d and without hematuria or renal failure (GFR ⩾60 ml/min) or histological evidence of extra-membranous glomerulonephritis or important interstitial lymphoid infiltrate	☐
**High = 3**	Highly active renal involvement, such as glomerular involvement with proteinuria >1.5 g/d or hematuria or renal failure (GFR <60 ml/min), or histological evidence of proliferative glomerulonephritis or cryoglobulinemia related renal involvement	☐

**Muscular [6]**<br>*Exclusion of weakness due to corticosteroids*		
**No = 0**	Absence of currently active muscular involvement	☐
**Low = 1**	Mild active myositis shown by abnormal EMG or biopsy with no weakness and creatine kinase (N <CK ⩽ 2N)	☐
**Moderate = 2**	Moderately active myositis proven by abnormal EMG or biopsy with weakness (maximal deficit of 4/5), or elevated creatine kinase (2N<CK ⩽4N)	☐
**High = 3**	Highly active myositis shown by abnormal EMG or biopsy with weakness (deficit ⩽ 3/5) or elevated creatine kinase (>4N)	☐

**PNS [5]**<br>*Rate as "No activity" stable long-lasting features related to damage or PNS involvement not related to the disease*		
**No = 0**	Absence of currently active PNS involvement	☐
**Low = 1**	Mild active peripheral nervous system involvement, such as pure sensory axonal polyneuropathy shown by NCS or trigeminal (V) neuralgia	☐
**Moderate = 2**	Moderately active peripheral nervous system involvement shown by NCS, such as axonal sensory-motor neuropathy with maximal motor deficit of 4/5, pure sensory neuropathy with presence of cryoglobulinemic vasculitis, ganglionopathy with symptoms restricted to mild/moderate ataxia, inflammatory demyelinating polyneuropathy (CIDP) with mild functional impairment (maximal motor deficit of 4/5or mild ataxia),<br>Or cranial nerve involvement of peripheral origin (except trigeminal (V) neralgia)	☐
**High = 3**	Highly active PNS involvement shown by NCS, such as axonal sensory-motor neuropathy with motor deficit ≤3/5, peripheral nerve involvement due to vasculitis (mononeuritis multiplex etc.), severe ataxia due to ganglionopathy, inflammatory demyelinating polyneuropathy (CIDP) with severe functional impairment: motor deficit ≤3/5 or severe ataxia	☐

**CNS [5]**<br>*Rate as "No activity" stable long-lasting features related to damage or CNS involvement not related to the disease*		
**No = 0**	Absence of currently active CNS involvement	☐
**Low = 1**	Moderately active CNS features, such as cranial nerve involvement of central origin, optic neuritis or multiple sclerosis-like syndrome with symptoms restricted to pure sensory impairment or proven cognitive impairment	☐
**High = 3**	Highly active CNS features, such as cerebral vasculitis with cerebrovascular accident or transient ischemic attack, seizures, transverse myelitis, lymphocytic meningitis, multiple sclerosis-like syndrome with motor deficit.	☐

**Hematological [2]**<br>*For anemia, neutropenia, and thrombopenia, only auto-immune cytopenia must be considered*<br>*Exclusion of vitamin or iron deficiency, drug-induced cytopenia*		
**No = 0**	Absence of auto-immune cytopenia	☐
**Low = 1**	Cytopenia of auto-immune origin with neutropenia (1000 < neutrophils < 1500/mm3), and/or anemia (10 < hemoglobin < 12 g/dl), and/or thrombocytopenia (100,000 < platelets < 150,000/mm3)<br>Or lymphopenia (500 < lymphocytes < 1000/mm3)	☐
**Moderate = 2**	Cytopenia of auto-immune origin with neutropenia (500 ⩽ neutrophils ⩽ 1000/mm3), and/or anemia (8 ⩽ hemoglobin ⩽ 10 g/dl), and/or thrombocytopenia (50,000 ⩽ platelets ⩽ 100,000/mm3)<br>Or lymphopenia (⩽500/mm3)	☐
**High = 3**	Cytopenia of auto-immune origin with neutropenia (neutrophils < 500/mm3), and/or or anemia (hemoglobin < 8 g/dl) and/or thrombocytopenia (platelets <50,000/mm3)	☐

**Biological [1]**		
**No = 0**	Absence of any of the following biological feature	☐
**Low = 1**	Clonal component and/or hypocomplementemia (low C4 or C3 or CH50) and/or hypergammaglobulinemia or high IgG level between 16 and 20 g/L	☐
**Moderate = 2**	Presence of cryoglobulinemia and/or hypergammaglobulinemia or high IgG level > 20 g/L, and/ or recent onset hypogammaglobulinemia or recent decrease of IgG level (<5 g/L)	☐

CIDP= chronic inflammatory demyelinating polyneuropathy; CK= creatine kinase; CNS= central nervous system; DLCO= diffusing CO capacity; EMG= electromyogram; FVC= forced vital capacity; GFR= glomerular filtration rate; Hb= hemoglobin; HRCT= high-resolution computed tomography; IgG= immunoglobulin G; NCS= nerve conduction studies; NHYA= New York heart association

4. MOS 36-Item Short Form Survey Instrument (SF-36)

Choose one option for each questionnaire item.

1. In general, would you say your health is:

① Excellent ② Very good ③ Good ④ Fair ⑤ Poor

2. Compared to one year ago, how would you rate your health in general now?

① Much better now than one year ago

② Somewhat better now than one year ago

③ About the same

④ Somewhat worse now than one year ago

⑤ Much worse now than one year ago

(The score is 1, 2, 3, 4, 5 for ①-⑤)

3. The following items are about activities you might do during a typical day. Does your health now limit you in these activities? If so, how much?

(1) Vigorous activities, such as running, lifting heavy objects, participating in strenuous sports

① Yes, limited a lot ② Yes, limited a little ③ No, not limited at all

(The score is 1, 2, 3 for ①-③the same below)

(2) Moderate activities, such as moving a table, pushing a vacuum cleaner, bowling, or playing golf

① Yes, limited a lot ② Yes, limited a little ③ No, not limited at all

(3) Lifting or carrying groceries

① Yes, limited a lot ② Yes, limited a little ③ No, not limited at all

(4) Climbing several flights of stairs

① Yes, limited a lot ② Yes, limited a little ③ No, not limited at all

(5) Climbing one flight of stairs

① Yes, limited a lot ② Yes, limited a little ③ No, not limited at all

(6) Bending, kneeling, or stooping

① Yes, limited a lot ② Yes, limited a little ③ No, not limited at all

(7) Walking more than a mile

① Yes, limited a lot ② Yes, limited a little ③ No, not limited at all

(8) Walking several blocks

① Yes, limited a lot ② Yes, limited a little ③ No, not limited at all

(9) Walking one block

① Yes, limited a lot ② Yes, limited a little ③ No, not limited at all

(10) Bathing or dressing yourself

① Yes, limited a lot ② Yes, limited a little ③ No, not limited at all

4. During the past 4 weeks, have you had any of the following problems with your work or other regular daily activities as a result of your physical health?

(1) Cut down the amount of time you spent on work or other activities

① Yes ② No

(The score is 1, 2 for ①-② the same below)

(2) Accomplished less than you would like

① Yes ② No

(3) Were limited in the kind of work or other activities

① Yes ② No

(4) Had difficulty performing the work or other activities (for example, it took extra effort)

① Yes ② No

5. During the past 4 weeks, have you had any of the following problems with your work or other regular daily activities as a result of any emotional problems (such as feeling depressed or anxious)?

(1) Cut down the amount of time you spent on work or other activities

① Yes ② No

(The score is 1, 2 for ①-② the same below)

(2) Accomplished less than you would like

① Yes ② No

(3) Didn't do work or other activities as carefully as usual

① Yes ② No

6. During the past 4 weeks, to what extent has your physical health or emotional problems interfered with your normal social activities with family, friends, neighbors, or groups?

① Not at all ② Slightly ③ Moderately ④ Quite a bit ⑤ Extremely

(The score is 5, 4, 3, 2, 1 for ①-⑤)

7. How much bodily pain have you had during the past 4 weeks?

① None ② Very mild ③ Mild ④ Moderate ⑤ sever ⑥ Very severe

(The score is 6, 5.4, 4.2, 3.1, 2.2, 1 for ①-⑤)

8. During the past 4 weeks, how much did pain interfere with your normal work (including both work outside the home and housework)?

① Not at all ② A little bit ③ Moderately ④ Quite a bit ⑤ Extremely

(If neither 7 nor 8 is yes, the score is 6, 4.75, 3.5, 2.25, 1.0 for ①-⑤; if 7 is yes and 8 is no, the score is 5, 4, 3, 2, 1 for ①-⑤)

9. These questions are about how you feel and how things have been with you during the past 4 weeks. For each question, please give the one answer that comes closest to the way you have been feeling.

How much of the time during the past 4 weeks...

(1) Did you feel full of pep?

① All of the time ② Most of the time ③ A good bit of the time ④ Some of the time ⑤ A little of the time ⑥ None of the time

(The score is 6, 5, 4, 3, 2, 1 for ①-⑥)

(2) Have you been a very nervous person?

① All of the time ② Most of the time ③ A good bit of the time ④ Some of the time ⑤ A little of the time ⑥ None of the time

(The score is 1, 2, 3, 4, 5, 6 for ①-⑥)

(3) Have you felt so down in the dumps that nothing could cheer you up?

① All of the time ② Most of the time ③ A good bit of the time ④ Some of the time ⑤ A little of the time ⑥ None of the time

(The score is 1, 2, 3, 4, 5, 6 for ①-⑥)

(4) Have you felt calm and peaceful?

① All of the time ② Most of the time ③ A good bit of the time ④ Some of the time ⑤ A little of the time ⑥ None of the time

(The score is 6, 5, 4, 3, 2, 1 for ①-⑥)

(5) Did you have a lot of energy?

① All of the time ② Most of the time ③ A good bit of the time ④ Some of the time ⑤ A little of the time ⑥ None of the time

(The score is 6, 5, 4, 3, 2, 1 for ①-⑥)

(6) Have you felt downhearted and blue?

① All of the time ② Most of the time ③ A good bit of the time ④ Some of the time ⑤ A little of the time ⑥ None of the time

(7) Did you feel worn out?

① All of the time ② Most of the time ③ A good bit of the time ④ Some of the time ⑤ A little of the time ⑥ None of the time

(The score is 1, 2, 3, 4, 5, 6 for ①-⑥)

(8) Have you been a happy person?

① All of the time ② Most of the time ③ A good bit of the time ④ Some of the time ⑤ A little of the time ⑥ None of the time

(The score is 6, 5, 4, 3, 2, 1 for ①-⑥)

(9) Did you feel tired?

① All of the time ② Most of the time ③ A good bit of the time ④ Some of the time ⑤ A little of the time ⑥ None of the time

(The score is 1, 2, 3, 4, 5, 6 for ①-⑥)

10. During the past 4 weeks, how much of the time has your physical health or emotional problems interfered with your social activities (like visiting with friends, relatives, etc.)?

① All of the time ② Most of the time ③ A good bit of the time ④ Some of the time ⑤ A little of the time ⑥ None of the time

(The score is 1, 2, 3, 4, 5, 6 for ①-⑥)

11. How TRUE or FALSE is each of the following statements for you.

(1) I seem to get sick a little easier than other people

① Definitely true ② Mostly true ③ Don't know ④ Mostly false ⑤ Definitely false

(The score is 1, 2, 3, 4, 5 for ①-⑤)

(2) I am as healthy as anybody I know

① Definitely true ② Mostly true ③ Don't know ④ Mostly false ⑤ Definitely false

(The score is 5, 4, 3, 2, 1 for ①-⑤)

(3) I expect my health to get worse

① Definitely true ② Mostly true ③ Don't know ④ Mostly false ⑤ Definitely false

(The score is 1, 2, 3, 4, 5 for ①-⑤)

(4) My health is excellent

① Definitely true ② Mostly true ③ Don't know ④ Mostly false ⑤ Definitely false

(The score is 5, 4, 3, 2, 1 for ①-⑤)

SF-36 score calculation

Item	Score	Item	Score	Item	Score	Item	Score
1		3-(9)		7		9-(9)	
2		3-(10)		8		10	
3-(1)		4-(1)		9-(1)		11-(1)	
3-(2)		4-(2)		9-(2)		11-(2)	
3-(3)		4-(3)		9-(3)		11-(3)	
3-(4)		4-(4)		9-(4)		11-(4)	
3-(5)		5-(1)		9-(5)			
3-(6)		5-(2)		9-(6)			
3-(7)		5-(3)		9-(7)			
3-(8)		6		9-(8)		Sum	

5. Multidimensional fatigue inventory-20, MFI-20

No.	Item	Yes 1 2 3 4 5 No
1	I feel fit	☐ ☐ ☐ ☐ ☐
2	Physically I feel only able to do a little	☐ ☐ ☐ ☐ ☐
3	I feel very active	☐ ☐ ☐ ☐ ☐
4	I feel like doing all sorts of nice things	☐ ☐ ☐ ☐ ☐
5	I feel tired	☐ ☐ ☐ ☐ ☐
6	I think I do a lot in a day	☐ ☐ ☐ ☐ ☐
7	When I am doing something, I can keep my thoughts on it	☐ ☐ ☐ ☐ ☐
8	Physically I can take on a lot	☐ ☐ ☐ ☐ ☐
9	I dread having to do things	☐ ☐ ☐ ☐ ☐
10	I think I do very little in a day	☐ ☐ ☐ ☐ ☐
11	I can concentrate well	☐ ☐ ☐ ☐ ☐
12	I am rested	☐ ☐ ☐ ☐ ☐
13	It takes a lot of effort to concentrate on things	☐ ☐ ☐ ☐ ☐
14	Physically I feel I am in a bad condition	☐ ☐ ☐ ☐ ☐
15	I have a lot of plans	☐ ☐ ☐ ☐ ☐
16	I tire easily	☐ ☐ ☐ ☐ ☐
17	I get little done	☐ ☐ ☐ ☐ ☐
18	I don’t feel like doing anything	☐ ☐ ☐ ☐ ☐
19	My thoughts easily wander	☐ ☐ ☐ ☐ ☐
20	Physically I feel I am in an excellent condition	☐ ☐ ☐ ☐ ☐

- Item 2, 5, 9, 10, 13, 14, 16, 17, 18, 19 were positively formulated.
- Item 1, 3, 4, 6, 7, 8, 11, 12, 15, 20 were negatively formulated.

Higher scores indicate a higher degree of fatigue

PROTOCOL AMENDMENTS

Protocol Version

Original Protocol 22 Oct. 2014

Final Protocol 21 Dec. 2015

The final protocol was used to conduct the study in place of any preceding version of the protocol.

Applicable section(s)	Description of change(s)
**Endpoints**	The secondary endpoints included other clinical responses, safety and changes of immune cell subsets including ~~Treg, Tfh, Th17 cell and CD8+T cells.~~ **CD4 and CD19 B cells.**
**Follow up visits**	Laboratory flow detection: ~~At week 0, 6, 12 and 24, CD4+T and CD8+T cells~~ **At week 0, 2, 4, 6, 8, 10, 12, 16, 20 and 24, CD4+T and CD8+T cells sbutypes** were detected by flow cytometry, to evaluate the changes of cell group ratio before and after treatment.
	Cytokines:the levels of IL-2, IL-6,TGF-β and other cytokines were detected by ELISA at 0, ~~6,~~ 12 ~~and 24~~ weeks.
**Time and events schedule for SS design**	In the schedule form:<br>ESR: V0, ~~V2,~~ V4, ~~V5, V6,~~ V7, ~~V8, V9,~~ V10.<br>CRP: V0, ~~V2,~~ V4, ~~V5, V6,~~ V7, ~~V8, V9,~~ V10.<br>Cytokines:V1, ~~V4,~~ V7, ~~V10.~~<br>~~CD8+T cell,~~ **CD19+B cell**<br>CD4+T cell: V1, **V2, V3,** V4, **V5, V6,** V7, **V8, V9,** V10.<br>CD19+B cell: V1, **V2, V3,** V4, **V5, V6,** V7, **V8, V9,** V10.

**Time and events schedule for SS design**	Note:<br>d Renal tubular function: including urinarybeta-2-microglobulin (β2-MG), urinary N-acetyl-β-glucosaminidase (NAG) and retinol-binding protein(RBP), **measure when necessary**.<br><br>e Urine HCG: Urine pregnancy test**:** If a woman is capable of pregnancy, she must check it.<br><br>**m. Pulmonary function: when necessary.**<br><br>**n. Labial gland biopsy: when necessary.**

研究方案

低剂量白介素-2治疗活动性干燥综合征：

随机、双盲、安慰剂对照临床试验

研究单位：北京大学人民医院

二O一四年十一月二十九日

目录

目录......2
1. 题目......4
2. 项目摘要......4
3. 研究背景......4
4. 研究目的......4
5. 研究设计......4
5.1. 干燥综合征......4
5.2. 盲法的实施......4
5.3. 紧急揭盲......4
5.4. 药物分配方法......5
5.5. 合并用药规定......5
5.6. 研究人群......5
5.7. 入选和排除标准......5
5.7.1. 入选标准......5
5.7.2. 排除标准......6
5.8. 退出标准......7
6. 试验药物信息......7
6.1. 药物......7
6.2. 剂量和用法......7
6.3. 伴随治疗......8
6.3.1. 免疫调节剂......8
6.3.2. 抗疟药......8
6.3.3. 糖皮质激素治疗......8
6.3.4. 非甾体类抗炎药......8
6.3.5. 外用药......8
7. 研究过程......8
7.1. 筛选阶段......8
7.2. 双盲治疗阶段......9
7.2.1. 第 0 周/随机分组日......9
7.2.2. 安慰剂对照的治疗阶段......9
7.3. 随访阶段......9
7.4. 撤出研究的受试者......9
7.5. 有效性评估......9
7.5.1. 终点......9
7.6. 随访......10
7.6.1. 基线随访......10
7.6.2. 访视......10
7.6.3. 评估......10
7.6.3.1. ESSDAI......10
7.6.3.2. 干燥、疲乏、疼痛 VAS 评分......10
7.6.3.3. MFI-20 评分......11
7.6.3.4. 健康状况调查简表 SF-36......11
7.7. 生物标志物......11

7.8. 样品收集和管理......11
8. 研究药物信息......12
8.1. 研究药物的形状描述......12
8.2. 皮下给药......12
8.3. 包装......12
8.4. 标签......12
8.5. 准备、处理、储存......12
8.6. 药物问责......12
9. 安全性评价......13
9.1. 药物不良反应......13
9.1.1. 不良反应严重程度判断......13
9.1.2. 严重程度定义......14
9.1.3. 药物不良反应的处理措施及相关费用......14
10. 统计分析......14
10.1. 受试者概述......15
10.2. 样本量估计......15
10.3. 有效性分析......15
10.3.1. 主要终点分析......15
10.3.2. 次要终点......15
10.3.3. 其他分析......15
10.4. 安全性分析......16
11. 数据质量保证/质量控制......16
12. 数据管理......16
13. 道德规范......16
14. 总结和数据保存......16
附表：......17
1. 试验治疗期随访流程图......17
2. VAS 评分......18
3. 欧洲干燥综合征疾病活动度表（ESSDAI）......19
4. SF-36 生活质量表调查表......24
5. MFI-20 评分......27

1. 题目

低剂量白介素-2 治疗活动性干燥综合征：随机、双盲、安慰剂对照临床试验。

2. 项目摘要

低剂量重组人白介素-2（rhIL-2，商品名：欣吉尔）目前已被一系列开放性临床研究证明，在多种自身免疫性或自身炎症相关疾病治疗中有效，但是其在自身免疫性疾病中的真实疗效尚无严格的对照试验确证。本研究旨在通过随机、双盲、安慰剂对照临床试验来明确 rhIL-2 治疗干燥综合征的安全性和有效性，同时进一步探索该治疗对辅助性 T 细胞亚型的影响以明确其治疗的免疫机制。

3. 研究背景

原发性干燥综合征是一种以口干、眼干和多器官受累为特征的慢性自身免疫性疾病，包括周围神经病变、淋巴结病、肾小管酸中毒和间质性肺疾病。最近的研究表明，T 细胞和 B 细胞的失调在 PSS 的发生发展中起着至关重要的作用[1]。多种自身抗体的产生预示着 B 细胞耐受性的丧失。目前对 SS 的治疗是对症和经验性的。干燥表现是应用局部疗法来实现，如唾液替代和人工泪液治疗。全身损害的病人通常使用糖皮质激素联合免疫抑制剂治疗[2]。本研究旨在确认 rhIL-2 治疗干燥综合征的有效性和安全性。

4. 研究目的

本研究旨在确认rhIL-2治疗干燥综合征的有效性和安全性，同时进一步探索该治疗的免疫机制。

5. 研究设计

5.1. 干燥综合征

针对活动性干燥综合征在原治疗基础上调整药物治疗如下：

试验组（n=30）：原常规治疗方案基础上调整剂量+低剂量重组人白介素-2

对照组（n=30）：原常规治疗方案基础上调整剂量+安慰剂

5.2. 盲法的实施

本试验采用双盲的设计方法。重组人白介素-2 和安慰剂药物的包装外观、用法用量无差别。采用独立的第三方进行盲底的生成和现场盲法的实施。采用 SAS 9.3 统计软件，按 1:1 的比例产生随机编码表，即 SS01-60，入选的受试者将随机被分配入试验组或对照组。

5.3. 紧急揭盲

每个受试者都会有一个紧急揭盲信封，信封中记录了该受试者使用的具体药物，在紧急情况下需要知道其所用药物时才能打开。紧急信封由各中心负责的研究者或药剂师保管。在打开信封以前必须通知该中心主要研究者，得到其批准后

方可打开紧急信封。揭盲后该受试者将被终止试验，作为脱落病例处理。

5.4. 药物分配方法

符合纳入要求的受试者，根据其适应症，由药品管理员按入选时间的先后顺序，按药物号从小到大的顺序依次发放药物。

5.5. 合并用药规定

原治疗方案包括激素加或不加免疫抑制剂，醋酸泼尼松起始剂量≤7.5mg/d。

5.6. 研究人群

门诊或住院治疗的干燥综合征患者 60 例。

5.7. 入选和排除标准

5.7.1. 入选标准

1、男性或女性，且在筛选访视时年龄在 18-70 岁（含 18 及 65 岁），体重至少 35kg。

2、诊断为原发性干燥征（根据 2002 年 AECG 分类标准）[3]，且在筛查前确诊至少 3 个月。

3、疾病活动度（EESDAI）≥5 分；

4、RF、抗 SSA 或抗 SSB 抗体阳性。

5、诊断为原发性干燥综合征且病程超过 10 年的患者必须具有以下至少一个系统特征：

高球蛋白血症*（IgG>16.8g/L）或低补体 C3/C4*，或冷球蛋白血症*。

或自诊断为以下疾病以来的活动性或既往病史 （由干燥综合征引起）。

紫癜/皮肤血管炎，

淋巴结病，

非感染引起的持续性腮腺肿大，

外周神经病变（曾有神经传导试验记录），

HRCT 证实间质性肺疾病，

需要治疗的肾小管酸中毒，

干燥综合征引起的中枢神经系统疾病 （经 MRI 证实），

肌炎（CK 大于 2 倍正常上限）和肌电图或活检证实肌炎，

炎症性关节炎。

5、15 分钟内未刺激唾液流率大于 0ml。

6、症状性口干（VAS 评分≥50mm）。

7、症状性疲劳（VAS 评分≥50mm）。

8、症状性疼痛（VAS 评分≥50mm）

9、在接受首次治疗研究注射前应用糖皮质激素（≤7.5mg/d 强的松或相当剂量的其他激素）、DMARDs（如甲氨蝶呤、羟氯喹、硫唑嘌呤、吗替麦考酚酯、来氟米特、环孢素等）或匹罗卡品***必须稳定治疗 4 周，并且在整个研究中最好保持剂量不变。如果病人停用其中任何一种药物，那么在接受治疗研究前应停用至少 4 周。

10、参与试验时须给予病人书面告知同意书，并希望病人能够遵守研究随访计划和其他议定书的要求。

*在筛选后三个月内进行抗 Ro 抗体检测、IgG、RF、C3/C4 及冷球蛋白血症检测确定病人是否合格。如果超过 3 个月，应在本地检查时重复检查，以确认其是否合格。

VAS 评分范围为 0-100，100 对应最严重程度。

***在筛选、基线、12 周、24 周（研究结束）评估的 12 小时内毛果芸香碱或具有类似药理作用的药物禁止使用。

5.7.2. 排除标准

任何符合以下标准的受试者应被排除：

1、诊断为继发性干燥综合征。

2、任何 AECG 排除标准没有包括的其他情况（移植物抗宿主病，排除 pSS 的原发性淋巴瘤，结节病）

3、病情稳定（入组时 IgG ＜16.8 g/L）

4、之前使用利妥昔或其他单抗。

5、严重并发症：包括心力衰竭（≥ NYHA III 级），肾功能不全（肌酐清除率≤30 ml/min），肝功能不全（血清 ALT 或 AST 大于三倍正常上限，或总胆红素大于正常上限）

6、其他严重的、进展性的或不可控的血液学、胃肠、内分泌、肺、心、神经或脑疾病（包括脱髓鞘疾病，如多发性硬化）。

7、已知的过敏、高反应性或 IL-2 或其赋形剂不耐受。

8、患有严重感染（包括但不限于肝炎、肺炎、菌血症、肾盂肾炎、EB 病毒、结核感染），或因感染住院，或应用首剂治疗前 2 个月使用静脉抗生素治疗感染。

9、在首次使用研究制剂前 3 个月内胸部 X 线片显示有恶性肿瘤或当前活动性感染（包括结核病）的异常。

10、筛查前 6 个月内有非结核分枝杆菌感染或机会性感染（如巨细胞病毒、肺囊虫病、曲霉菌病）。

11、感染 HIV（HIV 抗体阳性血清学）或丙型肝炎（Hep C 抗体阳性血清学）。如果血清阳性，建议咨询在治疗艾滋病毒或丙型肝炎病毒感染方面有专长的医生。

12、感染乙型肝炎病毒。对于由于乙型肝炎病毒检测结果而不符合本研究条件的患者，建议咨询具有治疗乙型肝炎病毒感染专业知识的医生。

13、在过去 5 年内有任何已知的恶性肿瘤或有恶性肿瘤史（非黑色素瘤皮肤癌除外，在首个研究制剂使用前 3 个月内，无复发迹象的非黑色素瘤皮肤癌或手术治愈的宫颈肿瘤）。

14、有不受控制的精神或情绪障碍，包括在过去 3 年内有吸毒和酗酒史，这可能妨碍研究的顺利完成。

15、在首次注射研究剂前 3 个月内、在研究期间或在最后一次注射研究剂后 4 个月内，接受或预期接受任何活病毒或细菌疫苗注射。在筛查后 12 个月内接种了卡介苗。

16、怀孕、哺乳或有生育潜力的妇女（WCBP）在接受治疗期间和治疗结束后 12 个月内不愿使用经医学批准的避孕措施。

17、其伴侣有生育潜力但不愿在接受治疗期间和治疗结束后 12 个月内使用适当的经医学认可的避孕措施的男性。

5.8. 退出标准

受试者如果出现下述任一种事件，应退出研究：

1、受试者出现了时间上与注射研究药物相关的不良事件。

2、受试者撤回知情同意。

3、无法完成随访计划。

4、恶性肿瘤。

5、受试者在研究期间妊娠或计划妊娠。

6、使用了禁用的药物或治疗。

7、失访。

8、死亡。

如果受试者失访，研究工作人员必须尽力联系受试者并明确撤出的原因。必须记录随访采取的措施。当受试者在完成研究前撤出，撤出的原因应记录在 CRF 表和原始记录中。分配给受试者的研究药物不能分配给其他受试者。撤出研究的受试者不能被取代。撤出研究的受试者对于研究标本可做以下选择：保留已收集的样品并依照原始知情同意使用样品，或撤回对研究样品的知情同意，销毁样品，不再进行后续检测。

6. 试验药物信息

6.1. 药物

重组人白介素-2，安慰剂

6.2. 剂量和用法

受试者接受3个疗程的低剂量IL-2或安慰剂。具体方案为：1百万单位rhIL-2，皮下注射，隔日给药，连续给药14天，间歇14天后开始第二个疗程。所有患者前12周接受3个疗程的IL-2或安慰剂治疗，之后12周停药随访。

6.3. 伴随治疗

所有进入筛选前30天内的研究前用药必须在筛选时记录。研究过程中所有伴随治疗和任何变化都必须记录。应尽可能保持用药稳定到第24周。

6.3.1. 免疫调节剂

如果接受免疫调节剂，受试者应该从筛选到第24周剂量都稳定。免疫调节剂仅能在受试者出现不可接受的副作用时减量，而这可能影响受试者临床数据的解读。

6.3.2. 抗疟药

整个实验中可以使用稳定剂量的羟氯喹治疗。

6.3.3. 糖皮质激素治疗

不鼓励不必要的剂量变化，任何调整都要谨慎。医疗上必要时允许改变糖皮质激素治疗，但其程度和时机要谨慎考虑，因其可能影响研究结果。

初始治疗方案含糖皮质激素（醋酸泼尼松起始剂量≤7.5mg/d）伴或不伴免疫抑制剂。

如果受试者在激素减量过程中疾病活动度加重，可暂停进一步减量，和/或如果研究认为必要，其口服糖皮质激素剂量可临时提高。

6.3.4. 非甾体类抗炎药

应用NSAIDs（包括阿司匹林和选择性COX-2抑制剂）和其他止痛药的受试者应接受国家规定的常规剂量。NSAIDs处方和其他常规止痛药在应用首剂研究药物前2周至第24周不应调整，只有出现不可接受的不良反应时才能更改。在0-12周，治疗方案中不允许加用新的NSAIDs，在第12周后，允许微调NSAIDs药物，但推荐NSAIDs用药尽可能保持稳定，要记录任何显著改变。

6.3.5. 外用药

允许使用外用药，但不能含有禁止的药品。24周期间不允许使用环孢素的外用药膏或乳膏，但允许使用其眼药。允许使用外用眼药，比如用于治疗干眼症的玻璃酸钠眼药水和小牛血去蛋白提取物眼胶。在访视日以外可用低效价外用皮质激素，中高效价的外用糖皮质激素不允许使用。访视前72小时，未经评估不应在皮肤黏膜损害处使用外用药。

7. 研究过程

7.1. 筛选阶段

研究者在获取数据前必须并取得并核实知情同意书。筛选访视必须在随机访视（第 0 周）前 4 周内完成。另外，受试者在宣布筛选 SS 后必须已经接受研究随机分组。

为安全起见或为采集和分析特定样品的技术原因，可能会重复或额外采集样品。

研究者在随机分组前将进行所有用来明确受试者是否符合条件的筛选评估。所有受试者必须满足这些疾病的分类标准。

7.2. 双盲治疗阶段

7.2.1. 第 0 周/随机分组日

在第 0 周，符合条件的受试者将以 1:1 比例被随机双盲分配在人重组白介素-2 组或安慰剂组。

7.2.2. 安慰剂对照的治疗阶段

受试者以同样方式接受低剂量 rhIL-2 或安慰剂治疗。

受试者接受3个疗程的低剂量IL-2或安慰剂。每个疗程予1百万单位低剂量IL-2或安慰剂，皮下注射，隔日给药2周，之后2周停药随访。

7.3. 随访阶段

观察期为 12 周治疗及 12 周随访。收集患者临床表现、自身抗体和其他相关实验室检查，干燥综合征患者进行 ESSDAI 评分评估其病情缓解程度，在治疗前后监测药物不良反应。

7.4. 撤出研究的受试者

撤出研究的受试者不会被召回进行随访评估。

7.5. 有效性评估

有效性评估将基于主要和次要终点结果测评（详见 7.5.3）。描述所有有效性评估和终点、混合终点评估内容的完整列表详见附表 1 和 2。所有有效性评估应被研究者一致进行，以保持前后具有可比性。每次访视都会核实这些数据已采集并确保评估的一致性。

7.5.1. 终点

主要终点

主要终点是 24 周时，ESSDAI 评分改善≥3 分。

主要的次要终点

次要终点包括其他临床应答、安全性和免疫细胞亚群变化（包括 Treg、Tfh、Th17 和 CD8+T 细胞）。

7.6. 随访

7.6.1. 基线随访

（1）一般资料：包括姓名、性别、年龄、联系电话、病程。

（2）临床表现和体征：包括皮疹、发热、口腔溃疡、泡沫尿、疾病活动评分等。

（3）实验室检查：血常规、肝肾功能、ANA、抗 SSA、抗 SSB、RF、免疫球蛋白、ESR、CRP、T 细胞和 B 细胞亚群、IL-2、TGF-β 和其他细胞因子。

7.6.2. 访视

前 3 个月每 2 周访视 1 次（共 6 次），之后每个月访视 1 次。

（1）根据疾病活动评分（包括 ESSDAI 评分）观察临床表现是否缓解。

（2）监测血清学指标改变：每 4 周，血常规检查，肝肾功能，根据病情检测相关抗体如抗 SSA，抗 SSB，RF，免疫球蛋白，ESR，CRP 评估病情。

（3）流式细胞检测：第 0、6、12 和 24 周，流式细胞术检测 CD4+T 和 CD8+T 细胞亚群，评估治疗前后的细胞亚群变化率。

（4）细胞因子：在第 0，6，12 和第 24 周，通过 ELISA 检测 IL-2、IL-6、TGF-β 和其他细胞因子的水平。

7.6.3. 评估

描述所有有效性评估和终点、混合终点评估内容的完整列表详见附件 2。

7.6.3.1. ESSDAI

EULAR（欧洲抗风湿联盟）组织的国际 SS 专家合作组在 2010 年建立了 2 个广受认可的疾病活动性评分，即基于患者主观症状的（EULAR 干燥综合征患者报告指数）评分和基于患者客观系统性症状的 ESSDAI（干燥综合征疾病活动度指数）评分[4,5]。

筛选时（附件 6）进行 ESSDAI 评分，它是覆盖全身 12 个方面的疾病活动性指数，包括全身症状、淋巴结病变、腺体、皮肤黏膜、关节、肺部、肾脏、肌肉、周围神经、中枢神经、血液系统和生物学特征。每个方面分局活跃程度分为 3-4 级，每个方面的权重由多因素线性回归模型取得。只对跟疾病有关的症状评分，避免长期临床症状的影响。最终评分 0-123 分，0 分代表疾病无活动。

7.6.3.2. 干燥、疲乏、疼痛 VAS 评分

受试者将在一个可视化的模拟尺（VAS：0-100mm）上评估其过去 2 周的平均干燥、疲劳和疼痛程度。量尺的刻度从 0（代表"无症状"）到 100（代表"症状最重"）。

7.6.3.3. MFI-20 评分

问卷由 20 个项目组成，受试者必须在五点量表上标出其特定状态的程度，从"是"到"否"。正向和负向项目的计分相等时，可以相互抵消评分。分数越高，说明疲劳程度越高。

7.6.3.4. 健康状况调查简表 SF-36

RAND 健康状况调查简表（SF）-36 问卷是包含 36 项的自评多维量表。8 个健康维度包含功能评估（附件 3）[7]：

生理功能限制

日常角色活动限制

躯体疼痛

一般精神健康（精神压力和健康）

生命力（能量和疲劳）

因生理或精神问题造成的社会功能限制

因个人或情感问题造成的日常活动限制

一般健康感知

亚类评分从 0 到 100

评分由生理部分总分、精神部分总分、总分、亚类评分组成。评分越高，结果越好。14 岁以上适用，5-10 分钟完成。多数语言有翻译版本。量表已被广泛语言和文化核实。

SF-36 评价的概念不特别针对任何年龄、疾病或治疗组，可以比较不同疾病的相关负担和不同治疗的获益。任何亚类 3 分变化或组成部分 5 分变化是有临床意义的。

7.7. 生物标志物

血液、血清、尿液的收集、准备、储存和运输详见时间和事件表（表 1）和实验室手册。生物标志物包含但不限于 ESR、CRP 和其他炎症标志物、细胞表面标志物、自身抗体如抗 SSA、抗 SSB、RF、CD4+T 细胞和 CD19+B 细胞亚群，细胞因子（含 IL-2、IL-2、IL-6、IL-7、IL-10、IL-12p70、IL-15、IL-17A、IL-21、IFN-γ、TGF-β、IFN-α）和其他可能在 SS 发生发展中涉及的生物标志物种类。

7.8. 样品收集和管理

样品收集的实际日期和时间必须记录在实验室征用表中。

在时间和事件表（附表 2）中记录所有样品收集的时机和频率。收集、管理和运输样品的指导在实验室手册中，用于样品收集和管理。

8. 研究药物信息

8.1. 研究药物的形状描述

重组人白介素-2，其主要成分是重组人白细胞介素-2，来自重组大肠杆菌。辅剂是甘露醇，十二烷基硫酸钠，磷酸氢二钠，磷酸二氢钠，人白蛋白。安慰剂与IL-2具有相同的辅剂。

8.2. 皮下给药

受试者接受低剂量IL-2或安慰剂治疗3个疗程。具体方法为1百万单位，注射2ml，每隔一天皮下注射（rhIL-2 1×10^{6}IU，SC，Qod），共14天。停药14天后，开始另一个疗程。

8.3. 包装

研究用药将进行独立包装，以确保在整个供应链过程中对其进行适当管理。

8.4. 标签

研究药物标签将包含符合适用法规要求的信息。

8.5. 准备、处理、储存

所有研究试剂必须在2°C至8°C的受控温度下储存，不得冷冻，并避光。应避免剧烈摇晃产品。在给药之前，应目视检查产品的颗粒物质和变色。如果在溶液中观察到变色（除了浅黄色），可见的不透明颗粒或其他外来颗粒，则不应使用该产品。

在研究材料的制备和施用过程中必须使用无菌操作。在制备和给药过程中应避免暴露在直射阳光下。

8.6. 药物问责

研究者负责确保在整个研究过程中对在现场接收的所有研究药物进行清点和计算。对受试者施用的研究药物必须记录在药物责任表格中。研究现场人员不得将研究药物容器内的东西组合起来。

研究药物必须严格按照方案和容器标签进行处理，并且必须在适当的环境条件下存放在研究地点的限制访问区域或锁定的柜子中。未经使用的研究药物和受试者返回的研究药物必须在现场监测访问期间由赞助商的研究现场监测员进行验证。未使用的返回给保荐人的药物，或使用过的需进行销毁的药物，将被记录在药品退回表中。当研究地点是经过授权的销毁单位并且研究药物供应在现场销毁时，也必须在药物退回表上记录。

潜在危险的材料，如用过的输液袋，针头，注射器和小瓶应立即以安全的方

式处理，因药品责任的目的不能保留。

研究药物应在研究者或研究现场人员的合格成员或医院/诊所药剂师的监督下进行。研究药物仅供参与研究的受试者使用。返回的研究药物不得再次分发，即使是同一受试者。研究药物不得重新标记或重新分配以供其他受试者使用。研究人员一致同意，不得将研究药物从研究地点以外的任何地点分发，也不得存放在与赞助商商定的研究地点。

9. 安全性评价

9.1. 药物不良反应

药物不良反应包括了受试者应用 IL-2 治疗过程中发生的影响受试者健康的任何症状、综合征或疾病，也包括了实验室检查发现或其他诊断过程中发现的临床相关情况。

不良反应可能是：新的疾病；治疗状态症状或体征的恶化；伴随疾病的恶化等。

严重不良反应是指在用药过程中或观察期间任何时候出现的以下不良事件：包括：需住院；可能危害受试者或需要采取措施来预防的一种后果。

9.1.1. 不良反应严重程度判断

不良事件是指在临床研究受试对象在使用了研究（调查或非调查）产品中发生的不良的医疗事件。不良事件和治疗之间不一定存在因果关系。因此, 不良事件可能是任何不利的和意外的迹象（包括异常发现）、症状或在使用研究（调查或非调查）产品后出现的相关疾病，但不确切是否和研究（调查或非调查）产品存在因果关联。（国际协调会议[ICH]的定义）。

这包括和基线相比发生的任何新发疾病或严重程度或发生频率加重，或诊断过程中出现的异常结果，包括实验室检查异常。

注意：所有不良事件, 最后一次不良事件记录的时间）。

严重不良事件

根据 ICH 和欧盟的《药物使用警戒指南》，严重不良事件是指：在任何剂量下发生以下不良医疗事件：

死亡

危及生命 （事发时受试者有生命危险，并不包括假设事件更严重时将造成死亡）

需要住院治疗或延长住院时间。

持续或严重残疾-无行为能力

先天畸形/先天缺陷

通过研究药物发生的任何可疑的感染传播

在医学上是否重要

在迅速做出医学和科学的判断前，需考虑是否适合其他情况，如重要的医疗事件可能不会立即危及生命或导致死亡或住院，但可能危及受试者或需要干预来预防上诉任何一种不良事件的发生。以上均需要慎重考虑。

如果发生了严重和意外的不良事件，且有证据表明研究药物与该事件之间存在因果关系 （例如过敏反应导致的死亡），必须将该事件报告为严重和意外的可疑不良反应。

未列出的（意外的）不良事件/安全参考信息

当研究产品提供的安全信息和事件的性质或严重程度不一致时，则被视为未列出的不良事件。对于 rhIL-2，不良事件的发生机率主要取决于研究者是否将其列在调查人员手册中。

与药物使用相关的不良事件

根据定义，若不良事件和用药之间的关系可能、合理或非常有可能时，则认为两者相关。

9.1.2. 严重程度定义

严重程度分级主要根据以下定义：

轻度：受试者可以忍受，不影响治疗，不需要特别处理，对受试者无影响。

中度：受试者难以忍受，对受试者正常活动有直接影响。

重度：危及受试者生命，影响受试者功能或日常行为能力，需立即撤药或做紧急处理。

研究人员应用临床判断来评估和受试者不直接相关的事件严重性（如实验室异常）。

9.1.3. 药物不良反应的处理措施及相关费用

（1）低剂量注射用 rhIL-2 的常见不良反应在停药后可自行恢复。

（2）全身：当大剂量应用 IL-2 时，发热、寒战较常见，这与剂量相关；恶心、呕吐、流感样症状较少见。一旦出现，可继续观察，多可自行恢复。

（3）注射部位：红肿、硬结、疼痛，可继续观察，多可自行恢复，若持续无缓解，必要时减小剂量或停药。

（4）较大剂量时：毛细血管渗漏综合征、低血压等表现，需立即停药，必要时采用药物对症改善毛细血管通透性及低血压状态。

备注：本研究为小剂量IL-2治疗研究，临床应用和前期研究结果显示，尚未出现过大剂量IL-2引起的不良反应。

10. 统计分析

10.1. 受试者概述

接受过至少 1 次试验药物治疗的受试者均采集基线人口学及临床资料。有效性分析基于意向性治疗分析（mITT），也就是包括所有接受至少 1 次试验药物受试者。同时也进行符合方案集分析（PP），也就是完成治疗及随访受试者。安全性分析基于所有随机且接受至少 1 次治疗受试者。

10.2. 样本量估计

样本量估计基于每组受试者达到原始终点的比例。设定对照组 24 周达到原始终点比例为 30%，低剂量 IL-2 治疗组达到原始终点比例为 75%。各自需 28 例，可达到 90%以上的检验效能，显著性水平为 0.05。

10.3. 有效性分析

有效性分析基于意向性治疗分析（mITT）以及符合方案集分析（PP）。

10.3.1. 主要终点分析

干燥综合征原始终点为 24 周时 ESSDAI 评分改善≥3 分。

矫正基线的逻辑回归模型用于二分类变量的统计。连续变量采用协方差分析模型。纵向数据，对比不同随访时间，治疗有效性的分析，根据变量为连续变量或分类变量，分别采用重复测量混合模型以及广义评估方程。P 小于 0.05 为具有显著统计学意义。

10.3.2. 次要终点

干燥综合征次要终点为临床症状评价，干燥 VAS 评分、疼痛 VAS 评分、干燥 VAS 评分改善≥50mm 的比例；干燥 VAS 评分、疼痛 VAS 评分、干燥 VAS 评分和基线的变化；健康调查简表（SF-36），唾液腺流率以及 ESR），实验室指标包括免疫球蛋白 A，免疫球蛋白 B，抗 SSA 抗体、抗 SSB 抗体、泪膜破裂时间、泪河高度、Schirmer 试验、睑板腺数量、唾液腺超声、安全性评价以及 Treg 细胞、B10 细胞亚群。

连续变量分析采用协方差分析，其中以组别为固定因素，以基线资料作为协变量。如不满足平行性假设条件，应用非参数检验。矫正基线的逻辑回归模型用于二分类变量的统计。纵向数据，对比不同随访时间，治疗有效性的分析，根据变量为连续变量或分类变量，分别采用重复测量混合模型以及广义评估方程。P 小于 0.05 为具有显著统计学意义。

10.3.3. 其他分析

基线人口学以及临床疾病特征分析采用 t 检验、非参数检验比较连续变量，卡方检验比较分类变量。统计分析将采用 SPSS 17.0 统计分析软件进行计算。所有的统计检验均采用双侧检验，P 值小于或等于 0.05 将被认为所检验差别有统

计意义。可信区间采用 95%的可信度。

脱落分析：采用卡方检验计算脱落率。逐例描述各脱落受试者的人口学资料，主要指标和各时间点观察值。

10.4. 安全性分析

接受至少 1 次试验药物的受试者均纳入安全性分析。治疗过程中所有不良事件均纳入分析。针对每一个不良事件，至少发生 1 次的受试者所占比例进行统计。本研究分析感染的发生率以及感染类型，同时分析注射反应。导致退组的不良事件或者严重不良事件将格外重视。

11. 数据质量保证/质量控制

为确保数据的准确性和可靠性而采取的步骤包括选择合格的研究人员和适当的研究地点，在研究之前与研究人员和研究现场人员一起审议程序，由保荐人定期监测访问，以及直接传播临床实验室数据从中心实验室进入赞助商的数据库。将提供书面说明，用于样品的收集，处理，存储和运输。

在研究开始之前，将向研究现场人员提供CRF完成指南并进行审查。赞助商将在现场监测访问期间以及传输给赞助商之后审查CRF的准确性和完整性;任何差异将由调查员或指定人员酌情解决。在将数据上载到研究数据库之后，将验证它们与数据源的准确性和一致性。

12. 数据管理

研究人员应准确，及时地记录纸质和电子数据库版的病例数据。

13. 道德规范

经北京大学人民医院伦理委员会批准，所有受试者均获得知情同意书。

14. 总结和数据保存

保留原始记录。

根据数据库进行数据汇总，统计单位负责统计处理。知情同意书和CRF表将保留至研究结束后的5年。

附表：

1. 试验治疗期随访流程图

阶段	筛选期	治疗期（24 周）									
访视	V0	V1	V2	V3	V4	V5	V6	V7	V8	V9	V10
时间	-4-0 周	0 周	2 周±3 天	4 周±3 天	6 周±3 天	8 周±3 天	10 周±3 天	12 周±3 天	16 周±3 天	20 周±3 天	24 周±3 天
签署知情同意书[a]	√										
病史采集	√										
入选标准		√									
排除标准		√									
血常规[b]	√		√	√	√	√	√	√	√	√	√
尿常规[c]	√							√			√
肾小管功能[d]	√							√			√
尿 HCG[e]	√										
乙肝表面抗原	√										
丙肝抗体	√										
肝功能[f]	√							√			√
肾功能[g]	√							√			√
血脂[h]	√										
血糖	√										
电解质[i]	√							√			√
ESR	√	√	√	√	√	√	√	√	√	√	√
CRP	√	√	√	√	√	√	√	√	√	√	√
免疫球蛋白/补体	√		√	√	√	√	√	√	√	√	√
自身抗体[k]	√				√			√			√
细胞因子[l]		√			√			√			√
心电图	√										
眼科检查	√							√			√
肺功能[m]	√							√			√
唇腺活检[n]	√										
唾液腺彩超	√							√			√

**发放及回收药物**		√	√	√	√	√	√				
**不良事件情况记录**		√	√	√	√	√	√	√	√	√	√
**ESSDAI 评分**		√						√			√
**MFI-20 评分**		√						√			√
**VAS 评分**		√						√			√
**SF-36 评分**		√						√			√
**$CD4^{+}$T 细胞**		√			√			√			√
**CD8+T 细胞**		√			√			√			√
**日记卡发放与回收**		√									√

注：

a 筛选须在随机分组（基线 0 周）前的 4 周内进行。病史采集包括人口学资料、诊断、生命体征、是否初治、家族史、吸烟史、饮酒史、过敏史、主要病史、用药史、体格检查。

b 血常规：血红蛋白、红细胞比容、白细胞分类（嗜碱性粒细胞、嗜酸性粒细胞、淋巴细胞、单核细胞、中性粒细胞）、血小板。

c 尿常规：尿红细胞、白细胞、上皮细胞、结晶、颗粒管型、细菌。

d 肾小管功能：包括 β2 微球蛋白、N-乙酰氨基葡萄糖苷酶活性、视黄醇结合蛋白。

e 尿 HCG：尿妊娠试验。

f 肝功能：谷丙转氨酶、谷草转氨酶、白蛋白、总蛋白。

g 肾功能：肌酸激酶、尿素氮（BUN）、血肌酐、肾小球滤过率（eGFR）。

h 血脂：总胆固醇、甘油三酯、低密度脂蛋白胆固醇、高密度脂蛋白胆固醇。

i 电解质：钠、钾、钙、氯、二氧化碳结合力。

j 免疫球蛋白/补体：IgA，IgM，IgG，γ 球蛋白，C3，C4。

k 自身抗体：ANA、抗 SSA 抗体、抗 SSB 抗体、RF。

l 细胞因子：IL-2、TNF-α、IFN-γ、IL-6、IL-21、IL-17、TGF-β、sIL-2Rα 等。

2. VAS 评分

（1） 过去 2 周的干燥症状：包括口、眼、皮肤、鼻、气管及阴道。

无											有
	0	1	2	3	4	5	6	7	8	9	10

（2） 过去 2 周的疲乏程度。

无											有
	0	1	2	3	4	5	6	7	8	9	10

（3） 过去 2 周的肢体疼痛程度。

无	0	1	2	3	4	5	6	7	8	9	10	有

最终评分为干燥症状、肢体疼痛、疲乏三个积分的平均值，范围为 0-10 分。

3. 欧洲干燥综合征疾病活动度表（ESSDAI）

**全身症状[3]**<br>*注意除外与疾病无关的系统症状：如原发感染性发热，减肥所致体重下降。*		
**不活动**	无以下任何症状	☐
**低活动度**	轻微或间断发热（37.5°-38.5°C）或夜间盗汗<br>体重下降 5%到 10%	☐
**中活动度**	高热（>38.5°C）或夜间盗汗<br>体重下降大于 10%	☐

**淋巴结病变[4]**		
**不活动**	无以下任何症状	☐
**低活动度**	-任意部位淋巴结≥1cm 或腹股沟区淋巴结≥2cm	☐
**中活动度**	-任意部位淋巴结≥2cm 或腹股沟区淋巴结≥3cm<br>- 和/或脾脏肿大（临床可触及或影像学发现肿大）	☐
**高活动度**	-存在恶性 B 细胞增殖	☐

**腺体病变[2]***注意除外与疾病无关的腺体肿胀,如结石,感染*		
**不活动**	无腺体肿大	☐
**低活动度**	轻度腺体肿大:<br>- 腮腺肿大（≤ 3cm），<br>- 或局限性下颌下腺或泪腺肿大 [1]	☐
**中活动度**	重度腺体肿大:<br>- 腮腺肿大（>3cm ）<br>- 或广泛下颌下腺或泪腺肿大 [1]	☐

[1] 局限性和广泛性下颌下腺,泪腺肿大的区别由临床医生通过体检判断

**关节病变[2]**<br>*注意除外与疾病无关的关节受累,如骨关节炎*

**不活动**	目前没有活动性关节受累	☐
**低活动度**	手,腕,踝及足关节疼痛伴晨僵（>30 min）	☐
**中活动度**	28 个关节中有 1 到 5 个滑膜炎	☐
**高活动度**	28 个关节中≥ 6 个滑膜炎	☐

**粘膜病变[3]**<br>*注意：与损伤相关的粘膜病变归为不活动,排除与疾病活动度无关的粘膜病变*		
**不活动**	目前无活动性粘膜受累	☐
**低活动度**	多形红斑	☐
**中活动度**	局限性皮肤血管炎,包括荨麻疹性血管炎 [2],<br>或足及踝部紫癜,<br>或亚急性皮肤狼疮	☐
**高活动度**	弥漫性皮肤血管炎,包括荨麻疹性血管炎 [2],<br>或弥漫性紫癜<br>或与血管炎相关的溃疡	☐

[2] 局限性皮肤血管炎累及小于体表面积的 18%; 弥漫性皮肤血管炎累及大于体表面积 18% ;
体表面积（Body surface area,BSA）用九分区法定义（通常用来评估烧伤面积）, 具体如下:手掌（不包括手指）占1% BSA; 每个下肢占18% BSA;每个上肢占9% BSA;躯干（前面）占 18% BSA; 躯干 （后背） 占 18% BSA

**肺部病变[5]**<br>*注意：损作所致的肺部病变归为不活动性病变，除外与呼吸系统受累无关的病变（如吸烟）*		
**不活动**	目前无活动性肺部病变	☐
**低活动度**	持续咳嗽或支气管受累,但 X 线无异常表现<br>或放射学或 HRCT 诊断的肺间质病变 [3]:<br>• 无呼吸困难<br>• 肺功能正常	☐
**中活动度**	中度活动性肺部病变, 如 HRCT 诊断的肺间质病变:<br>• 活动后气短（NHYA II）<br>• 肺功能异常:<br>- $40\% \leq DL_{CO} < 70\%$和/或 $60\% \leq FVC < 80\%$	☐

**高活动度**	高度活动性肺间质病变,如 HRCT 诊断的肺间质病变:<br>• 休息时气短（NHYA III, IV）<br>• 或肺功能异常:<br>- DL_{CO}< 40% 和/或 FVC < 60%	☐

[3]HRCT 及放射学诊断的肺间质病变需要是 2 年内新诊断的.

DLCO=CO 扩散能力; FVC= 用力肺活量; HRCT= 高分辨率 CT; NHYA=组约心脏病协会分类

**肾脏疾病[5]**<br>*注意:与损伤相关的稳定的病变归为不活动性病变，并排除与肾脏疾病无关的病变*<br>如有肾活检结果,则首先按照组织特点确定疾病活动性.		
**不活动**	目前无活动性肾脏病变:<br>- 蛋白尿< 0.5g/d, 无血尿,无白细胞尿,无酸中毒.<br>- 或由于损伤所致的持续稳定的蛋白尿.	☐
**低活动度**	肾脏活动性病变, 包括:<br>• 肾小管酸中毒不伴肾功能损害（GFR[4]≥60ml/min）<br>• 肾小球病变:<br>- 蛋白尿 （0.5g/d 到 1 g/d）<br>- 无血尿及肾功能衰竭 （GFR4 ≥60ml/min）	☐
**中活动度**	中度肾脏活动性病变,如：<br>• 肾小管酸中毒伴肾功能衰竭（GFR[4]< 60 ml/min）<br>• 肾小球病变：<br>- 蛋白尿 1g/d 到 1.5g/d<br>- 无蛋白尿及肾功能衰竭 （GFR4 ≥ 60ml/min）<br>• 组织学证据：<br>- 外膜性肾小球肾炎<br>- 严重的间质淋巴细胞浸润	☐
**高活动度**	高度活动性肾脏病变,如:<br>• 肾小球病变：<br>- 蛋白尿大于 1.5 g/d<br>- 或血尿<br>- 或肾功能衰竭（GFR[4]< 60 ml/min）<br>• 或组织学证据：<br>- 增生性肾小球肾炎<br>- 冷球蛋白相关的肾功能病变	☐

[4] MDRD 公式法估算肾小球滤过率（GFR）

**肌肉病变[6]**<br>*注意排除与疾病无关的肌肉受累,如糖皮质激素引起的肌无力*

**不活动**	目前无活动性肌肉病变	☐
**低活动度**	肌电图或肌肉活检诊断的活动性肌炎:<br>- 无肌无力<br>- 肌酸肌酶 （N < CK ≤ 2N）	☐
**中活动度**	经 EMG 或肌肉活检证实的中度活动性肌炎：<br>- 肌无力（肌力大于等于 4 级）<br>- 或肌酸肌酶升高（2N < CK ≤ 4N）	☐
**高活动度**	经 EMG 或肌肉活检证实的高度活动性肌炎:<br>- 肌无力（肌力≤ 3/5）<br>- 或肌酸肌酶升高 （> 4N）	☐

EMG= electromyogram, 肌电图;

**外周神经病变[5]**<br>*注意：与损伤相关的稳定的病变归为不活动性病变，并排除与疾病无关的 PNS 病变*		
**不活动**	目前无活动性外周神经病变	☐
**低活动度**	活动性外周神经病变,如:<br>- - NCS 证实的单纯感觉轴索神经病变<br>- - 三叉神经（V）痛	☐
**中活动度**	NCS 证实的中度活动性外周神经病变，如：<br>- 轴索感觉-运动神经病变伴运动功能缺失≤4/5<br>- 单纯感觉神经病变伴冷球蛋白血症型血管炎<br>- 神经节病变 [5] 所致的轻/中度运动失调<br>- 炎症性脱髓鞘性多发神经病变 [6]（CIDP）伴轻度功能障碍（运动功能缺失最多为 4/5 或轻度运动失调）<br>或脑神经的外周病变 （三叉神经（V）痛除外）	☐
**高活动度**	NCS 证实的高度活动性外周神经病变，如：<br>- 轴索感觉-运动神经病变伴运动功能缺失≤ 3/5<br>- 血管炎导致的外周神经病变（复合性单神经炎等）<br>- 神经节病变导致的重度共济性运动失调 [5]<br>- 炎症性脱髓鞘性多发神经病变 [6] （CIDP）伴重度功能障碍：运动功能缺失≤ 3/5 或重度运动失调	☐

NCS: 神经传导检查.

[5] 单纯感觉障碍伴运动失调和弥散性功能障碍或感觉缺失

[6]多神经根病变存在以下 4 种临床症状（四肢感觉运动缺失，近端运动障碍, 广义的无反射，上肢感觉异常和/或相关的颅神经病变），蛋白水平升高和/或NCS异常（远端运动电位潜伏期延长, 神经传导速度下降,F波潜伏期延长, 传导阻滞和/或时间上的离散）

**中枢神经病变[5]**<br>*注意：与损伤相关的稳定的病变归为不活动性病变，并排除与疾病无关的 CNS 病变*		
**不活动**	目前无活动性 CNS 病变	☐
**中活动度**	中度活动性 CNS 病变，如：<br>-颅神经的中枢病变<br>-视神经炎<br>-多发性硬化样综合征出现单纯感觉障碍或知觉障碍	☐
**高活动度**	高度活动性 CNS 病变，如：<br>- 脑血管炎伴意外或短暂失血发作<br>- 失神小发作 s<br>- 横贯性脊髓炎<br>- 淋巴细胞性脑膜炎<br>-多发性硬化样综合征出现运动功能缺失	☐

**血液系统[2]**<br>*注意：*<br>*- 仅包括自身免疫性贫血[7],血小板减少[8] 和中性粒细胞减少症[9]*<br>*- 排除与疾病无关的血细胞减少症 （ 如维生素或铁缺乏引起的血细胞减少或药物性血细胞减少,如环磷酰胺引起的淋巴细胞减少）*		
**不活动**	无自身免疫性血细胞减少	☐
**低活动度**	自身免疫性血细胞减少：<br>-中性粒细胞减少症 （1000 <中性粒细胞< 1500/mm3）<br>- 或贫血 （10 <Hb < 12g/dl）<br>-或血小板减少症（ 100,000 <血小板< 150,000/mm3）<br>或淋巴细胞减少症（500<淋巴细胞<1000/mm3）	☐
**中活动度**	自身免疫性血小板减少：<br>- 中性粒细胞减少（500 ≤ 中性粒细胞 ≤ 1000/mm3），<br>-或贫血 （8 ≤ Hb ≤ 10g/dl）<br>-或血小板减少症（50,000 ≤ 血小板 ≤ 100,000/mm3）<br>或淋巴细胞减少症（≤500/mm3）	☐
**高活动度**	自身免疫性血细胞减少：<br>-中性粒细胞减少症 （中性粒细胞< 500/mm3），<br>- 或贫血（Hb < 8 g/dl）<br>- 或血小板减少症（血小板 < 50,000/mm3）	☐

[7] Coombs 试验阳性伴网织红细胞增多的贫血

8 无其它原因的循环血小板减少，或抗血小板自身抗体阳性和/或骨髓活检有巨噬细胞存在和/或相关的自身免疫性贫血

[9] 无其它原因的中性粒细胞减少症。

生物学特征[1]		
不活动	无以下任何生物学特征	☐
低活动度	-补体正常<br>-或低补体血症 （C4 ，C3 or CH50 低）<br>- 或高丙球蛋白血症或 16 g/L <IgG<20g/L	☐
中活动度	- 冷球蛋白血症<br>- 或高丙球蛋白血症或 IgG> 20g/L<br>- 近期[10]发生的低丙球蛋白血症或 IgG 减少（<5g/L）	☐

[10] 最近 6 个月

计分方法:

每个系统的得分按该系统活动度来评，在其后对应的方括号内。
活动水平按如下方法评分:
不活动= 0
低活动度 = 1
中等活动度 = 2
高活动度 = 3
总分是各个系统病变得分之和。

4. SF-36 生活质量表调查表

1. 总体来讲，您的健康状况是：
①非常好 ②很好 ③好 ④一般 ⑤差
2. 跟 1 年以前比，您觉得自己的健康状况是：
①比 1 年前好多了 ②比 1 年前好一些 ③跟 1 年前差不多
④比 1 年前差一些 ⑤比 1 年前差多了
（权重或得分依次为 1、2、3、4 和 5）

[健康和日常活动]

3. 以下这些问题都和日常活动有关。请您想一想，您的健康状况是否限制了这些活动?如果有限制，程度如何？
（1）重体力活动，如跑步举重、参加剧烈运动等：
①限制很大 ②有些限制 ③毫无限制
（权重或得分依次为 1、2、3，下同）
（2）适度的活动，如移动一张桌子、扫地、打太极拳、做简单体操等：
①限制很大 ②有些限制 ③毫无限制
（3）手提日用品，如买菜、购物等：
①限制很大 ②有些限制 ③毫无限制
（4）上几层楼梯：
①限制很大 ②有些限制 ③毫无限制
（5）上一层楼梯：
①限制很大 ②有些限制 ③毫无限制

（6）弯腰、屈膝、下蹲：
①限制很大 ②有些限制 ③毫无限制
（7）步行 1500 米以上的路程：
①限制很大 ②有些限制 ③毫无限制
（8）步行 1000 米的路程：
①限制很大 ②有些限制 ③毫无限制
（9）步行 100 米的路程：
①限制很大 ②有些限制 ③毫无限制
（10）自己洗澡、穿衣：
①限制很大 ②有些限制 ③毫无限制

4. 在过去 4 个星期里，您的工作和日常活动有无因为身体健康的原因而出现以下这些问题？
（1）减少了工作或其他活动时间：
①是 ②不是
（权重或得分依次为 1、2，下同）
（2）本来想要做的事情只能完成一部分：
①是 ②不是
（3）想要干的工作或活动种类受到限制：
①是 ②不是
（4）完成工作或其他活动困难增多（比如需要额外的努力）：
①是 ②不是

5. 在过去 4 个星期里，您的工作和日常活动有无因为情绪的原因（如压抑或忧虑）而出现以下这些问题？
（1）减少了工作或活动时间：
①是 ②不是
（权重或得分依次为 1、2，下同）
（2）本来想要做的事情只能完成一部分：
①是 ②不是
（3）做事情不如平时仔细：
①是 ②不是

6. 在过去 4 个星期里，您的健康或情绪不好在多大程度上影响了您与家人、朋友、邻居或集体的正常社会交往？
①完全没有影响 ②有一点影响 ③中等影响 ④影响很大 ⑤影响非常大
（权重或得分依次为 5、4、3、2、1）

7. 在过去 4 个星期里，您有身体疼痛吗？
①完全没有疼痛 ②有一点疼痛 ③中等疼痛 ④严重疼痛 ⑤很严重疼痛
（权重或得分依次为 6、5.4、4.2、3.1、2.2、1）

8. 在过去 4 个星期里，您的身体疼痛影响了您的工作和家务吗？
①完全没有影响 ②有一点影响 ③中等影响 ④影响很大 ⑤影响非常大
（如果 7 无 8 无，权重或得分依次为 6、4.75、3.5、2.25、1.0；如果为 7 有 8 无，则为 5、4、3、2、1）

[您的感觉]

9. 以下这些问题是关于过去 1 个月里您自己的感觉，对每一条问题所说的事情，您的情况是什么样的？

（1）您觉得生活充实：
①所有的时间 ②大部分时间 ③比较多时间 ④一部分时间
⑤小部分时间 ⑥没有这种感觉
（权重或得分依次为 6、5、4、3、2、1）
（2）您是一个敏感的人：
①所有的时间 ②大部分时间 ③比较多时间 ④一部分时间
⑤小部分时间 ⑥没有这种感觉
（权重或得分依次为 1、2、3、4、5、6）
（3）您的情绪非常不好，什么事都不能使您高兴起来：
①所有的时间 ②大部分时间 ③比较多时间 ④一部分时间
⑤小部分时间 ⑥没有这种感觉
（权重或得分依次为 1、2、3、4、5、6）
（4）您的心理很平静：
①所有的时间 ②大部分时间 ③比较多时间 ④一部分时间
⑤小部分时间 ⑥没有这种感觉
（权重或得分依次为 6、5、4、3、2、1）
（5）您做事精力充沛：
①所有的时间 ②大部分时间 ③比较多时间 ④一部分时间
⑤小部分时间 ⑥没有这种感觉
（权重或得分依次为 6、5、4、3、2、1）
（6）您的情绪低落：
①所有的时间 ②大部分时间 ③比较多时间 ④一部分时间
⑤小部分时间 ⑥没有这种感觉
（权重或得分依次为 1、2、3、4、5、6）
（7）您觉得筋疲力尽：
①所有的时间 ②大部分时间 ③比较多时间 ④一部分时间
⑤小部分时间 ⑥没有这种感觉
（权重或得分依次为 1、2、3、4、5、6）
（8）您是个快乐的人：
①所有的时间 ②大部分时间 ③比较多时间 ④一部分时间
⑤小部分时间 ⑥没有这种感觉
（权重或得分依次为 6、5、4、3、2、1）
（9）您感觉厌烦：
①所有的时间 ②大部分时间 ③比较多时间 ④一部分时间
⑤小部分时间 ⑥没有这种感觉
（权重或得分依次为 1、2、3、4、5、6）
10. 不健康影响了您的社会活动（如走亲访友）：
①所有的时间 ②大部分时间 ③比较多时间 ④一部分时间
⑤小部分时间 ⑥没有这种感觉
（权重或得分依次为 1、2、3、4、5、6）

[总体健康情况]

11. 请看下列每一条问题，哪一种答案最符合您的情况？
（1）我好象比别人容易生病：
①绝对正确 ②大部分正确 ③不能肯定 ④大部分错误 ⑤绝对错误`

（权重或得分依次为 1、2、3、4、5）
（2）我跟周围人一样健康：
①绝对正确 ②大部分正确 ③不能肯定 ④大部分错误 ⑤绝对错误
（权重或得分依次为 5、4、3、2、1）
（3）我认为我的健康状况在变坏：
①绝对正确 ②大部分正确 ③不能肯定 ④大部分错误 ⑤绝对错误
（权重或得分依次为 1、2、3、4、5）
（4）我的健康状况非常好：
①绝对正确 ②大部分正确 ③不能肯定 ④大部分错误 ⑤绝对错误
（权重或得分依次为 5、4、3、2、1）
SF-36 生活质量评分统计

题号	计分	题号	计分	题号	计分	题号	计分
1		3-9		7		9-9	
2		3-10		8		10	
3-1		4-1		9-1		11-1	
3-2		4-2		9-2		11-2	
3-3		4-3		9-3		11-3	
3-4		4-4		9-4		11-4	
3-5		5-1		9-5			
3-6		5-2		9-6			
3-7		5-3		9-7			
3-8		6		9-8		合计	

5. MFI-20 评分

多维疲劳量表（multidimensional fatigue inventory-20,MFI-20）

No.	Item	是	1	2	3	4	5	否
1	我感觉良好		□	□	□	□	□	
2	我感觉只能做一点体力活动		□	□	□	□	□	
3	我感觉很有活力		□	□	□	□	□	
4	我愿做各种令我开心的事		□	□	□	□	□	
5	我觉得疲惫		□	□	□	□	□	
6	我觉得我一天干太多的活		□	□	□	□	□	
7	我能专心做事		□	□	□	□	□	
8	在体力上我能做很多事		□	□	□	□	□	
9	我害怕必须做事		□	□	□	□	□	
10	我一天只能做很少的事		□	□	□	□	□	
11	我能很好地集中精力		□	□	□	□	□	
12	我一直在休息		□	□	□	□	□	
13	我要很努力才能集中精神		□	□	□	□	□	

14	我要很努力才能应对糟糕的处境	□	□	□	□	□
15	我有很多工作计划	□	□	□	□	□
16	我容易觉得疲劳	□	□	□	□	□
17	我几乎没做任何事	□	□	□	□	□
18	我不想做任何事	□	□	□	□	□
19	我容易走神	□	□	□	□	□
20	我感觉体力状况很好	□	□	□	□	□

--请在每个条目后选择该表述与您相符的程度，越符合越偏向 1 侧，越不符合越偏向 5 侧:

--表述疲劳的条目 2、5、9、10、13、14、 16、17、18、19，为正向计分；

--不表述疲劳的条目 1、3、4、6、7、8、 11、12、15、20，为反向计分；

--其分数越高,说明疲劳症状越严重

References:

1. Reksten TR, Jonsson MV, Szyszko EA, Brun JG, Jonsson R, Brokstad KA. Cytokine and autoantibody profiling related to histopathological features in primary Sjogren's syndrome. Rheumatology (Oxford). 2009; **48**(9): 1102-6.
2. Ramos-Casals M, Brito-Zeron P, Siso-Almirall A, Bosch X, Tzioufas AG. Topical and systemic medications for the treatment of primary Sjogren's syndrome. Nature reviews Rheumatology. 2012; **8**(7): 399-411.
3. Vitali C, Bombardieri S, Jonsson R, Moutsopoulos HM, Alexander EL, Carsons SE, et al. Classification criteria for Sjogren's syndrome: a revised version of the European criteria proposed by the American-European Consensus Group. Annals of the rheumatic diseases. 2002; **61**(6): 554-8.
4. Seror R, Ravaud P, Mariette X, Bootsma H, Theander E, Hansen A, et al. EULAR Sjogren's Syndrome Patient Reported Index (ESSPRI): development of a consensus patient index for primary Sjogren's syndrome. Annals of the rheumatic diseases. 2011; **70**(6): 968-72.
5. Seror R, Ravaud P, Bowman SJ, Baron G, Tzioufas A, Theander E, et al. EULAR Sjogren's syndrome disease activity index: development of a consensus systemic disease activity index for primary Sjogren's syndrome. Annals of the rheumatic diseases. 2010; **69**(6): 1103-9.
6. Smets EM, Garssen B, Bonke B, De Haes JC. The Multidimensional Fatigue Inventory (MFI) psychometric qualities of an instrument to assess fatigue. Journal of psychosomatic research. 1995; **39**(3): 315-25.
7. Ware JE, Jr., Sherbourne CD. The MOS 36-item short-form health survey (SF-36). I. Conceptual framework and item selection. Medical care. 1992; **30**(6): 473-83.

研究方案

低剂量白介素-2治疗活动性干燥综合征：随机、双盲、安慰剂对照临床试验

研究单位：北京大学人民医院

二O一五年十二月二十一日

目录

目录........2
1. 题目........4
2. 项目摘要........4
3. 研究背景........4
4. 研究目的........4
5. 研究设计........4
5.1. 干燥综合征........4
5.2. 盲法的实施........4
5.3. 紧急揭盲........4
5.4. 药物分配方法........5
5.5. 合并用药规定........5
5.6. 研究人群........5
5.7. 入选和排除标准........5
5.7.1. 入选标准........5
5.7.2. 排除标准........6
5.8. 退出标准........7
6. 试验药物信息........7
6.1. 药物........7
6.2. 剂量和用法........7
6.3. 伴随治疗........8
6.3.1. 免疫调节剂........8
6.3.2. 抗疟药........8
6.3.3. 糖皮质激素治疗........8
6.3.4. 非甾体类抗炎药........8
6.3.5. 外用药........8
7. 研究过程........8
7.1. 筛选阶段........8
7.2. 双盲治疗阶段........9
7.2.1. 第 0 周/随机分组日........9
7.2.2. 安慰剂对照的治疗阶段........9
7.3. 随访阶段........9
7.4. 撤出研究的受试者........9
7.5. 有效性评估........9
7.5.1. 终点........9
7.6. 随访........10
7.6.1. 基线随访........10
7.6.2. 访视........10
7.6.3. 评估........10
7.6.3.1. ESSDAI........10
7.6.3.2. 干燥、疲乏、疼痛 VAS 评分........10
7.6.3.3. MFI-20 评分........11
7.6.3.4. 健康状况调查简表 SF-36........11
7.7. 生物标志物........11

7.8. 样品收集和管理 11
8. 研究药物信息 12
8.1. 研究药物的形状描述 12
8.2. 皮下给药 12
8.3. 包装 12
8.4. 标签 12
8.5. 准备、处理、储存 12
8.6. 药物问责 12
9. 安全性评价 13
9.1. 药物不良反应 13
9.1.1. 不良反应严重程度判断 13
9.1.2. 严重程度定义 14
9.1.3. 药物不良反应的处理措施及相关费用 14
10. 统计分析 14
10.1. 受试者概述 15
10.2. 样本量估计 15
10.3. 有效性分析 15
10.3.1. 主要终点分析 15
10.3.2. 次要终点 15
10.3.3. 其他分析 15
10.4. 安全性分析 16
11. 数据质量保证/质量控制 16
12. 数据管理 16
13. 道德规范 16
14. 总结和数据保存 16
附表： 17
1. 试验治疗期随访流程图 17
2. VAS 评分 18
3. 欧洲干燥综合征疾病活动度表（**ESSDAI**） 19
4. SF-36 生活质量表调查表 24
5. MFI-20 评分 27

1. 题目

低剂量白介素-2 治疗活动性干燥综合征：随机、双盲、安慰剂对照临床试验。

2. 项目摘要

低剂量重组人白介素-2（rhIL-2，商品名：欣吉尔）目前已被一系列开放性临床研究证明，在多种自身免疫性或自身炎症相关疾病治疗中有效，但是其在自身免疫性疾病中的真实疗效尚无严格的对照试验确证。本研究旨在通过随机、双盲、安慰剂对照临床试验来明确 rhIL-2 治疗干燥综合征的安全性和有效性，同时进一步探索该治疗对辅助性 T 细胞亚型的影响以明确其治疗的免疫机制。

3. 研究背景

原发性干燥综合征是一种以口干、眼干和多器官受累为特征的慢性自身免疫性疾病，包括周围神经病变、淋巴结病、肾小管酸中毒和间质性肺疾病。最近的研究表明，T 细胞和 B 细胞的失调在 PSS 的发生发展中起着至关重要的作用[1]。多种自身抗体的产生预示着 B 细胞耐受性的丧失。目前对 SS 的治疗是对症和经验性的。干燥表现是应用局部疗法来实现，如唾液替代和人工泪液治疗。全身损害的病人通常使用糖皮质激素联合免疫抑制剂治疗[2]。本研究旨在确认 rhIL-2 治疗干燥综合征的有效性和安全性。

4. 研究目的

本研究旨在确认rhIL-2治疗干燥综合征的有效性和安全性，同时进一步探索该治疗的免疫机制。

5. 研究设计

5.1. 干燥综合征

针对活动性干燥综合征在原治疗基础上调整药物治疗如下：

试验组（n=30）：原常规治疗方案基础上调整剂量+低剂量重组人白介素-2

对照组（n=30）：原常规治疗方案基础上调整剂量+安慰剂

5.2. 盲法的实施

本试验采用双盲的设计方法。重组人白介素-2 和安慰剂药物的包装外观、用法用量无差别。采用独立的第三方进行盲底的生成和现场盲法的实施。采用 SAS 9.3 统计软件，按 1:1 的比例产生随机编码表，即 SS01-60，入选的受试者将随机被分配入试验组或对照组。

5.3. 紧急揭盲

每个受试者都会有一个紧急揭盲信封，信封中记录该受试者使用的具体药物，在紧急情况下需要知道其所用药物时才能打开。紧急信封由各中心负责的研究者或药剂师保管。在打开信封以前必须通知该中心主要研究者，得到其批准后方可

打开紧急信封。揭盲后该受试者将被终止试验，作为脱落病例处理。

5.4. 药物分配方法

符合纳入要求的受试者，根据其适应症，由药品管理员按入选时间的先后顺序，按药物号从小到大的顺序依次发放药物。

5.5. 合并用药规定

原治疗方案包括激素加或不加免疫抑制剂，醋酸泼尼松起始剂量≤7.5mg/d。

5.6. 研究人群

门诊或住院治疗的干燥综合征患者 60 例。

5.7. 入选和排除标准

5.7.1. 入选标准

1、男性或女性，且在筛选访视时年龄在 18-70 岁（含 18 及 65 岁），体重至少 35kg。

2、诊断为原发性干燥征（根据 2002 年 AECG 分类标准）[3]，且在筛查前确诊至少 3 个月。

3、疾病活动度（EESDAI）≥5 分；

4、RF、抗 SSA 或抗 SSB 抗体阳性。

5、诊断为原发性干燥综合征且病程超过 10 年的患者必须具有以下至少一个系统特征：

高球蛋白血症*（IgG>16.8g/L）或低补体 C3/C4*，或冷球蛋白血症*。

或自诊断为以下疾病以来的活动性或既往病史 （由干燥综合征引起）。

紫癜/皮肤血管炎，

淋巴结病，

非感染引起的持续性腮腺肿大，

外周神经病变（曾有神经传导试验记录），

HRCT 证实间质性肺疾病，

需要治疗的肾小管酸中毒，

干燥综合征引起的中枢神经系统疾病 （经 MRI 证实），

肌炎（CK 大于 2 倍正常上限）和肌电图或活检证实肌炎，

炎症性关节炎。

5、15 分钟内未刺激唾液流率大于 0ml。

6、症状性口干（VAS 评分≥50mm）。

7、症状性疲劳（VAS 评分≥50mm）。

8、症状性疼痛（VAS 评分≥50mm）

9、在接受首次治疗研究注射前应用糖皮质激素（≤7.5mg/d 强的松或相当剂量的其他激素）、DMARDs（如甲氨蝶呤、羟氯喹、硫唑嘌呤、吗替麦考酚酯、来氟米特、环孢素等）或匹罗卡品***必须稳定治疗 4 周，并且在整个研究中最好保持剂量不变。如果病人停用其中任何一种药物，那么在接受治疗研究前应停用至少 4 周。

10、参与试验时须给予病人书面告知同意书，并希望病人能够遵守研究随访计划和其他议定书的要求。

*在筛选后三个月内进行抗 Ro 抗体检测、IgG、RF、C3/C4 及冷球蛋白血症检测确定病人是否合格。如果超过 3 个月，应在本地检查时重复检查，以确认其是否合格。

VAS 评分范围为 0-100，100 对应最严重程度。

***在筛选、基线、12 周、24 周（研究结束）评估的 12 小时内毛果芸香碱或具有类似药理作用的药物禁止使用。

5.7.2. 排除标准

任何符合以下标准的受试者应被排除：

1、诊断为继发性干燥综合征。

2、任何 AECG 排除标准没有包括的其他情况（移植物抗宿主病，排除 pSS 的原发性淋巴瘤，结节病）

3、病情稳定（入组时 IgG ＜16.8 g/L）

4、之前使用利妥昔或其他单抗。

5、严重并发症：包括心力衰竭（≥ NYHA III 级），肾功能不全（肌酐清除率≤30 ml/min），肝功能不全（血清 ALT 或 AST 大于三倍正常上限，或总胆红素大于正常上限）

6、其他严重的、进展性的或不可控的血液学、胃肠、内分泌、肺、心、神经或脑疾病（包括脱髓鞘疾病，如多发性硬化）。

7、已知的过敏、高反应性或 IL-2 或其赋形剂不耐受。

8、患有严重感染（包括但不限于肝炎、肺炎、菌血症、肾盂肾炎、EB 病毒、结核感染），或因感染住院，或应用首剂治疗前 2 个月使用静脉抗生素治疗感染。

9、在首次使用研究制剂前 3 个月内胸部 X 线片显示有恶性肿瘤或当前活动性感染（包括结核病）的异常。

10、筛查前 6 个月内有非结核分枝杆菌感染或机会性感染（如巨细胞病毒、肺囊虫病、曲霉菌病）。

11、感染 HIV（HIV 抗体阳性血清学）或丙型肝炎（Hep C 抗体阳性血清学）。如果血清阳性，建议咨询在治疗艾滋病毒或丙型肝炎病毒感染方面有专长的医生。

12、感染乙型肝炎病毒。对于由于乙型肝炎病毒检测结果而不符合本研究条件的患者，建议咨询具有治疗乙型肝炎病毒感染专业知识的医生。

13、在过去 5 年内有任何已知的恶性肿瘤或有恶性肿瘤史（非黑色素瘤皮肤癌除外，在首个研究制剂使用前 3 个月内，无复发迹象的非黑色素瘤皮肤癌或手术治愈的宫颈肿瘤）。

14、有不受控制的精神或情绪障碍，包括在过去 3 年内有吸毒和酗酒史，这可能妨碍研究的顺利完成。

15、在首次注射研究剂前 3 个月内、在研究期间或在最后一次注射研究剂后 4 个月内，接受或预期接受任何活病毒或细菌疫苗注射。在筛查后 12 个月内接种了卡介苗。

16、怀孕、哺乳或有生育潜力的妇女（WCBP）在接受治疗期间和治疗结束后 12 个月内不愿使用经医学批准的避孕措施。

17、其伴侣有生育潜力但不愿在接受治疗期间和治疗结束后 12 个月内使用适当的经医学认可的避孕措施的男性。

5.8. 退出标准

受试者如果出现下述任一种事件，应退出研究：

1、受试者出现了时间上与注射研究药物相关的不良事件。

2、受试者撤回知情同意。

3、无法完成随访计划。

4、恶性肿瘤。

5、受试者在研究期间妊娠或计划妊娠。

6、使用了禁用的药物或治疗。

7、失访。

8、死亡。

如果受试者失访，研究工作人员必须尽力联系受试者并明确撤出的原因。必须记录随访采取的措施。当受试者在完成研究前撤出，撤出的原因应记录在 CRF 表和原始记录中。分配给受试者的研究药物不能分配给其他受试者。撤出研究的受试者不能被取代。撤出研究的受试者对于研究标本可做以下选择：保留已收集的样品并依照原始知情同意使用样品，或撤回对研究样品的知情同意，销毁样品，不再进行后续检测。

6. 试验药物信息

6.1. 药物

重组人白介素-2，安慰剂

6.2. 剂量和用法

受试者接受3个疗程的低剂量IL-2或安慰剂。具体方案为：1百万单位rhIL-2，皮下注射，隔日给药，连续给药14天，间歇14天后开始第二个疗程。所有患者前12周接受3个疗程的IL-2或安慰剂治疗，之后12周停药随访。

6.3. 伴随治疗

所有进入筛选前30天内的研究前用药必须在筛选时记录。研究过程中所有伴随治疗和任何变化都必须记录。应尽可能保持用药稳定到第24周。

6.3.1. 免疫调节剂

如果接受免疫调节剂，受试者应该从筛选到第24周剂量都稳定。免疫调节剂仅能在受试者出现不可接受的副作用时减量，而这可能影响受试者临床数据的解读。

6.3.2. 抗疟药

整个实验中可以使用稳定剂量的羟氯喹治疗。

6.3.3. 糖皮质激素治疗

不鼓励不必要的剂量变化，任何调整都要谨慎。医疗上必要时允许改变糖皮质激素治疗，但其程度和时机要谨慎考虑，因其可能影响研究结果。

初始治疗方案含糖皮质激素（醋酸泼尼松起始剂量≤7.5mg/d）伴或不伴免疫抑制剂。

如果受试者在激素减量过程中疾病活动度加重，可暂停进一步减量，和/或如果研究认为必要，其口服糖皮质激素剂量可临时提高。

6.3.4. 非甾体类抗炎药

应用NSAIDs（包括阿司匹林和选择性COX-2抑制剂）和其他止痛药的受试者应接受国家规定的常规剂量。NSAIDs处方和其他常规止痛药在应用首剂研究药物前2周至第24周不应调整，只有出现不可接受的不良反应时才能更改。在0-12周，治疗方案中不允许加用新的NSAIDs，在第12周后，允许微调NSAIDs药物，但推荐NSAIDs用药尽可能保持稳定，要记录任何显著改变。

6.3.5. 外用药

允许使用外用药，但不能含有禁止的药品。24周期间不允许使用环孢素的外用药膏或乳膏，但允许使用其眼药。允许使用外用眼药，比如用于治疗干眼症的玻璃酸钠眼药水和小牛血去蛋白提取物眼胶。在访视日以外可用低效价外用皮质激素，中高效价的外用糖皮质激素不允许使用。访视前72小时，未经评估不应在皮肤黏膜损害处使用外用药。

7. 研究过程

7.1. 筛选阶段

研究者在获取数据前必须并取得并核实知情同意书。筛选访视必须在随机访视（第 0 周）前 4 周内完成。另外，受试者在宣布筛选 SS 后必须已经接受研究随机分组。

为安全起见或为采集和分析特定样品的技术原因，可能会重复或额外采集样品。

研究者在随机分组前将进行所有用来明确受试者是否符合条件的筛选评估。所有受试者必须满足这些疾病的分类标准。

7.2. 双盲治疗阶段

7.2.1. 第 0 周/随机分组日

在第 0 周，符合条件的受试者将以 1:1 比例被随机双盲分配在人重组白介素-2 组或安慰剂组。

7.2.2. 安慰剂对照的治疗阶段

受试者以同样方式接受低剂量 rhIL-2 或安慰剂治疗。

受试者接受3个疗程的低剂量IL-2或安慰剂。每个疗程予1百万单位低剂量IL-2或安慰剂，皮下注射，隔日给药2周，之后2周停药随访。

7.3. 随访阶段

观察期为 12 周治疗及 12 周随访。收集患者临床表现、自身抗体和其他相关实验室检查，干燥综合征患者进行 ESSDAI 评分评估其病情缓解程度，在治疗前后监测药物不良反应。

7.4. 撤出研究的受试者

撤出研究的受试者不会被召回进行随访评估。

7.5. 有效性评估

有效性评估将基于主要和次要终点结果测评（详见 7.5.3）。描述所有有效性评估和终点、混合终点评估内容的完整列表详见附表 1 和 2。所有有效性评估应被研究者一致进行，以保持前后具有可比性。每次访视都会核实这些数据已采集并确保评估的一致性。

7.5.1. 终点

主要终点

主要终点是 24 周时，ESSDAI 评分改善≥3 分。

主要的次要终点

次要终点包括其他临床应答、安全性和免疫细胞亚群变化（包括 T 细胞和 NK 细胞亚群）。

7.6. 随访

7.6.1. 基线随访

（1）一般资料：包括姓名、性别、年龄、联系电话、病程。

（2）临床表现和体征：包括皮疹、发热、口腔溃疡、泡沫尿、疾病活动评分等。

（3）实验室检查：血常规、肝肾功能、ANA、抗 SSA、抗 SSB、RF、免疫球蛋白、ESR、CRP、T 细胞和 B 细胞亚群、IL-2、TGF-β 和其他细胞因子。

7.6.2. 访视

前 3 个月每 2 周访视 1 次（共 6 次），之后每个月访视 1 次。

（1）根据疾病活动评分（包括 ESSDAI 评分）观察临床表现是否缓解。

（2）监测血清学指标改变：每 4 周，血常规检查，肝肾功能，根据病情检测相关抗体如抗 SSA，抗 SSB，RF，免疫球蛋白，ESR，CRP 评估病情。

（3）流式细胞检测：第 0、2、4、6、8、12、16、20 和 24 周，流式细胞术检测 CD4+T 细胞和 CD19+B 细胞，评估治疗前后的细胞亚群变化率。

（4）细胞因子：在第 0 和第 12 周，通过 ELISA 检测 IL-2、IL-6、TGF-β 和其他细胞因子的水平。

7.6.3. 评估

描述所有有效性评估和终点、混合终点评估内容的完整列表详见附件 2。

7.6.3.1. ESSDAI

EULAR（欧洲抗风湿联盟）组织的国际 SS 专家合作组在 2010 年建立了 2 个广受认可的疾病活动性评分，即基于患者主观症状的（EULAR 干燥综合征患者报告指数）评分和基于患者客观系统性症状的 ESSDAI（干燥综合征疾病活动度指数）评分[4,5]。

筛选时（附件 6）进行 ESSDAI 评分，它是覆盖全身 12 个方面的疾病活动性指数，包括全身症状、淋巴结病变、腺体、皮肤黏膜、关节、肺部、肾脏、肌肉、周围神经、中枢神经、血液系统和生物学特征。每个方面分局活跃程度分为 3-4 级，每个方面的权重由多因素线性回归模型取得。只对跟疾病有关的症状评分，避免长期临床症状的影响。最终评分 0-123 分，0 分代表疾病无活动。

7.6.3.2. 干燥、疲乏、疼痛 VAS 评分

受试者将在一个可视化的模拟尺（VAS：0-100mm）上评估其过去 2 周的平均干燥、疲劳和疼痛程度。量尺的刻度从 0（代表“无症状”）到 100（代表“症状最重”）。

7.6.3.3. MFI-20 评分

问卷由 20 个项目组成，受试者必须在五点量表上标出她特定状态的程度，从“是”到“否”。正向和负向项目的计分相等时，可以相互抵消评分。分数越高，说明疲劳程度越高。

7.6.3.4. 健康状况调查简表 SF-36

RAND 健康状况调查简表（SF）-36 问卷是包含 36 项的自评多维量表。8 个健康维度包含功能评估（附件 3）[7]：

生理功能限制

日常角色活动限制

躯体疼痛

一般精神健康（精神压力和健康）

生命力（能量和疲劳）

因生理或精神问题造成的社会功能限制

因个人或情感问题造成的日常活动限制

一般健康感知

亚类评分从 0 到 100

评分由生理部分总分、精神部分总分、总分、亚类评分组成。评分越高，结果越好。14 岁以上适用，5-10 分钟完成。多数语言有翻译版本。量表已被广泛语言和文化核实。

SF-36 评价的概念不特别针对任何年龄、疾病或治疗组，可以比较不同疾病的相关负担和不同治疗的获益。任何亚类 3 分变化或组成部分 5 分变化是有临床意义的。

7.7. 生物标志物

血液、血清、尿液的收集、准备、储存和运输详见时间和事件表（表 1）和实验室手册。生物标志物包含但不限于 ESR、CRP 和其他炎症标志物、细胞表面标志物、自身抗体如抗 SSA、抗 SSB、RF、CD4+T 细胞和 CD19+B 细胞亚群，细胞因子（含 IL-2、IL-2、IL-6、IL-7、IL-10、IL-12p70、IL-15、IL-17A、IL-21、IFN-γ、TGF-β、IFN-α）和其他可能在 SS 发生发展中涉及的生物标志物种类。

7.8. 样品收集和管理

样品收集的实际日期和时间必须记录在实验室征用表中。

在时间和事件表（附表 2）中记录所有样品收集的时机和频率。收集、管理和运输样品的指导在实验室手册中，用于样品收集和管理。

8. 研究药物信息

8.1. 研究药物的形状描述

重组人白介素-2，其主要成分是重组人白细胞介素-2，来自重组大肠杆菌。辅剂是甘露醇，十二烷基硫酸钠，磷酸氢二钠，磷酸二氢钠，人白蛋白。安慰剂与IL-2具有相同的辅剂。

8.2. 皮下给药

受试者接受低剂量IL-2或安慰剂治疗3个疗程。具体方法为1百万单位，注射2ml，每隔一天皮下注射（rhIL-21×10^6IU，SC，Qod），共14天。停药14天后，开始另一个疗程。

8.3. 包装

研究用药将进行独立包装，以确保在整个供应链过程中对其进行适当管理。

8.4. 标签

研究药物标签将包含符合适用法规要求的信息。

8.5. 准备、处理、储存

所有研究试剂必须在2°C至8°C的受控温度下储存，不得冷冻，并避光。应避免剧烈摇晃产品。在给药之前，应目视检查产品的颗粒物质和变色。如果在溶液中观察到变色（除了浅黄色），可见的不透明颗粒或其他外来颗粒，则不应使用该产品。

在研究材料的制备和施用过程中必须使用无菌操作。在制备和给药过程中应避免暴露在直射阳光下。

8.6. 药物问责

研究者负责确保在整个研究过程中对在现场接收的所有研究药物进行清点和计算。对受试者施用的研究药物必须记录在药物责任表格中。研究现场人员不得将研究药物容器内的东西组合起来。

研究药物必须严格按照方案和容器标签进行处理，并且必须在适当的环境条件下存放在研究地点的限制访问区域或锁定的柜子中。未经使用的研究药物和受试者返回的研究药物必须在现场监测访问期间由赞助商的研究现场监测员进行验证。未使用的返回给保荐人的药物，或使用过的需进行销毁的药物，将被记录在药品退回表中。当研究地点是经过授权的销毁单位并且研究药物供应在现场销毁时，也必须在药物退回表上记录。

潜在危险的材料，如用过的输液袋，针头，注射器和小瓶应立即以安全的方

式处理，因药品责任的目的不能保留。

研究药物应在研究者或研究现场人员的合格成员或医院/诊所药剂师的监督下进行。研究药物仅供参与研究的受试者使用。返回的研究药物不得再次分发，即使是同一受试者。研究药物不得重新标记或重新分配以供其他受试者使用。研究人员一致同意，不得将研究药物从研究地点以外的任何地点分发，也不得存放在与赞助商商定的研究地点。

9. 安全性评价

9.1. 药物不良反应

药物不良反应包括了受试者应用 IL-2 治疗过程中发生的影响受试者健康的任何症状、综合征或疾病，也包括了实验室检查发现或其他诊断过程中发现的临床相关情况。

不良反应可能是：新的疾病；治疗状态症状或体征的恶化；伴随疾病的恶化等。

严重不良反应是指在用药过程中或观察期间任何时候出现的以下不良事件：包括：需住院；可能危害受试者或需要采取措施来预防的一种后果。

9.1.1. 不良反应严重程度判断

不良事件是指在临床研究受试对象在使用了研究 （调查或非调查） 产品中发生的不良的医疗事件。不良事件和治疗之间不一定存在因果关系。因此，不良事件可能是任何不利的和意外的迹象（包括异常发现）、症状或在使用研究（调查或非调查） 产品后出现的相关疾病，但不确切是否和研究 （调查或非调查） 产品存在因果关联。（国际协调会议[ICH]的定义）。

这包括和基线相比发生的任何新发疾病或严重程度或发生频率加重，或诊断过程中出现的异常结果，包括实验室检查异常。

注意：所有不良事件，最后一次不良事件记录的时间）。

严重不良事件

根据 ICH 和欧盟的《药物使用警戒指南》，严重不良事件是指：在任何剂量下发生以下不良医疗事件：

死亡

危及生命 （事发时受试者有生命危险，并不包括假设事件更严重时将造成死亡）

需要住院治疗或延长住院时间。

持续或严重残疾-无行为能力

先天畸形/先天缺陷

通过研究药物发生的任何可疑的感染传播

在医学上是否重要

在迅速做出医学和科学的判断前，需考虑是否适合其他情况，如重要的医疗事件可能不会立即危及生命或导致死亡或住院，但可能危及受试者或需要干预来预防上诉任何一种不良事件的发生。以上均需要慎重考虑。

如果发生了严重和意外的不良事件，且有证据表明研究药物与该事件之间存在因果关系 （例如过敏反应导致的死亡），必须将该事件报告为严重和意外的可疑不良反应。

未列出的（意外的）不良事件/安全参考信息

当研究产品提供的安全信息和事件的性质或严重程度不一致时，则被视为未列出的不良事件。对于 rhIL-2，不良事件的发生机率主要取决于研究者是否将其列在调查人员手册中。

与药物使用相关的不良事件

根据定义，若不良事件和用药之间的关系可能、合理或非常有可能时，则认为两者相关。

9.1.2. 严重程度定义

严重程度分级主要根据以下定义：

轻度：受试者可以忍受，不影响治疗，不需要特别处理，对受试者无影响。

中度：受试者难以忍受，对受试者正常活动有直接影响。

重度：危及受试者生命，影响受试者功能或日常行为能力，需立即撤药或做紧急处理。

研究人员应用临床判断来评估和受试者不直接相关的事件严重性（如实验室异常）。

9.1.3. 药物不良反应的处理措施及相关费用

（1）低剂量注射用 rhIL-2 的常见不良反应在停药后可自行恢复。

（2）全身：当大剂量应用 IL-2 时，发热、寒战较常见，这与剂量相关；恶心、呕吐、流感样症状较少见。一旦出现，可继续观察，多可自行恢复。

（3）注射部位：红肿、硬结、疼痛，可继续观察，多可自行恢复，若持续无缓解，必要时减小剂量或停药。

（4）较大剂量时：毛细血管渗漏综合征、低血压等表现，需立即停药，必要时采用药物对症改善毛细血管通透性及低血压状态。

备注：本研究为小剂量IL-2治疗研究，临床应用和前期研究结果显示，尚未出现过大剂量IL-2引起的不良反应。

10. 统计分析

10.1. 受试者概述

接受过至少 1 次试验药物治疗的受试者均采集基线人口学及临床资料。有效性分析基于意向性治疗分析（mITT），也就是包括所有接受至少 1 次试验药物受试者。同时也进行符合方案集分析（PP），也就是完成治疗及随访受试者。安全性分析基于所有随机且接受至少 1 次治疗受试者。

10.2. 样本量估计

样本量估计基于每组受试者达到原始终点的比例。设定对照组 24 周达到原始终点比例为 30%，低剂量 IL-2 治疗组达到原始终点比例为 75%。各自需 28 例，可达到 90%以上的检验效能，显著性水平为 0.05。

10.3. 有效性分析

有效性分析基于意向性治疗分析（mITT）以及符合方案集分析（PP）。

10.3.1. 主要终点分析

干燥综合征原始终点为 24 周时 ESSDAI 评分改善≥3 分。

矫正基线的逻辑回归模型用于二分类变量的统计。连续变量采用协方差分析模型。纵向数据，对比不同随访时间，治疗有效性的分析，根据变量为连续变量或分类变量，分别采用重复测量混合模型以及广义评估方程。P 小于 0.05 为具有显著统计学意义。

10.3.2. 次要终点

干燥综合征次要终点为临床症状评价，干燥 VAS 评分、疼痛 VAS 评分、干燥 VAS 评分改善≥50mm 的比例；干燥 VAS 评分、疼痛 VAS 评分、干燥 VAS 评分和基线的变化；健康调查简表（SF-36），唾液腺流率以及 ESR），实验室指标包括免疫球蛋白 A，免疫球蛋白 B，抗 SSA 抗体、抗 SSB 抗体、泪膜破裂时间、泪河高度、Schirmer 试验、睑板腺数量、唾液腺超声、安全性评价以及 Treg 细胞、B10 细胞亚群。

连续变量分析采用协方差分析，其中以组别为固定因素，以基线资料作为协变量。如不满足平行性假设条件，应用非参数检验。矫正基线的逻辑回归模型用于二分类变量的统计。纵向数据，对比不同随访时间，治疗有效性的分析，根据变量为连续变量或分类变量，分别采用重复测量混合模型以及广义评估方程。P 小于 0.05 为具有显著统计学意义。

10.3.3. 其他分析

基线人口学以及临床疾病特征分析采用 t 检验、非参数检验比较连续变量，

卡方检验比较分类变量。统计分析将采用 SPSS 17.0 统计分析软件进行计算。所有的统计检验均采用双侧检验，P 值小于或等于 0.05 将被认为所检验差别有统计意义。可信区间采用 95%的可信度。

脱落分析：采用卡方检验计算脱落率。逐例描述各脱落受试者的人口学资料，主要指标和各时间点观察值。

10.4. 安全性分析

接受至少 1 次试验药物的受试者均纳入安全性分析。治疗过程中所有不良事件均纳入分析。针对每一个不良事件，至少发生 1 次的受试者所占比例进行统计。本研究分析感染的发生率以及感染类型，同时分析注射反应。导致退组的不良事件或者严重不良事件将格外重视。

11. 数据质量保证/质量控制

为确保数据的准确性和可靠性而采取的步骤包括选择合格的研究人员和适当的研究地点，在研究之前与研究人员和研究现场人员一起审议程序，由保荐人定期监测访问，以及直接传播临床实验室数据从中心实验室进入赞助商的数据库。将提供书面说明，用于样品的收集，处理，存储和运输。

在研究开始之前，将向研究现场人员提供CRF完成指南并进行审查。赞助商将在现场监测访问期间以及传输给赞助商之后审查CRF的准确性和完整性;任何差异将由调查员或指定人员酌情解决。在将数据上载到研究数据库之后，将验证它们与数据源的准确性和一致性。

12. 数据管理

研究人员应准确，及时地记录纸质和电子数据库版的病例数据。

13. 道德规范

经北京大学人民医院伦理委员会批准，所有受试者均获得知情同意书。

14. 总结和数据保存

保留原始记录。

根据数据库进行数据汇总，统计单位负责统计处理。知情同意书和CRF表将保留至研究结束后的5年。

附表：

1. 试验治疗期随访流程图

阶段	筛选期	治疗期（24 周）									
访视	V0	V1	V2	V3	V4	V5	V6	V7	V8	V9	V10
时间	-4-0 周	0 周	2 周 ±3 天	4 周 ±3 天	6 周 ±3 天	8 周 ±3 天	10 周±3 天	12 周±3 天	16 周±3 天	20 周±3 天	24 周±3 天
签署知情同意书[a]	√										
病史采集	√										
入选标准		√									
排除标准		√									
血常规[b]	√		√	√	√	√	√	√	√	√	√
尿常规[c]	√							√			√
肾小管功能[d]	√							√			√
尿 HCG[e]	√										
乙肝表面抗原	√										
丙肝抗体	√										
肝功能[f]	√							√			√
肾功能[g]	√							√			√
血脂[h]	√										
血糖	√										
电解质[i]	√							√			√
ESR	√							√			√
CRP	√							√			√
免疫球蛋白/补体	√		√	√	√	√	√	√	√	√	√
自身抗体[k]	√				√			√			√
细胞因子[l]		√						√			
心电图	√										
眼科检查	√							√			√
肺功能[m]	√							√			√
唇腺活检[n]	√										
唾液腺彩超	√							√			√
发放及回收药		√	√	√	√	√	√				

物											
不良事件情况记录		√	√	√	√	√	√	√	√	√	√
ESSDAI 评分		√						√			√
MFI-20 评分		√						√			√
VAS 评分		√						√			√
SF-36 评分		√						√			√
CD4$^+$T 细胞		√	√	√	√	√	√	√	√	√	√
CD19+B 细胞		√	√	√	√	√	√	√	√	√	√
日记卡发放与回收		√									√

注：

a 筛选须在随机分组（基线 0 周）前的 4 周内进行。病史采集包括人口学资料、诊断、生命体征、是否初治、家族史、吸烟史、饮酒史、过敏史、主要病史、用药史、体格检查。

b 血常规：血红蛋白、红细胞比容、白细胞分类（嗜碱性粒细胞、嗜酸性粒细胞、淋巴细胞、单核细胞、中性粒细胞）、血小板。

c 尿常规：尿红细胞、白细胞、上皮细胞、结晶、颗粒管型、细菌。

d 肾小管功能：包括 β2 微球蛋白、N-乙酰氨基葡萄糖苷酶活性、视黄醇结合蛋白，必要时检查。

e 尿妊娠试验：育龄期女性必查。

f 肝功能：谷丙转氨酶、谷草转氨酶、白蛋白、总蛋白。

g 肾功能：肌酸激酶、尿素氮（BUN）、血肌酐、肾小球滤过率（eGFR）。

h 血脂：总胆固醇、甘油三酯、低密度脂蛋白胆固醇、高密度脂蛋白胆固醇。

i 电解质：钠、钾、钙、氯、二氧化碳结合力。

j 免疫球蛋白/补体：IgA，IgM，IgG，γ 球蛋白，C3，C4。

k 自身抗体：ANA、抗 SSA 抗体、抗 SSB 抗体、RF。

l 细胞因子：IL-2、TNF-α、IFN-γ、IL-6、IL-21、IL-17、TGF-β、sIL-2Rα 等。

m 肺功能：必要时。

n 唇腺活检：必要时。

2. VAS 评分

（1） 过去 2 周的干燥症状：包括口、眼、皮肤、鼻、气管及阴道。

无	0	1	2	3	4	5	6	7	8	9	10	有

（2） 过去 2 周的疲乏程度。

无											有
	0	1	2	3	4	5	6	7	8	9	10

（3） 过去 2 周的肢体疼痛程度。

无											有
	0	1	2	3	4	5	6	7	8	9	10

最终评分为干燥症状、肢体疼痛、疲乏三个积分的平均值，范围为 0-10 分。

3. 欧洲干燥综合征疾病活动度表（ESSDAI）

**全身症状[3]**<br>*注意除外与疾病无关的系统症状：如原发感染性发热，减肥所致体重下降。*		
**不活动**	无以下任何症状	☐
**低活动度**	轻微或间断发热（37.5°-38.5°C）或夜间盗汗<br>体重下降 5%到 10%	☐
**中活动度**	高热（>38.5°C）或夜间盗汗<br>体重下降大于 10%	☐

**淋巴结病变[4]**		
**不活动**	无以下任何症状	☐
**低活动度**	-任意部位淋巴结≥1cm 或腹股沟区淋巴结≥2cm	☐
**中活动度**	-任意部位淋巴结≥2cm 或腹股沟区淋巴结≥3cm<br>- 和/或脾脏肿大（临床可触及或影像学发现肿大）	☐
**高活动度**	-存在恶性 B 细胞增殖	☐

**腺体病变[2]***注意除外与疾病无关的腺体肿胀,如结石,感染*		
**不活动**	无腺体肿大	☐
**低活动度**	轻度腺体肿大:<br>- 腮腺肿大（≤ 3cm），<br>- 或局限性下颌下腺或泪腺肿大[1]	☐
**中活动度**	重度腺体肿大:<br>- 腮腺肿大（>3cm ）<br>- 或广泛下颌下腺或泪腺肿大[1]	☐

[1] 局限性和广泛性下颌下腺,泪腺肿大的区别由临床医生通过体检判断

**关节病变[2]**<br>*注意除外与疾病无关的关节受累,如骨关节炎*		
**不活动**	目前没有活动性关节受累	☐
**低活动度**	手,腕,踝及足关节疼痛伴晨僵（>30 min）	☐
**中活动度**	28 个关节中有 1 到 5 个滑膜炎	☐
**高活动度**	28 个关节中≥ 6 个滑膜炎	☐

**粘膜病变[3]**<br>*注意：与损伤相关的粘膜病变归为不活动,排除与疾病活动度无关的粘膜病变*		
**不活动**	目前无活动性粘膜受累	☐
**低活动度**	多形红斑	☐
**中活动度**	局限性皮肤血管炎,包括荨麻疹性血管炎 [2],<br>或足及踝部紫癜,<br>或亚急性皮肤狼疮	☐
**高活动度**	弥漫性皮肤血管炎,包括荨麻疹性血管炎 [2],<br>或弥漫性紫癜<br>或与血管炎相关的溃疡	☐

[2] 局限性皮肤血管炎累及小于体表面积的 18%; 弥漫性皮肤血管炎累及大于体表面积 18% ;

体表面积（Body surface area,BSA）用九分区法定义（通常用来评估烧伤面积）,具体如下:手掌（不包括手指）占1% BSA; 每个下肢占18% BSA;每个上肢占9% BSA;躯干（前面）占 18% BSA; 躯干 （后背） 占 18% BSA

**肺部病变[5]**<br>*注意：损作所致的肺部病变归为不活动性病变，除外与呼吸系统受累无关的病变（如吸烟）*		
**不活动**	目前无活动性肺部病变	☐
**低活动度**	持续咳嗽或支气管受累,但 X 线无异常表现<br>或放射学或 HRCT 诊断的肺间质病变 [3]:<br>• 无呼吸困难<br>• 肺功能正常	☐
**中活动度**	中度活动性肺部病变, 如 HRCT 诊断的肺间质病变:<br>• 活动后气短（NHYA II）<br>• 肺功能异常:<br>- 40%≤ DL_{CO}＜70%和/或 60% ≤ FVC ＜80%	☐

**高活动度**	高度活动性肺间质病变,如 HRCT 诊断的肺间质病变:<br>• 休息时气短（NHYA III, IV）<br>• 或肺功能异常:<br>- DL_{CO}< 40% 和/或 FVC < 60%	☐

[3]HRCT 及放射学诊断的肺间质病变需要是 2 年内新诊断的.

DLCO=CO 扩散能力; FVC= 用力肺活量; HRCT= 高分辨率 CT; NHYA=组约心脏病协会分类

**肾脏疾病[5]**<br>*注意:与损伤相关的稳定的病变归为不活动性病变，并排除与肾脏疾病无关的病变*<br>如有肾活检结果,则首先按照组织特点确定疾病活动性.		
不活动	目前无活动性肾脏病变:<br>- 蛋白尿< 0.5g/d, 无血尿,无白细胞尿,无酸中毒.<br>- 或由于损伤所致的持续稳定的蛋白尿.	☐
低活动度	肾脏活动性病变, 包括:<br>• 肾小管酸中毒不伴肾功能损害（GFR[4]≥60ml/min）<br>• 肾小球病变:<br>- 蛋白尿 （0.5g/d 到 1 g/d）<br>- 无血尿及肾功能衰竭 （GFR4 ≥60ml/min）	☐
中活动度	中度肾脏活动性病变,如：<br>• 肾小管酸中毒伴肾功能衰竭（GFR[4]< 60 ml/min）<br>• 肾小球病变：<br>- 蛋白尿 1g/d 到 1.5g/d<br>- 无蛋白尿及肾功能衰竭 （GFR4 ≥ 60ml/min）<br>• 组织学证据：<br>- 外膜性肾小球肾炎<br>- 严重的间质淋巴细胞浸润	☐
高活动度	高度活动性肾脏病变,如:<br>• 肾小球病变：<br>- 蛋白尿大于 1.5 g/d<br>- 或血尿<br>- 或肾功能衰竭（GFR[4]< 60 ml/min）<br>• 或组织学证据：<br>- 增生性肾小球肾炎<br>- 冷球蛋白相关的肾功能病变	☐

[4] MDRD 公式法估算肾小球滤过率（GFR）

**肌肉病变[6]**<br>*注意排除与疾病无关的肌肉受累,如糖皮质激素引起的肌无力*

**不活动**	目前无活动性肌肉病变	☐
**低活动度**	肌电图或肌肉活检诊断的活动性肌炎:<br>- 无肌无力<br>- 肌酸肌酶 （N < CK ≤ 2N）	☐
**中活动度**	经 EMG 或肌肉活检证实的中度活动性肌炎：<br>- 肌无力（肌力大于等于 4 级）<br>- 或肌酸肌酶升高（2N < CK ≤ 4N）	☐
**高活动度**	经 EMG 或肌肉活检证实的高度活动性肌炎:<br>- 肌无力（肌力≤ 3/5）<br>- 或肌酸肌酶升高 （> 4N）	☐

EMG= electromyogram, 肌电图;

**外周神经病变[5]**<br>*注意：与损伤相关的稳定的病变归为不活动性病变，并排除与疾病无关的 PNS 病变*		
**不活动**	目前无活动性外周神经病变	☐
**低活动度**	活动性外周神经病变,如:<br>- - NCS 证实的单纯感觉轴索神经病变<br>- - 三叉神经（V）痛	☐
**中活动度**	NCS 证实的中度活动性外周神经病变，如：<br>- 轴索感觉-运动神经病变伴运动功能缺失≤4/5<br>- 单纯感觉神经病变伴冷球蛋白血症型血管炎<br>- 神经节病变 [5] 所致的轻/中度运动失调<br>- 炎症性脱髓鞘性多发神经病变 [6] （CIDP）伴轻度功能障碍 （运动功能缺失最多为 4/5 或轻度运动失调）<br>或脑神经的外周病变 （三叉神经（V）痛除外）	☐
**高活动度**	NCS 证实的高度活动性外周神经病变，如：<br>- 轴索感觉-运动神经病变伴运动功能缺失≤ 3/5<br>- 血管炎导致的外周神经病变（复合性单神经炎等）<br>- 神经节病变导致的重度共济性运动失调 [5]<br>- 炎症性脱髓鞘性多发神经病变 [6] （CIDP）伴重度功能障碍：运动功能缺失≤ 3/5 或重度运动失调	☐

NCS: 神经传导检查.

[5] 单纯感觉障碍伴运动失调和弥散性功能障碍或感觉缺失

[6]多神经根病变存在以下 4 种临床症状（四肢感觉运动缺失，近端运动障碍，广义的无反射，上肢感觉异常和/或相关的颅神经病变），蛋白水平升高和/或NCS异常（远端运动电位潜伏期延长，神经传导速度下降,F波潜伏期延长，传导阻滞和/或时间上的离散）

**中枢神经病变[5]**<br>*注意：与损伤相关的稳定的病变归为不活动性病变，并排除与疾病无关的 CNS 病变*		
**不活动**	目前无活动性 CNS 病变	☐
**中活动度**	中度活动性 CNS 病变，如：<br>-颅神经的中枢病变<br>-视神经炎<br>-多发性硬化样综合征出现单纯感觉障碍或知觉障碍	☐
**高活动度**	高度活动性 CNS 病变，如：<br>- 脑血管炎伴意外或短暂失血发作<br>- 失神小发作 s<br>- 横贯性脊髓炎<br>- 淋巴细胞性脑膜炎<br>-多发性硬化样综合征出现运动功能缺失	☐

**血液系统[2]**<br>*注意：*<br>*- 仅包括自身免疫性贫血[7],血小板减少[8] 和中性粒细胞减少症[9]*<br>*- 排除与疾病无关的血细胞减少症 （ 如维生素或铁缺乏引起的血细胞减少或药物性血细胞减少,如环磷酰胺引起的淋巴细胞减少）*		
**不活动**	无自身免疫性血细胞减少	☐
**低活动度**	自身免疫性血细胞减少：<br>-中性粒细胞减少症 （1000 <中性粒细胞< 1500/mm3）<br>- 或贫血 （10 <Hb < 12g/dl）<br>-或血小板减少症（ 100,000 <血小板< 150,000/mm3）<br>或淋巴细胞减少症（500<淋巴细胞<1000/mm3）	☐
**中活动度**	自身免疫性血小板减少：<br>- 中性粒细胞减少（500 ≤ 中性粒细胞 ≤ 1000/mm3），<br>-或贫血 （8 ≤ Hb ≤ 10g/dl）<br>-或血小板减少症（50,000 ≤ 血小板 ≤ 100,000/mm3）<br>或淋巴细胞减少症（≤500/mm3）	☐
**高活动度**	自身免疫性血细胞减少：<br>-中性粒细胞减少症 （中性粒细胞< 500/mm3），<br>- 或贫血（Hb < 8 g/dl）<br>- 或血小板减少症（血小板 < 50,000/mm3）	☐

[7] Coombs 试验阳性伴网织红细胞增多的贫血

8 无其它原因的循环血小板减少，或抗血小板自身抗体阳性和/或骨髓活检有巨噬细胞存在和/或相关的自身免疫性贫血

[9] 无其它原因的中性粒细胞减少症。

**生物学特征[1]**		
**不活动**	无以下任何生物学特征	☐
**低活动度**	-补体正常<br>-或低补体血症 （C4 ，C3 or CH50 低）<br>- 或高丙球蛋白血症或 16 g/L <IgG<20g/L	☐
**中活动度**	- 冷球蛋白血症<br>- 或高丙球蛋白血症或 IgG> 20g/L<br>- 近期 [10] 发生的低丙球蛋白血症或 IgG 减少（<5g/L）	☐

[10] 最近 6 个月

计分方法:

每个系统的得分按该系统活动度来评，在其后对应的方括号内。
活动水平按如下方法评分:
不活动= 0
低活动度 = 1
中等活动度 = 2
高活动度 = 3
总分是各个系统病变得分之和。

4. SF-36 生活质量表调查表

1. 总体来讲，您的健康状况是：
① 非常好 ② 很好 ③ 好 ④ 一般 ⑤ 差
2. 跟 1 年以前比，您觉得自己的健康状况是：
① 比 1 年前好多了 ② 比 1 年前好一些 ③ 跟 1 年前差不多
④ 比 1 年前差一些 ⑤ 比 1 年前差多了
（权重或得分依次为 1、2、3、4 和 5）

[健康和日常活动]

3. 以下这些问题都和日常活动有关。请您想一想，您的健康状况是否限制了这些活动?如果有限制，程度如何？
（1）重体力活动，如跑步举重、参加剧烈运动等：
① 限制很大 ② 有些限制 ③ 毫无限制
（权重或得分依次为 1、2、3，下同）
（2）适度的活动，如移动一张桌子、扫地、打太极拳、做简单体操等：
① 限制很大 ② 有些限制 ③ 毫无限制
（3）手提日用品，如买菜、购物等：
① 限制很大 ② 有些限制 ③ 毫无限制
（4）上几层楼梯：
① 限制很大 ② 有些限制 ③ 毫无限制
（5）上一层楼梯：
① 限制很大 ② 有些限制 ③ 毫无限制

（6）弯腰、屈膝、下蹲：
① 限制很大 ② 有些限制 ③ 毫无限制
（7）步行 1500 米以上的路程：
① 限制很大 ② 有些限制 ③ 毫无限制
（8）步行 1000 米的路程：
① 限制很大 ② 有些限制 ③ 毫无限制
（9）步行 100 米的路程：
① 限制很大 ② 有些限制 ③ 毫无限制
（10）自己洗澡、穿衣：
① 限制很大 ② 有些限制 ③ 毫无限制

4. 在过去 4 个星期里，您的工作和日常活动有无因为身体健康的原因而出现以下这些问题？
（1）减少了工作或其他活动时间：
① 是 ② 不是
（权重或得分依次为 1、2，下同）
（2）本来想要做的事情只能完成一部分：
① 是 ② 不是
（3）想要干的工作或活动种类受到限制：
① 是 ② 不是
（4）完成工作或其他活动困难增多（比如需要额外的努力）：
① 是 ② 不是

5. 在过去 4 个星期里，您的工作和日常活动有无因为情绪的原因（如压抑或忧虑）而出现以下这些问题？
（1）减少了工作或活动时间：
① 是 ② 不是
（权重或得分依次为 1、2，下同）
（2）本来想要做的事情只能完成一部分：
① 是 ② 不是
（3）做事情不如平时仔细：
① 是 ② 不是

6. 在过去 4 个星期里，您的健康或情绪不好在多大程度上影响了您与家人、朋友、邻居或集体的正常社会交往？
① 完全没有影响 ② 有一点影响 ③ 中等影响 ④ 影响很大 ⑤ 影响非常大
（权重或得分依次为 5、4、3、2、1）

7. 在过去 4 个星期里，您有身体疼痛吗？
① 完全没有疼痛 ② 有一点疼痛 ③ 中等疼痛 ④ 严重疼痛 ⑤ 很严重疼痛
（权重或得分依次为 6、5.4、4.2、3.1、2.2、1）

8. 在过去 4 个星期里，您的身体疼痛影响了您的工作和家务吗？
① 完全没有影响 ② 有一点影响 ③ 中等影响 ④ 影响很大 ⑤ 影响非常大
（如果 7 无 8 无，权重或得分依次为 6、4.75、3.5、2.25、1.0；如果为 7 有 8 无，则为 5、4、3、2、1）

[您的感觉]

9. 以下这些问题是关于过去 1 个月里您自己的感觉，对每一条问题所说的事情，您的情况是什么样的？

（1）您觉得生活充实：
① 所有的时间 ② 大部分时间 ③ 比较多时间 ④ 一部分时间
⑤ 小部分时间 ⑥ 没有这种感觉
（权重或得分依次为 6、5、4、3、2、1）
（2）您是一个敏感的人：
① 所有的时间 ② 大部分时间 ③ 比较多时间 ④ 一部分时间
⑤ 小部分时间 ⑥ 没有这种感觉
（权重或得分依次为 1、2、3、4、5、6）
（3）您的情绪非常不好，什么事都不能使您高兴起来：
① 所有的时间 ② 大部分时间 ③ 比较多时间 ④ 一部分时间
⑤ 小部分时间 ⑥ 没有这种感觉
（权重或得分依次为 1、2、3、4、5、6）
（4）您的心理很平静：
① 所有的时间 ② 大部分时间 ③ 比较多时间 ④ 一部分时间
⑤ 小部分时间 ⑥ 没有这种感觉
（权重或得分依次为 6、5、4、3、2、1）
（5）您做事精力充沛：
① 所有的时间 ② 大部分时间 ③ 比较多时间 ④ 一部分时间
⑤ 小部分时间 ⑥ 没有这种感觉
（权重或得分依次为 6、5、4、3、2、1）
（6）您的情绪低落：
① 所有的时间 ② 大部分时间 ③ 比较多时间 ④ 一部分时间
⑤ 小部分时间 ⑥ 没有这种感觉
（权重或得分依次为 1、2、3、4、5、6）
（7）您觉得筋疲力尽：
① 所有的时间 ② 大部分时间 ③ 比较多时间 ④ 一部分时间
⑤ 小部分时间 ⑥ 没有这种感觉
（权重或得分依次为 1、2、3、4、5、6）
（8）您是个快乐的人：
① 所有的时间 ② 大部分时间 ③ 比较多时间 ④ 一部分时间
⑤ 小部分时间 ⑥ 没有这种感觉
（权重或得分依次为 6、5、4、3、2、1）
（9）您感觉厌烦：
① 所有的时间 ② 大部分时间 ③ 比较多时间 ④ 一部分时间
⑤ 小部分时间 ⑥ 没有这种感觉
（权重或得分依次为 1、2、3、4、5、6）
10. 不健康影响了您的社会活动（如走亲访友）：
① 所有的时间 ② 大部分时间 ③ 比较多时间 ④ 一部分时间
⑤ 小部分时间 ⑥ 没有这种感觉
（权重或得分依次为 1、2、3、4、5、6）

[总体健康情况]

11. 请看下列每一条问题，哪一种答案最符合您的情况?
（1）我好象比别人容易生病：
① 绝对正确 ② 大部分正确 ③ 不能肯定 ④ 大部分错误 ⑤ 绝对错误`

（权重或得分依次为 1、2、3、4、5）
（2）我跟周围人一样健康：
① 绝对正确 ② 大部分正确 ③ 不能肯定 ④ 大部分错误 ⑤ 绝对错误
（权重或得分依次为 5、4、3、2、1）
（3）我认为我的健康状况在变坏：
① 绝对正确 ② 大部分正确 ③ 不能肯定 ④ 大部分错误 ⑤ 绝对错误
（权重或得分依次为 1、2、3、4、5）
（4）我的健康状况非常好：
① 绝对正确 ② 大部分正确 ③ 不能肯定 ④ 大部分错误 ⑤ 绝对错误
（权重或得分依次为 5、4、3、2、1）
SF-36 生活质量评分统计

题号	计分	题号	计分	题号	计分	题号	计分
1		3-9		7		9-9	
2		3-10		8		10	
3-1		4-1		9-1		11-1	
3-2		4-2		9-2		11-2	
3-3		4-3		9-3		11-3	
3-4		4-4		9-4		11-4	
3-5		5-1		9-5			
3-6		5-2		9-6			
3-7		5-3		9-7			
3-8		6		9-8		合计	

5. MFI-20 评分

多维疲劳量表（multidimensional fatigue inventory-20,MFI-20）

题号.	项目	是 1	2	3	4	5 否
1	我感觉良好	□	□	□	□	□
2	我感觉只能做一点体力活动	□	□	□	□	□
3	我感觉很有活力	□	□	□	□	□
4	我愿做各种令我开心的事	□	□	□	□	□
5	我觉得疲惫	□	□	□	□	□
6	我觉得我一天干太多的活	□	□	□	□	□
7	我能专心做事	□	□	□	□	□
8	在体力上我能做很多事	□	□	□	□	□
9	我害怕必须做事	□	□	□	□	□
10	我一天只能做很少的事	□	□	□	□	□
11	我能很好地集中精力	□	□	□	□	□
12	我一直在休息	□	□	□	□	□
13	我要很努力才能集中精神	□	□	□	□	□

14	我要很努力才能应对糟糕的处境	□	□	□	□	□
15	我有很多工作计划	□	□	□	□	□
16	我容易觉得疲劳	□	□	□	□	□
17	我几乎没做任何事	□	□	□	□	□
18	我不想做任何事	□	□	□	□	□
19	我容易走神	□	□	□	□	□
20	我感觉体力状况很好	□	□	□	□	□

--请在每个条目后选择该表述与您相符的程度，越符合越偏向 1 侧，越不符合越偏向 5 侧:

--表述疲劳的条目 2、5、9、10、13、14、 16、17、18、19，为正向计分；

--不表述疲劳的条目 1、3、4、6、7、8、 11、12、15、20，为反向计分；

--其分数越高,说明疲劳症状越严重

References:

1. Reksten TR, Jonsson MV, Szyszko EA, Brun JG, Jonsson R, Brokstad KA. Cytokine and autoantibody profiling related to histopathological features in primary Sjogren's syndrome. Rheumatology (Oxford). 2009; **48**(9): 1102-6.
2. Ramos-Casals M, Brito-Zeron P, Siso-Almirall A, Bosch X, Tzioufas AG. Topical and systemic medications for the treatment of primary Sjogren's syndrome. Nature reviews Rheumatology. 2012; **8**(7): 399-411.
3. Vitali C, Bombardieri S, Jonsson R, Moutsopoulos HM, Alexander EL, Carsons SE, et al. Classification criteria for Sjogren's syndrome: a revised version of the European criteria proposed by the American-European Consensus Group. Annals of the rheumatic diseases. 2002; **61**(6): 554-8.
4. Seror R, Ravaud P, Mariette X, Bootsma H, Theander E, Hansen A, et al. EULAR Sjogren's Syndrome Patient Reported Index (ESSPRI): development of a consensus patient index for primary Sjogren's syndrome. Annals of the rheumatic diseases. 2011; **70**(6): 968-72.
5. Seror R, Ravaud P, Bowman SJ, Baron G, Tzioufas A, Theander E, et al. EULAR Sjogren's syndrome disease activity index: development of a consensus systemic disease activity index for primary Sjogren's syndrome. Annals of the rheumatic diseases. 2010; **69**(6): 1103-9.
6. Smets EM, Garssen B, Bonke B, De Haes JC. The Multidimensional Fatigue Inventory (MFI) psychometric qualities of an instrument to assess fatigue. Journal of psychosomatic research. 1995; **39**(3): 315-25.
7. Ware JE, Jr., Sherbourne CD. The MOS 36-item short-form health survey (SF-36). I. Conceptual framework and item selection. Medical care. 1992; **30**(6): 473-83.

北京大学人民医院医学伦理委员会
伦理审查批件

批件号	2014PHB087-01			
项目名称	低剂量重组人 IL-2 在自身免疫病治疗中作用的临床和机制研究			
项目来源	北京双鹭药业股份有限公司			
研究单位	北京大学人民医院		承担科室	风湿免疫科
主要研究者	栗占国		职　　称	主任医师
审查类别		审查方式	审查日期	
初始审查		会议审查	2014-9-18	
初审后的复审		会议审查	2015-01-20	
复审后的复审		快速审查	2015-08-18	
审查地点	北京市西城区西直门南大街 11 号 北京大学人民医院医学伦理委员会			
审查委员	鲍永珍　陈凤荣　葛子钢　何权瀛　刘开彦　母　双　饶慧瑛　王晓峰<br>王　伟　杨拔贤　曾超美			
审批文件	1. 初始审查申请：2014-09-03<br>2. 初审后的复审申请：2014-12-10<br>3. 复审后的复审申请：2015-02-25<br>4. 临床研究方案：IL002-V2.0，2014-11-29<br>5. 知情同意书：IL002-V3.0，2015-02-25<br>6. 主要研究者简历<br>7. 研究药物说明书			

审查意见：同　意

根据卫生部《涉及人的生物医学研究伦理审查办法（试行）》（2007）、CFDA《药物临床试验质量管理规范（2003）》、《药物临床试验伦理审查工作指导原则（2010）》、《医疗器械临床试验规定（2004）》、《体外诊断试剂临床研究技术指导原则（2007）》、WMA《赫尔辛基宣言》和 CIOMS《人体生物医学研究国际道德指南》的伦理原则，经本伦理委员会审查，同意按所批准的文件开展本项研究。

请遵循 GCP 原则、遵循伦理委员会批准的方案开展临床研究，保护受试者的健康与权利。

研究过程中若变更主要研究者，对临床研究方案、知情同意书、招募材料等的任何修改，请申请人提交修正案审查申请。

发生严重不良事件，请申请人及时提交严重不良事件报告。

请按照伦理委员会规定的年度/定期跟踪审查频率，按期提交研究进展报告；申办者应当向组长单位伦理委员会提交各中心研究进展的汇总报告；当出现任何可能显著影响试验进行、或增加受试者危险的情况时，请申请人及时向伦理委员会提交书面报告。

研究纳入了不符合纳入标准或符合排除标准的受试者，符合中止试验规定而未让受试者退出研究，给予错误治疗或剂量，给予方案禁止的合并用药等没有遵从方案开展研究的情况；或可能对受试者的权益/健康、以及研究的科学性造成不良影响等违背 GCP 原则的情况，请申办者/监查员/研究者提交违背方案报告。

申请人暂停或提前终止临床研究，请及时提交暂停/终止研究报告。

完成临床研究，请申请人提交研究完成报告。

年度/定期跟踪审查频率	12 个月		
批件有效期	1 年；截止日期：2016 年 08 月 20 日<br>（如试验逾期未实施，需提出延长有效期申请）		
联 系 人	丛翠翠	联系电话	010-88324516
主任委员签字	[signature]		
伦理委员会	北京大学人民医院医学伦理委员会　（盖章）		
日　期	2015-08-21		

北京大学人民医院医学伦理委员会
伦理审查批件

批 件 号	2014PHB087-03		
项目名称	低剂量重组人白介素-2（商品名：欣吉尔）在自身免疫疾病治疗中的临床和机制研究		
项目来源	北京双鹭药业股份有限公司		
研究单位	北京大学人民医院	承担科室	风湿免疫科
主要研究者	栗占国	职　　称	教　授

审查类别	审查方式	审查日期
修正案审查	快速审查	2016-08-03

审查地点	北京市西城区西直门南大街 11 号　北京大学人民医院医学伦理委员会
审查委员	饶慧瑛
审批文件	1. 修正案审查申请：2016-05-30<br>2. 研究方案：IL003-4.0 版，2016-05-30

审查意见：同 意			
根据卫生部《涉及人的生物医学研究伦理审查办法（试行）》（2007）、CFDA《药物临床试验质量管理规范（2003）》、《药物临床试验伦理审查工作指导原则（2010）》、《医疗器械临床试验规定（2004）》、《体外诊断试剂临床研究技术指导原则（2007）》、WMA《赫尔辛基宣言》和 CIOMS《人体生物医学研究国际道德指南》的伦理原则，经本伦理委员会审查，同意按所批准的文件开展本项研究。<br>请遵循 GCP 原则、遵循伦理委员会批准的方案开展临床研究，保护受试者的健康与权利。<br>研究过程中若变更主要研究者，对临床研究方案、知情同意书、招募材料等的任何修改，请申请人提交修正案审查申请。<br>发生严重不良事件，请申请人及时提交严重不良事件报告。<br>请按照伦理委员会规定的年度/定期跟踪审查频率，按期提交研究进展报告；申办者应当向组长单位伦理委员会提交各中心研究进展的汇总报告；当出现任何可能显著影响试验进行、或增加受试者危险的情况时，请申请人及时向伦理委员会提交书面报告。<br>研究纳入了不符合纳入标准或符合排除标准的受试者，符合中止试验规定而未让受试者退出研究，给予错误治疗或剂量，给予方案禁止的合并用药等没有遵从方案开展研究的情况；或可能对受试者的权益/健康、以及研究的科学性造成不良影响等违背 GCP 原则的情况，请申办者/监查员/研究者提交违背方案报告。<br>申请人暂停或提前终止临床研究，请及时提交暂停/终止研究报告。<br>完成临床研究，请申请人提交研究完成报告。			
年度/定期跟踪审查频率	12 个月		
批件有效期	1 年；截止日期：2017 年 08 月 02 日<br>（如试验逾期未实施，需提出延长有效期申请）		
联系人	丛翠翠	联系电话	010-88324516
主任委员签字	[illegible]		
伦理委员会	北京大学人民医院医学伦理委员会 （盖章）		
日期	2016-08-03		
