# Supplementary material for: Efficacy and Safety of Low-Dose Interleukin 2 for Primary Sjögren Syndrome: A Randomized Clinical Trial
Source: JAMA Netw Open. 2022 Nov 10;5(11):e2241451. doi: 10.1001/jamanetworkopen.2022.41451 (PMC9650609; doi:10.1001/jamanetworkopen.2022.41451)
Supplement: Supplement 2. — eFigure 1. Phenotypic Characterization of Treg and CD24hiCD27+B Cells After LD-IL-2 Therapy eFigure 2. Flow Cytometry Results Showing CD25 Expression on Different B Cell Subsets and the Changes of CD24hiCD27+ B Cells and Cytokines After Co-Stimulated With IL-2 In Vitro eFigure 3. Immunological Responses to LD-IL-2 Therapy eFigure 4. Serum BAFF Expression on SS and HC eFigure 5. Changes of Serum IFN-α in Patients With Fatigue eTable 1. Inclusion and Exclusion Criteria eTable 2. Adverse Events in Participants Who Received LD-IL-2 or Placebo Treatment eTable 3. Monoclonal Antibodies Used in Flow Cytometric Analysis in Human eTable 4. Decreased Scores From Baseline at Week 12 and Week 24 in Every Domain of ESSDAI eTable 5. Responses of Laboratory Parameters in Participants eTable 6. Percentage of Clinical Manifestations in Different Time Points of Both Groups eTable 7. Change in Ocular Parameters after LD-IL-2 Therapy eTable 8. Change in Salivary Gland Ultrasonography Scan Score After LD-IL-2 Therapy eTable 9. Immunological Responses of T Cell Subtypes to LD-IL-2 Therapy eTable 10. Change in Cytokines After LD-IL-2 Therapy eTable 11. Changes of B Cell Subsets After LD-IL-2 Therapy [file jamanetwopen-e2241451-s002.pdf]

Supplemental Online Content

He J, Chen J, Miao M, et al. Efficacy and safety of low-dose interleukin-2 for primary Sjögren syndrome: a randomized clinical trial. *JAMA Netw Open.* 2022;5(11):e2241451. doi:10.1001/jamanetworkopen.2022.41451

eFigure 1. Phenotypic Characterization of Treg and $CD24^{hi}CD27^{+}$B Cells After LD-IL-2 Therapy

eFigure 2. Flow Cytometry Results Showing CD25 Expression on Different B Cell Subsets and the Changes of $CD24^{hi}CD27^{+}$ B Cells and Cytokines After Co-Stimulated With IL-2 In Vitro

eFigure 3. Immunological Responses to LD-IL-2 Therapy

eFigure 4. Serum BAFF Expression on SS and HC

eFigure 5. Changes of Serum IFN-α in Patients With Fatigue

eTable 1. Inclusion and Exclusion Criteria

eTable 2. Adverse Events in Participants Who Received LD-IL-2 or Placebo Treatment

eTable 3. Monoclonal Antibodies Used in Flow Cytometric Analysis in Human

eTable 4. Decreased Scores From Baseline at Week 12 and Week 24 in Every Domain of ESSDAI

eTable 5. Responses of Laboratory Parameters in Participants

eTable 6. Percentage of Clinical Manifestations in Different Time Points of Both Groups

eTable 7. Change in Ocular Parameters after LD-IL-2 Therapy

eTable 8. Change in Salivary Gland Ultrasonography Scan Score After LD-IL-2 Therapy

eTable 9. Immunological Responses of T Cell Subtypes to LD-IL-2 Therapy

eTable 10. Change in Cytokines After LD-IL-2 Therapy

eTable 11. Changes of B Cell Subsets After LD-IL-2 Therapy

This supplemental material has been provided by the authors to give readers additional information about their work.

© 2022 He J et al. *JAMA Network Open.*

eFigure 1. Phenotypic Characterization of Treg and CD24^{hi}CD27^{+}B Cells After LD-IL-2 Therapy

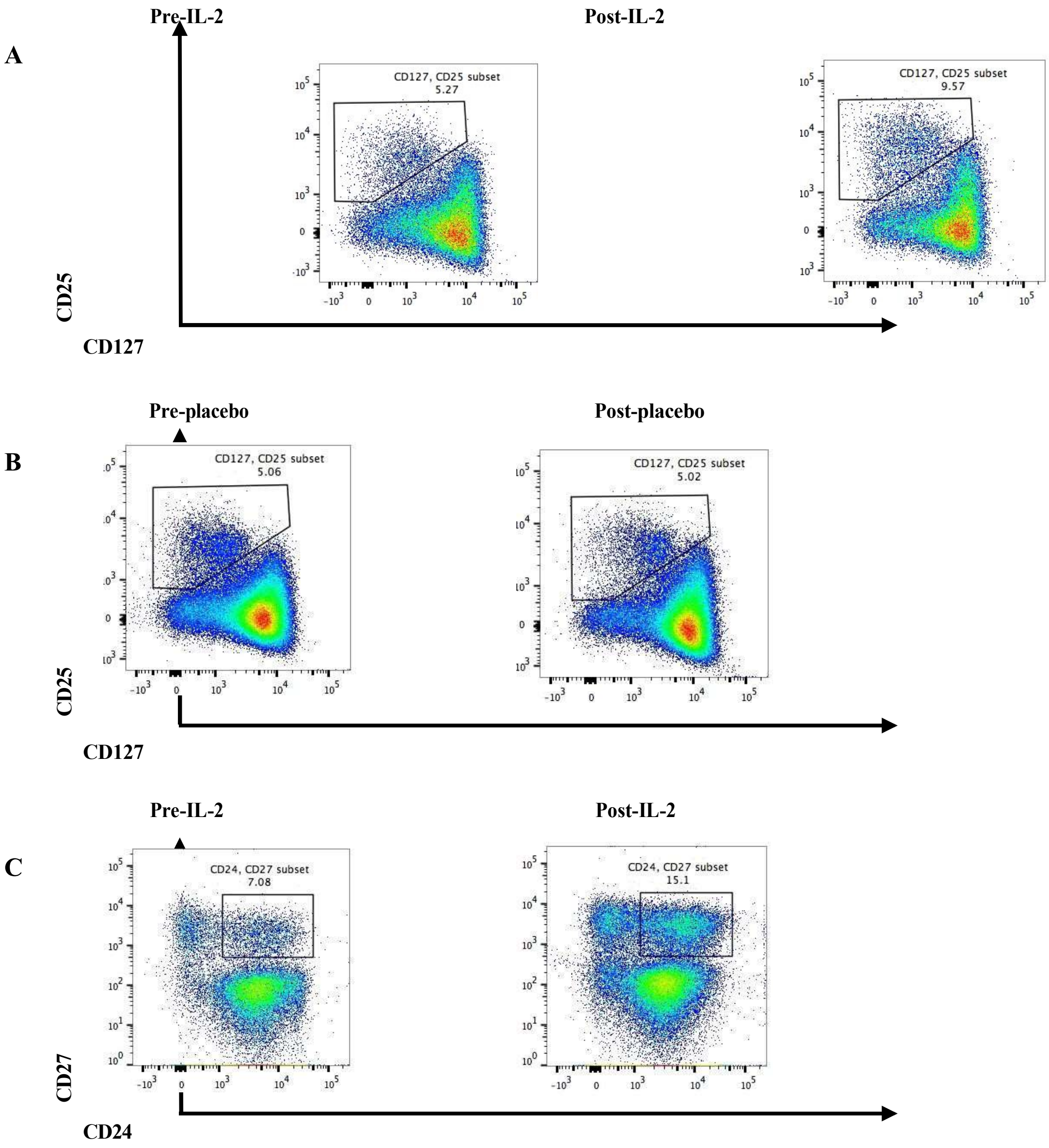

© 2022 He J et al. *JAMA Network Open.*

D

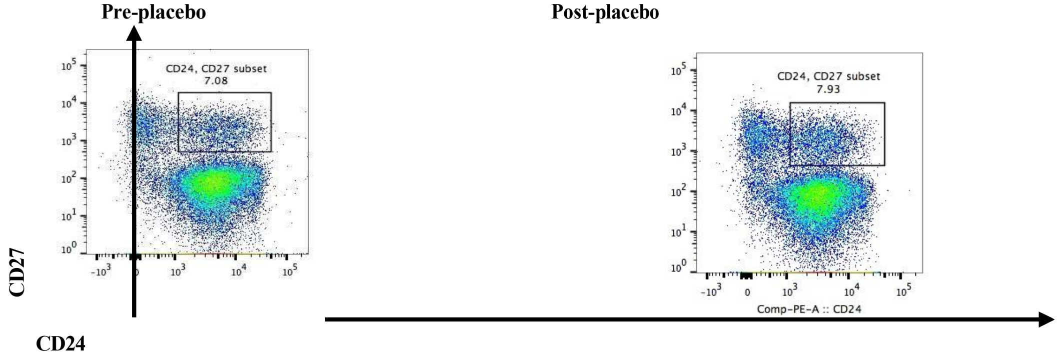

(A and B) Flow cytometry results showing the changes of Treg ($CD4^{+}CD25^{hi}CD127^{low}$) before and after Ld-IL2 and placebo treatment. (**C and D**) Flow cytometry results showing the changes of $CD19^{+}CD24^{hi}CD27^{+}$ B cells before and after Ld-IL2 and placebo treatment.

© 2022 He J et al. *JAMA Network Open.*

eFigure 2. Flow Cytometry Results Showing CD25 Expression on Different B Cell Subsets and the Changes of CD24^{hi}CD27^{+} B Cells and Cytokines After Co-Stimulated With IL-2 In Vitro

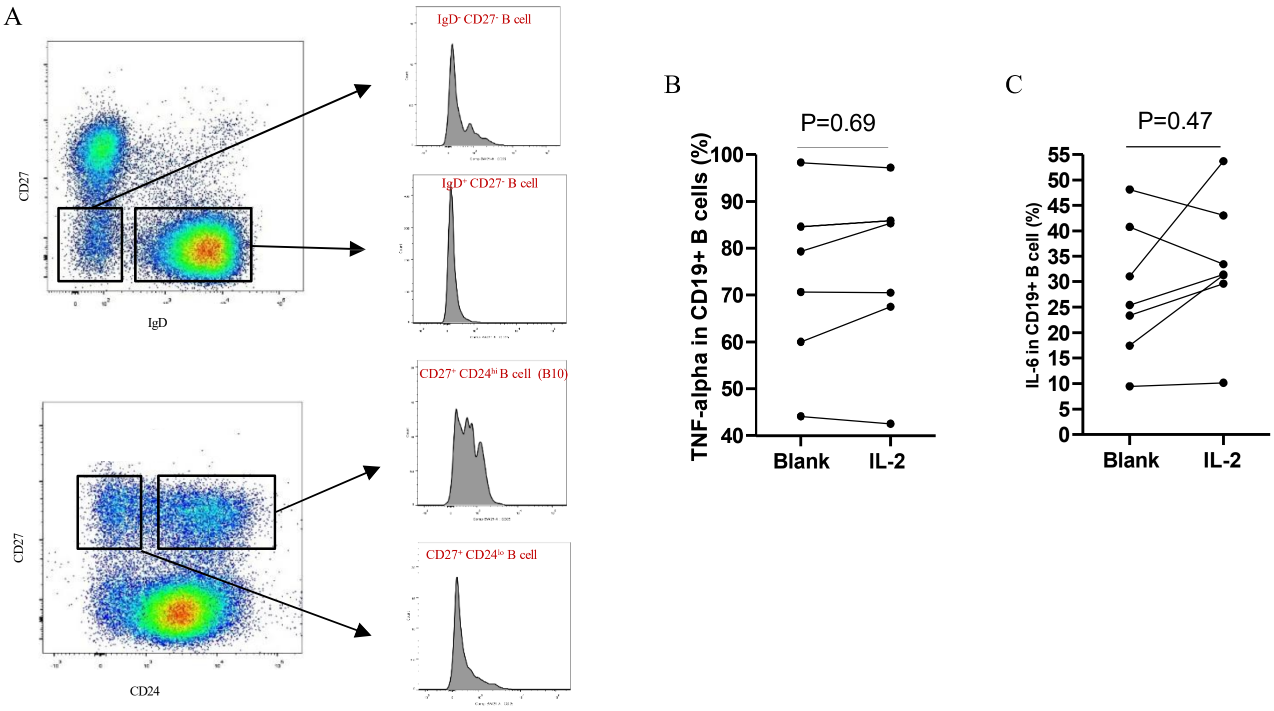

(A) CD24^{hi}CD27^{+} B cells are defined as B10 cells. **(B) to (C)** The changes of TNF-α and IL-6 after IL-2 co-stimulated treatment.

© 2022 He J et al. *JAMA Network Open.*

eFigure 3. **Immunological Responses to LD-IL-2 Therapy**

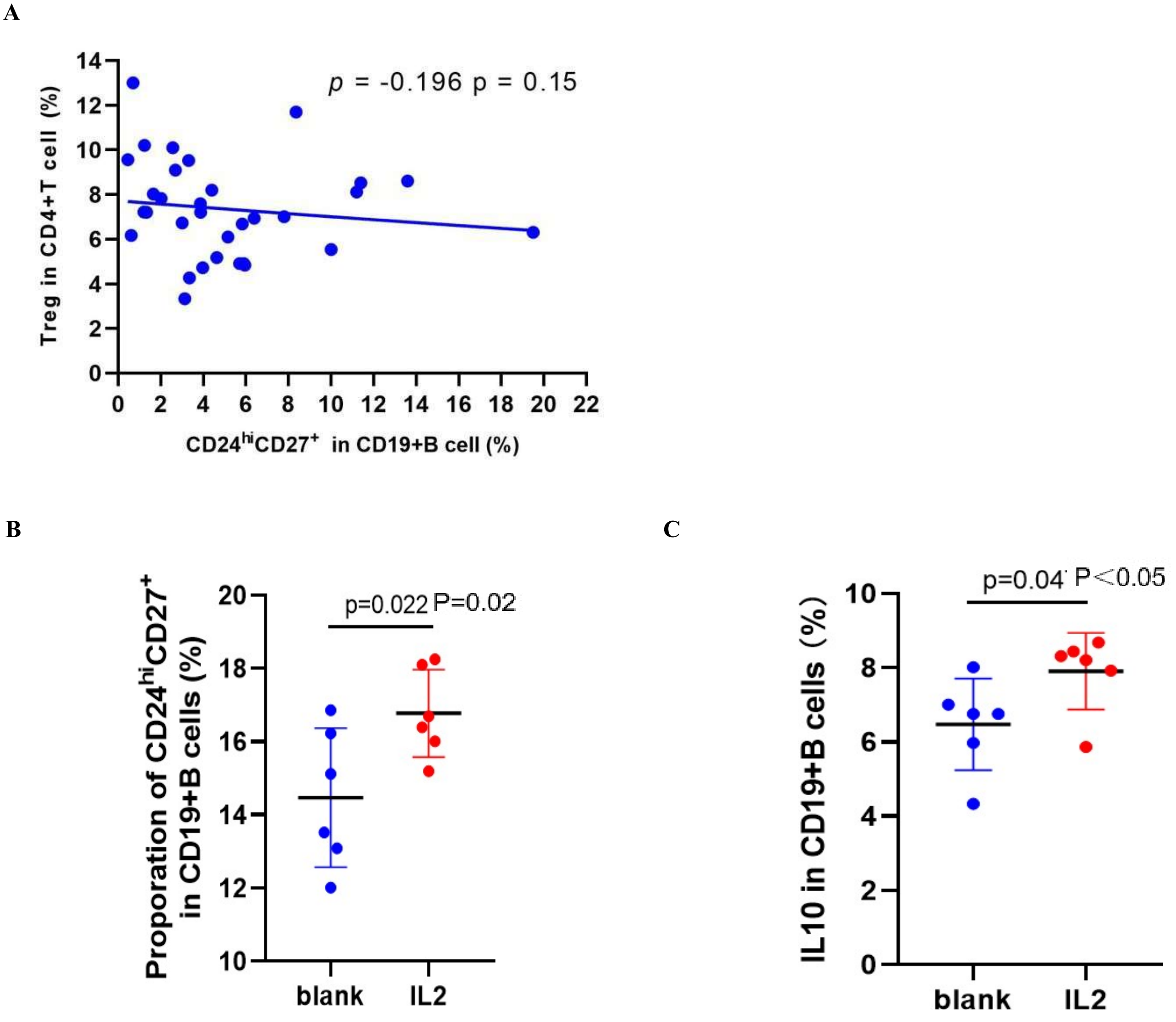

(A) There was a negative relation between CD24^{hi}CD27^{+} in CD19^{+} B cells and Treg in CD4^{+} T cells without significant differences. **(B)** IL-2 co-stimulated treatment induced increased CD24^{hi}CD27^{+} in CD19^{+} B cells. **(C)** IL-2 co-stimulated treatment induced increased IL-10 expression in CD19^{+}B cells. *,<0.05.

© 2022 He J et al. *JAMA Network Open.*

eFigure 4. Serum BAFF Expression on SS and HC

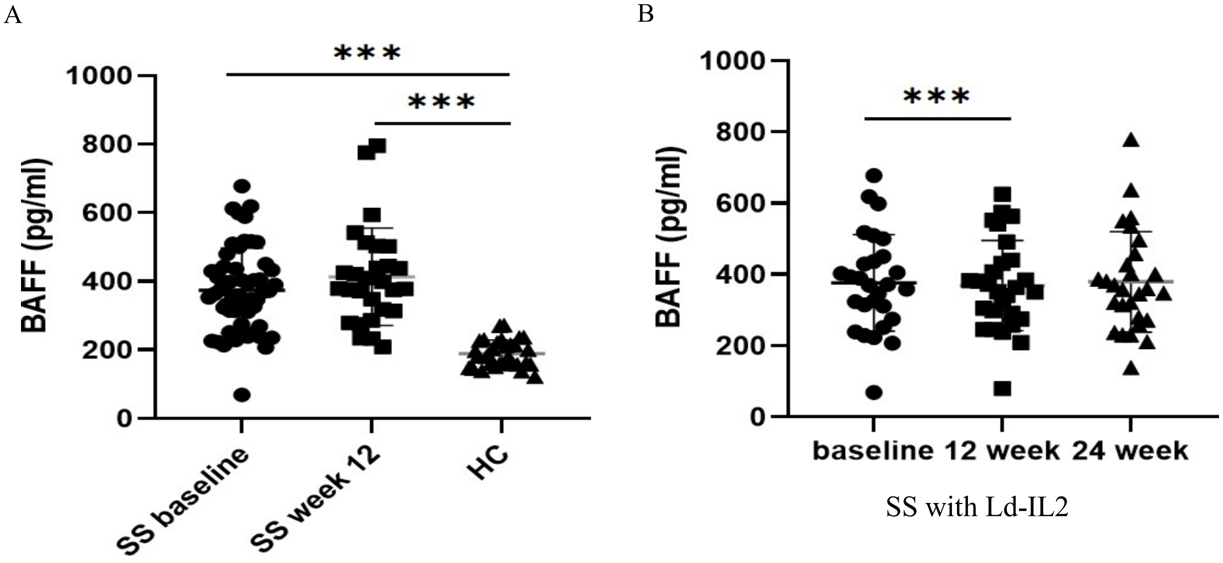

A. Serum BAFF levels in SS patient (baseline and week 12 after treatment) and HC. *P* values are based on *Student's t-test*.
B. Change of serum BAFF concentration at baseline, week 12 and week 24 after IL-2 treatment.. *P* values are based on *matched samples t-test.*

At week 0, the BAFF level in SS patients was 363.80 (208.00-588.78) pg/ml, significantly higher than HC 181.50 (151.42-217.36) pg/ml. *,<0.05; **,<0.01; ***,<0.001.

© 2022 He J et al. *JAMA Network Open.*

eFigure 5. Changes of Serum IFN-α in Patients With Fatigue

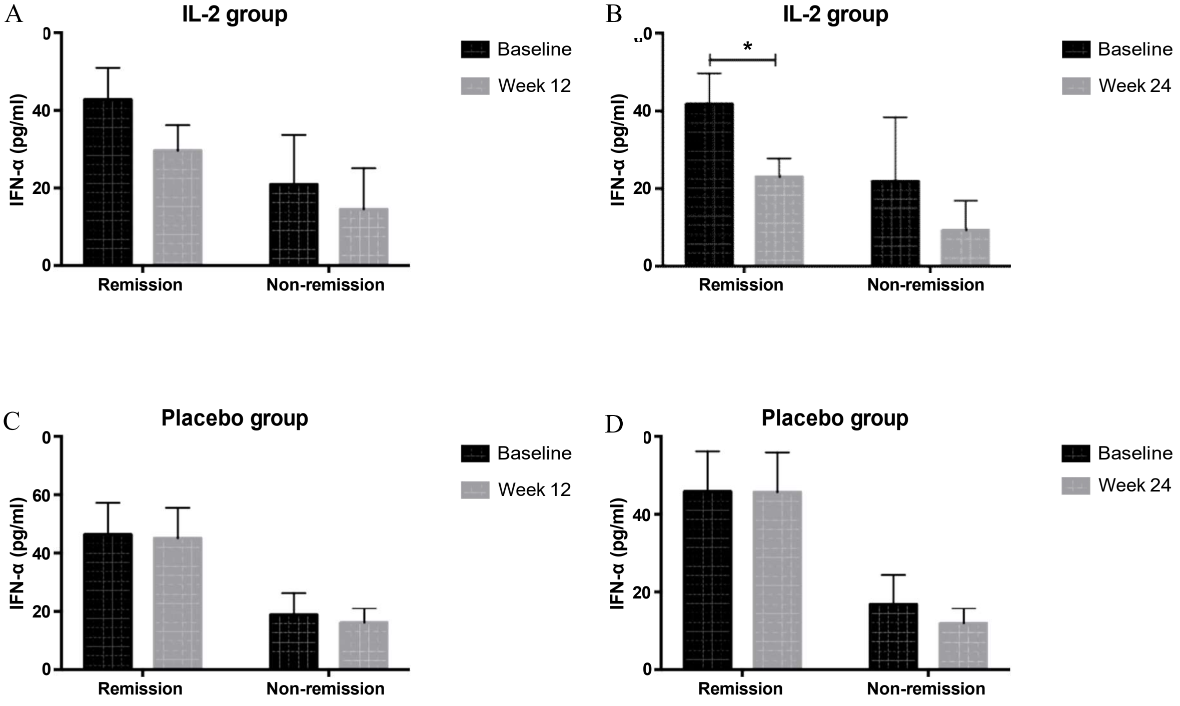

(A) Change in serum IFN concentrations between patients with fatigue remission and those without remission in the IL-2 arm at week 12. (B) Change in serum IFN concentrations between patients with fatigue remission and those without remission in the IL-2 arm at week 24. (C) Change in serum IFN concentrations between patients with fatigue remission and those without remission in the placebo arm at week 12. (D) Change in serum IFN concentrations between patients with fatigue remission and those without remission in the placebo arm at week 24.

© 2022 He J et al. *JAMA Network Open.*

eTable 1. Inclusion and Exclusion Criteria

Inclusion criteria

1. Men or women 18-70 years of age (or the legal age of consent in the jurisdiction in which the study is taking place).
2. Diagnosis of primary Sjögren's syndrome (according to the 2002 AECG classification criteria) for ≥3 months before screening.
3. European League Against Rheumatism Sjögren's Syndrome Disease Activity Index (ESSDAI) ≥5.
4. Scores of at least 50mm on at least 2 of 3 VASs (scores range from 0 [none] to 100mm [worst] for dryness, pain, and fatigue).
5. Positive tests for RF, anti-SSA or anti-SSB antibodies.
6. Active disease (IgG ≥16.8 g/L [upper limit of normal level]) at the time of enrolment.
7. Three months of stable background treatment with corticosteroids (≤7.5 mg/d prednisone or equivalent) and/or with antimalarials, nonsteroidal anti-inflammatory drugs or immunosuppressants.

Exclusion criteria

1. Secondary Sjögren's syndrome.
2. Stable disease activity (IgG ＜16.8 g/L [upper limit of normal level]) at the time of enrolment.
3. Prior rituximab or monoclonal antibody usage.
4. Severe comorbidities: including
   a. Heart failure (≥ grade III NYHA);
   b. Renal insufficiency (creatinine clearance ≤30 ml/min/rate);
   c. Hepatic insufficiency (serum ALT or AST >3 times the ULN, or total bilirubin >ULN for the central laboratory conducting the test).
5. Known allergies, hypersensitivity, or intolerance to IL-2 or its excipients.
6. History of severe allergic reaction to monoclonal antibodies or to murine, chimeric, or human proteins or their excipients.
7. Had a severe infection (including, but not limited to hepatitis, pneumonia, sepsis, or pyelonephritis); had been hospitalized for an infection; or had been treated with I.V. antibiotics for an infection, within 2 months prior to the first administration of study agent.
8. Chest radiograph within 3 months prior to the first administration of study agent that showed an abnormality suggestive of a malignancy or current active infection, including TB.
9. Had a nontuberculous mycobacterial infection or opportunistic infection (e.g., cytomegalovirus, pneumocystosis, aspergillosis) within 6 months prior to screening.
10. Infected with HIV (positive serology for HIV antibody) or hepatitis C (positive serology for Hep C antibody). If seropositive, consultation with a physician with expertise in the treatment of HIV or hepatitis C virus infection was recommended.
11. Infected with hepatitis B virus. For patients who were not eligible for this study due to hepatitis B virus test results, consultation with a physician with expertise in the treatment of hepatitis B virus infection was recommended.
12. Had any known malignancy or has a history of malignancy within the previous 5 years (with the exception of a nonmelanoma skin cancer that had been treated with no evidence of recurrence for ≥3 months before the first study agent administration or cervical neoplasia with surgical cure).
13. Had uncontrolled psychiatric or emotional disorder, including a history of drug and alcohol abuse within the past 3 years that might prevent the successful completion of the study.
14. Received, or was expected to receive, any live virus or bacterial vaccination within 3 months before the first administration of study agent, during the study, or within 4 months after the last administration of study agent. Had a BCG vaccination within 12 months of screening.
15. Was pregnant or breast-feeding.

AECG = American-European Consensus Group. RF = rheumatoid factor. IgG = immunoglobulin G. NYHA = New York Heart Association. ALT = alanine aminotransferase. AST = aspartate aminotransferase. ULN = upper limit of normal. TB = tuberculosis. HIV = human immunodeficiency virus. BCG = Bacille Calmette- Guérin.

© 2022 He J et al. *JAMA Network Open.*

eTable 2. **Adverse Events in Participants Who Received LD-IL-2 or Placebo Treatment**[a]

	Low-dose IL-2 (n=30)	Placebo (n=30)	P Value
Injection site reaction, No. (%)	3 (10.0)	0 (0.0)	0.12
Fever after injection, No. (%)	1 (3.3)	1 (3.3)	1.00
Hepatic enzyme increased [b]	1 (3.3)	0 (0.0)	1.00
Infection, No. (%)	1 (3.3)	9 (30.0)	0.006
Upper respiratory tract infection	1 (3.3)	5 (16.7)	0.20
Urinary tract infection	0 (0.0)	3 (10.0)	0.12
Herpes zoster	0 (0.0)	1 (3.3)	1.000

a Adverse events were collected at each visit via inquiry and clinical laboratory tests.
The safety population included all patients who were randomized and received at least 1 dose of study drug.
If a patient had multiple types of adverse events, he/she was counted once for each type.
b Aspartate aminotransferase or alanine aminotransferase at least 3 times the upper limit of normal.
IL-2=Interleukin-2.

© 2022 He J et al. *JAMA Network Open.*

eTable 3. **Monoclonal Antibodies Used in Flow Cytometric Analysis**

Antigen	Clone	Fluorochrome	Vendor
CD4	RPA-T4	Alexa Fluor 700	Biolegend
CD19	HIB19	Brilliant Violet 785	Biolegend
CD3	OKT3	Brilliant Violet 650	Biolegend
TCRαβ	IP26	FITC	Biolegend
CD8	RPA-T8	Brilliant Violet 510	Biolegend
CD25	M-A251	PE-CF594	BD Biosciences
CD127	A019D5	Brilliant Violet 605	Biolegend
CD45RA	HI100	APC-Cy7	Biolegend
CD24	eBioSN3 (SN3 A5-2H10)	PE	eBioscience
CD27	O323	APC	Biolegend
CD38	HIT2	Brilliant Violet 421	Biolegend
IgD	IA6-2	FITC	Biolegend

© 2022 He J et al. *JAMA Network Open.*

eTable 4. Decreased Scores From Baseline at Week 12 and Week 24 in Every Domain of ESSDAI

Domains of ESSDAI	Week 12			Week 24		
	Ld-IL2	Placebo	*P* value	Ld-IL2	Placebo	*P* value
Glandular	1.33 ± 1.30	0.46 ± 0.88	0.07	1.67 ± 1.44	0.77 ± 1.88	0.008
Articular	1.80 ± 0.63	0.25 ± 0.71	0.001	2.0	0.31 ± 0.75	0.01
Cutaneous	3.0	NA	NA	3.0	1.0 ± 1.07	NA
Pulmonary	3.18 ± 3.37	0.45 ± 1.51	0.01	3.64 ± 3.23	0.91 ± 2.02	0.01
Renal	NA	NA	NA	NA	NA	1.000
PNS	NA	NA	NA	5.0	NA	0.32
Hematological	0.88 ± 1.26	0.67 ± 1.95	0.63	-1.38 ± 1.20	0.27 ± 2.37	0.02
Biological	0.27 ± 0.74	0.27 ± 0.58	0.76	0.4 ± 0.77	0.3 ± 0.53	0.92
Constitutional	NA	NA	NA	NA	NA	NA
Lymphadenopathy and lymphoma	NA	NA	NA	NA	NA	NA
Muscular	NA	NA	NA	NA	NA	NA
Central nervous system	NA	NA	NA	NA	NA	NA

PNS, peripheral nervous system.
Data were presented as mean ± SD. NA, not available for lacking of enough variable.
R, Response; S, Supplementary.

© 2022 He J et al. *JAMA Network Open.*

eTable 5. **Responses of Laboratory Parameters in Participants**

	Baseline	Week 12	Week 24	*P* value (W0-W12)	*P* value (W0-W24)
WBC, ×10⁹/L					
IL-2, n=11	3.29 (2.71-3.49)	3.61 (3.06-6.99)	4.04 (2.37-8.77)	0.002	0.04
Placebo, n=10	3.24 (2.39-3.46)	3.74 (2.46-4.10)	4.06 (2.22-4.79)	0.72	0.95
Neutrophil, ×10⁹/L					
IL-2, n=11	1.80 (1.54-2.22)	2.05 (1.63-3.60)	2.28 (1.66-2.74)	0.58	0.78
Placebo, n=10	2.02 (1.88-2.12)	1.75 (1.13-2.46)	1.65 (1.32-2.55)	0.43	0.37
Lymphocyte, ×10⁹/L					
IL-2, n=11	1.01 (0.76-1.47)	1.02 (0.73-1.46)	1.09 (0.80-1.39)	0.90	0.96
Placebo, n=10	1.09 (0.77-1.58)	1.19 (1.05-1.55)	1.16 (1.00-1.69)	0.43	0.71
Platelet, ×10⁹/L					
IL-2, n=5	104 (20-123)	145 (106-220)	149 (116-184)	0.11	0.72
Placebo, n=6	101 (64-120)	113 (65-147)	117 (73-150)	0.95	0.96
γ-G, %					
IL-2, n=29	26.2 (16.9-36.8)	25.9 (16.3-35.7)	25.0 (17.9-34.5)	0.57	0.51
Placebo, n=28	25.6 (17.7-36.6)	26.2 (18.1-35.6)	25.7 (20.0-35.0)	0.80	0.37
IgA, g/L					
IL-2, n=29	3.94 (1.56-6.83)	3.80 (1.5-6.68)	3.96 (1.54-7.50)	0.81	0.05
Placebo, n=28	3.99 (1.83-6.58)	3.79 (1.74-6.38)	3.63 (2.16-7.09)	0.91	0.50
IgG, g/L					
IL-2 , n=29	23.1 (17.7-35.5)	22.4 (17.6-34.2)	22.5 (13.4-35.5)	0.15	0.04
Placebo, n=28	22.7 (16-34.9)	22.5 (16.2-35.1)	23.8 (14.4-36.2)	0.12	0.21
ESR, mm/hr					
IL-2 , n=29	31 (2-93)	27 (11-55)	25 (7-70)	0.12	0.21
Placebo, n=28	29 (5-89)	25 (5-86)	29 (7-85)	0.45	0.89
Anti-SSA, IU/mL					
IL-2, n=29	208.6 (105.8-269.2)	201.8 (94.9-224.6)	203.1 (62.9-224.7)	0.09	0.15
Placebo, n=28	210.3 (154.0-229.0)	210.3 (154.5-235.3)	210.9 (153.2-228.3)	0.98	0.43
Anti-SSB, IU/mL					
IL-2, n=29	65.1 (0.8-239.4)	44.1 (0.8-235.1)	40.4 (0.9-217.3)	0.34	0.47
Placebo, n=28	59.9 (2.1-283.9)	46.9 (2.3-279.3)	63.4 (1.5-249.4)	0.34	0.67
RF, IU/mL					
IL-2, n=29	190 (20-2520)	65.3 (20-971)	96.3 (20-1020)	0.19	0.47
Placebo, n=28	94.2 (20-1510)	92.7 (20-1660)	99.9 (20-1630)	0.25	0.10
DLCO, %					
IL-2, n=12	66.8 (61.2-70.1)	72.4 (70.3-79.8)	76.4 (71.9-80.2)	0.01	0.003
Placebo, n=11	69.6 (59.4- 73.6)	70.6 (62.2-86.1)	72.5 (66.5-85.5)	0.63	0.22
FVC, %					
IL-2, n=12	87.3 (77.7-95.8)	100.2 (71.2-123.4)	102.3 (75.3-120.3)	0.06	0.03
Placebo, n=11	90.0 (65.7-100.0)	91.0 (66.8-96.5)	89.2 (66.8-98.0)	0.67	0.51

Data are mean(SD), median (IQR) or n (%). BSA=Body surface area. ESSPRI=European League Against Rheumatism (EULAR) Sjögren's syndrome patient- reported index. ESSDAI=European League Against Rheumatism (EULAR) Sjögren's syndrome disease activity index. SF-36=Short Form (36 Items) Health Survey. MFI-20=Multidimensional Fatigue Inventory-20. WBC=white blood cell. γ-G=γ-globulin. IgA=immunoglobulin A. IgG=immunoglobulin G. ESR=erythrocyte sedimentation rate. RF=rheumatoid factor. FVC=Forced Vital Capacity. DLCO=Diffusing capacity of the Lung for Carbon Monoxide. β2-MG=beta-2-microglobulin. NAG=N-acetyl-β-glucosaminidase. RBP=Retinol-Binding Protein. NA=Not applicable.

© 2022 He J et al. *JAMA Network Open.*

eTable 6. **Percentage of Clinical Manifestations in Different Time Points of Both Groups**

Clinical manifestations	IL-2 group			Placebo group		
	**Baseline**	**Week 12**	**Week 24**	**Baseline**	**Week 12**	**Week 24**
Parotid gland enlargement	12 (100.0)	3 (25.0)	3 (25.0)	13 (100.0)	11 (84.6)	10 (76.9)
Articular	10 (100.0)	1 (10.0)	0 (0.0)	8 (100.0)	5 (62.5)	6 (75.0)
Leukopenia	12 (100.0)	10 (83.3)	8 (66.7)	7 (100.0)	6 (85.7)	8 (100.0) [a]
Anemia	3 (100.0)	2 (66.7)	2 (66.7)	7 (100.0)	4 (57.1)	5 (71.4)
Thrombocytopenia	4 (100.0)	2 (50.0)	2 (50.0)	7 (100.0)	4 (57.1)	3 (42.9)
Pulmonary	12 (100.0)	4 (33.3)	3 (25.0)	11 (100.0)	6 (54.5)	5 (45.5)
Renal	2 (100.0)	2 (100.0)	2 (100.0)	3 (100.0)	3 (100.0)	3 (100.0)
Neurologic	1 (100.0)	1 (100.0)	0 (0.0)	1 (100.0)	1 (100.0)	1 (100.0)
Cutaneous	1 (100.0)	0 (0.0)	0 (0.0)	0 (0.0)	0 (0.0)	0 (0.0)

[a] 8 patients suffered from leukopenia.

© 2022 He J et al. *JAMA Network Open.*

eTable 7. **Change in Ocular Parameters after LD-IL-2 Therapy**

Characteristics	Baseline	Week 12	Week 24	*P* value (W0-W12)	*P* value (W0-W24)	*P* value (W12-W24)
BUT-OD, s						
IL-2	4.29 (3.18)	4.94 (4.09)	4.54 (4.23)	0.41	0.66	0.64
Placebo	4.50 (4.72)	5.06 (4.99)	5.57 (8.74)	0.45	0.94	0.43
BUT-OS, s						
IL-2	4.36 (4.06)	5.07 (4.44)	4.32 (3.58)	0.47	0.62	0.43
Placebo	4.09 (3.38)	5.37 (4.97)	4.76 (7.41)	0.18	0.64	0.61
TMH-OD, mm						
IL-2	15.10 (10.07)	13.63 (12.36)	13.44 (10.01)	0.48	0.25	0.52
Placebo	14.96 (9.74)	14.44 (9.29)	14.04 (9.05)	0.34	0.19	0.90
TMH-OS, mm						
IL-2	15.29 (10.88)	13.96 (16.29)	12.84 (12.87)	0.31	0.25	0.28
Placebo	16.00 (11.27)	16.54 (16.29)	14.24 (10.35)	0.35	0.34	0.49
Number of MG-OD						
IL-2	12(3-21)	12(0-22)	13(0-24)	0.41	0.28	0.74
Placebo	13(6-20)	14(4-24)	15(4-26)	0.67	0.27	0.54
Number of MG-OS						
IL-2	12(0-25)	13(0-22)	13(0-25)	0.79	0.37	0.45
Placebo	13(3-22)	12(4-23)	15(2-28)	0.99	0.52	0.49
Schirmer I test-OD, mm						
IL-2	2.44 (4.51)	2.59 (4.32)	2.01 (4.64)	0.45	0.47	0.69
Placebo	2.17 (5.58)	2.45 (4.96)	2.50 (5.03)	0.68	0.59	0.61
Schirmer I test-OS, mm						
IL-2	3.65 (6.44)	3.32 (5.53)	2.66 (4.05)	0.39	0.39	0.36
Placebo	2.89 (5.98)	2.46 (5.28)	2.25 (4.52)	0.44	0.46	0.31
MG dropout ratio in upper lids-OD, %						
IL-2	43.7 (45.7)	49.9 (39.7)	47.8 (40.5)	0.43	0.41	0.87
Placebo	46.1 (35.4)	45.0 (32.6)	42.6 (36.5)	0.81	0.51	0.62
MG dropout ratio in upper lids-OS, %						
IL-2	46.2 (35.4)	44.9 (32.6)	42.6 (36.5)	0.34	0.32	0.27
Placebo	46.1 (30.3)	42.5 (29.2)	41.8 (36.4)	0.17	0.71	0.18

Data are mean ± SD or median (interquartile range). OD = oculus dexter. OS = oculus sinister. BUT = break-up time. TMH = tear meniscus height. MG = Meibomian gland. This table excludes 3 patients who were enrolled but did not complete any follow-up visits.

Ocular measures：All patients underwent a complete examination of the ocular surface of both eyes followed by a masked operator: tear film break-up time (TBUT), noninvasive tear film break-up time (NIKBUT), noninvasive tear meniscus height (NIKTMH), Schirmer I test, meibography and IVCM analysis of the central cornea subbasal nerves. NIKTMH, NIKBUT and meibography were performed with Oculus Keratograph 5M (Oculus Keratograph, Oculus, Wetzlar, Germany). Partial or complete loss of meibomian glands was scored using the grades described by Reiko[1]: 0, no loss of meibomian glands; 1, the loss of area was 1/3 of the total meibomian gland area; 2, the loss of area was between 1/3 and 2/3; and 3, the loss of area was more than 2/3. Scores for the upper and lower eyelids were analyzed using ImageJ (ImageJ; National Institutes of Health, Bethesda, MD) and summed to obtain a score for each eye (0–6). TBUT was measured by instilling fluorescein into the inferior culdesac and calculating the average of two consecutive break-up times. Corneal staining was evaluated using the National Eye Institute (NEI) scale after the instillation of fluorescein. Schirmer I test was conducted by inserting Schirmer strips (Jingming Co., Ltd., Tianjing, China) into the lower conjunctival sac at the junction of the lateral and middle thirds without anesthesia for 5 min and wetting of the strips was recorded in millimeters.

Reference

1. Arita R, Fukuoka S, Morishige N. Therapeutic efficacy of intense pulsed light in patients with refractory meibomian gland dysfunction. Ocul Surf. 2019 Jan;17(1):104-110. doi: 10.1016/j.jtos.2018.11.004. Epub 2018 Nov 13. PMID: 30445177

© 2022 He J et al. *JAMA Network Open.*

eTable 8. Change in Salivary Gland Ultrasonography Scan Score After LD-IL-2 Therapy

Characteristics	Baseline	Week 12	Week 24	*P* value (W0 -W12)	*P* value (W0 - W24)	*P* value (W12 -W24)
SGUS Score						
LD-IL2	10 (8-12)	9.5 (8-10)	9 (8-11)	0.62	0.52	0.82
Placebo	12 (8-14)	10 (8-12)	11 (8-12)	0.42	0.56	0.64

Method:
The US system was equipped with a high-resolution linear transducer (9-12 MHz). The bilateral parotid and submandibular glands were scanned. The novel four-grade semiquantitative scoring system (grade 0–3) developed for the OMERACT was employed to assess the echostructure of each gland. Fatty replacement and fibrosis were considered during SGUS scoring when the semiquantitative scoring system could not be applied; hyperechoic (fibrous) bands (grade 3) and fatty replacement (grade 1) were considered part of the overall semiquantitative scoring system. In each participant, the grades for the parotid and submandibular glands on one side (right or left) and the grades of all four glands were considered in the analysis.

Reference:
Jousse-Joulin S, D'Agostino MA, Nicolas C, et al. Video clip assessment of a salivary gland ultrasound scoring system in Sjogren's syndrome using consensual definitions: an OMERACT ultrasound working group reliability exercise. *Ann Rheum Dis* 2019;78:967-73.

© 2022 He J et al. *JAMA Network Open.*

eTable 9. **Immunological Responses of T Cell Subtypes to LD-IL-2 Therapy**

variables	Baseline	Week 2	Week 4	Week 6	Week 8	Week 10	Week 12	Week 16	Week 20	Week 24
CD4$^+$ T cells (%)										
IL-2 median(quartile)	23.50(19.00,31.10)	26.75(19.45,34.15)	28.90(21.60,31.55)	29.60(20.30,30.75)	23.35(20.35,32.95)	28.40(24.40,35.40)	27.20(19.85,31.40)	23.00(21.20,28.20)	25.45(19.23,30.45)	21.60(17.05,27.65)
Placebo median(quartile)	21.80(15.20,34.00)	29.70(18.80,34.70)	29.80(21.45,39.08)	23.75(20.75,34.83)	25.90(19.63,37.30)	23.10(13.80,37.20)	25.55(17.73,33.95)	22.80(14.90,36.35)	27.20(18.60,35.70)	25.20(19.00,34.05)
CD8$^+$T cell(%)										
IL-2 median(quartile)	16.20(12.30,22.50)	17.55(11.25,24.08)	14.00(10.75,26.80)	16.90(11.85,26.45)	20.15(10.55,24.98)	17.20(13.40,24.20)	16.90(10.95,22.15)	19.80(15.00,27.20)	22.75(14.58,26.28)	17.40(12.00,28.15)
Placebo median(quartile)	17.40(10.56,24.55)	19.80(13.60,24.90)	19.00(14.45,24.30)	20.50(17.33,25.30)	21.80(15.60,25.05)	20.30(12.40,25.90)	21.10(16.45,23.50)	22.95(12.63,25.75)	23.25(19.08,28.40)	21.10(17.30,27.93)
Tfh cell(%)										
IL-2 median(quartile)	10.70(9.27,15.05)	10.50(8.01,18.95)	11.95(9.10,18.05)	13.30(10.00,19.70)	11.85(9.13,18.15)	12.90(10.58,16.33)	11.30(9.24,12.15)	14.10(10.00,15.90)	13.20(9.11,17.55)	13.80(11.20,15.75)
Placebo median(quartile)	9.93(7.75,16.70)	11.50(6.90,15.90)	11.65(7.76,20.13)	14.40(9.90,17.30)	11.70(8.88,20.10)	13.65(8.82,17.48)	14.10(10.02,16.45)	16.15(10.62,19.95)	13.30(11.88,17.73)	14.55(10.53,19.08)
Treg cell(%)										
IL-2 median(quartile)	7.22(5.18,9.20)	12.15(7.05,14.05)	8.62(5.88,10.45)	11.30(7.85,14.15)	8.22(6.00,10.73)	9.65(7.40,12.70)	7.65(6.96,10.65)	7.41(5.71,8.77)	8.21(6.04,10.18)	8.63(6.46,9.36)
Placebo median(quartile)	6.98(6.15,8.96)	6.84(4.95,8.81)**	7.16(5.49,9.12)	7.01(5.39,9.61)**	7.88(5.95,9.66)	7.88(5.65,9.69)*	6.86(5.22,9.08)	6.80(6.02,9.89)	7.34(5.00,10.10)	7.74(5.31,9.34)
Th1 cell(%)										
IL-2 median(quartile)	28.20(24.60,36.20)	31.60(20.51,34.68)	31.00(16.55,38.05)	24.90(18.70,31.80)	24.20(12.95,32.83)	27.40(22.53,36.08)	21.35(15.83,28.30)	21.90(10.50,30.60)	22.80(17.65,34.15)	24.80(15.88,34.00)
Placebo median(quartile)	27.90(17.45,33.50)	27.60(22.50,31.45)	23.90(18.58,35.43)	23.00(14.00,27.60)	25.00(20.25,36.48)	20.65(13.93,27.15)	23.20(13.60,32.60)	19.10(14.60,30.35)	25.50(21.00,29.10)	28.10(15.16,34.95)
Th2 cell(%)										
IL-2 median(quartile)	41.70(30.15,60.78)	35.10(32.68,58.13)	46.50(39.30,62.40)	53.45(39.50,59.40)	41.35(36.03,55.45)	58.15(41.60,71.00)	54.90(45.80,68.60)	43.50(39.75,56.40)	49.60(42.15,59.93)	41.70(30.15,60.78)
Placebo median(quartile)	47.90(30.55,64.90)	45.40(34.80,60.20)	49.10(40.40,69.15)	47.50(30.30,59.73)	56.85(44.13,76.63)	53.20(40.00,63.75)	58.60(37.55,70.70)	52.75(36.63,67.05)	38.60(31.90,68.15)	47.90(30.55,64.90)
Th17 cell(%)										
IL-2 median(quartile)	17.10(12.55,20.85)	15.10(7.43,20.98)	16.80(15.00,19.70)	15.40(13.10,18.60)	14.35(9.42,16.83)	16.55(13.41,18.08)	15.15(7.18,17.05)	16.00(11.30,22.40)	17.80(13.45,22.35)	17.15(12.35,21.30)
Placebo median(quartile)	13.90(8.11,19.80)	17.00(13.20,21.13)	17.00(13.00,20.68)	15.40(11.05,19.85)	16.85(14.70,19.25)	16.40(13.80,20.10)	16.50(11.01,21.85)	17.55(10.58,24.95)	15.90(14.20,22.05)	17.05(12.45,20.88)
Treg/Th17										
IL-2 median(quartile)	0.42(0.34,0.66)	0.74(0.49,1.49)	0.51(0.38,0.72)	0.65(0.46,0.86)	0.60(0.33,0.85)	0.65(0.38,0.81)	0.63(0.43,1.22)	0.49(0.25,0.68)	0.47(0.26,0.91)	0.44(0.31,0.87)
Placebo median(quartile)	0.67(0.41,1.17)	0.37(0.31,0.67)*	0.49(0.28,0.69)	0.49(0.36,0.69)	0.46(0.33,0.63)	0.54(0.32,0.68)	0.61(0.33,0.92)	0.38(0.31,1.21)	0.58(0.35,0.82)	0.41(0.25,0.93)
Treg/Tfh										
IL-2 median(quartile)	0.60(0.46,0.85)	0.94(0.65,2.04)	0.64(0.44,1.03)	0.92(0.56,1.31)	0.68(0.49,0.93)	0.71(0.51,0.96)	0.63(0.58,1.00)	0.60(0.37,1.01)	0.58(0.45,0.78)	0.50(0.41,0.62)
Placebo median(quartile)	0.72(0.35,1.17)	0.59(0.38,1.04)*	0.65(0.30,0.93)	0.54(0.45,0.64)**	0.61(0.34,0.88)	0.61(0.37,0.83)	0.52(0.37,0.83)*	0.48(0.27,0.69)	0.51(0.37,0.65)	0.51(0.32,0.67)

© 2022 He J et al. *JAMA Network Open.*

eTable 10. Change in Cytokines After LD-IL-2 Therapy

Characteristics	Baseline	Week 12	Week 24	*P* value (W0-W12)	*P* value (W0-W24)
IL-2, pg/ml					
IL-2	5.68 (0.11-40.91)	11.72 (0.38-55.40)	8.68 (0.52-31.46)	0.06	0.009
Placebo	5.32 (0.59-37.31)	3.62 (0.55-36.74)	3.04 (0.45-37.38)	0.57	0.44
TGF-β, ng/ml					
IL-2	1.55 (0.43-3.58)	1.35 (0.45-3.99)	1.33 (0.63-4.13)	0.33	0.97
Placebo	1.26(0.46-3.88)	1.52 (0.52-3.51)	1.52(0.51-3.52)	0.53	0.77
IFN-α, pg/ml					
IL-2	19.62 (1.72-131.30)	12.19 (0.10-90.92)	9.13 (0.17-74.22)	<0.001	0.03
Placebo	13.68 (1.74-138.53)	11.35 (1.44-129.68)	13.84 (1.69-130.07)	0.24	0.37
IL-6, pg/ml					
IL-2	6.09 (0.93-19.36)	8.37 (1.65-24.96)	5.92 (1.53-26.64)	0.11	0.50
Placebo	3.69 (1.14-25.88)	4.30 (2.06-19.22)	5.07 (1.62-19.94)	0.22	0.52
IL-21, pg/ml					
IL-2	18.86 (0.39-221.22)	30.91 (0.29-231.52)	15.62 (0.49-192.91)	0.23	0.25
Placebo	6.30 (1.82-265.51)	7.42 (0.59-240.06)	7.27 (0.49-245.15)	0.32	0.26
IL-7, pg/ml					
IL-2	22.44 (2.14-238.66)	31.24(1.61-235.18)	15.80 (0.33-215.41)	0.51	0.38
Placebo	9.09 (1.27-267.39)	10.84(1.78-378.63)	8.53 (0.48-323.38)	0.76	0.30
IFN-γ, pg/ml					
IL-2	0.94 (0.32-4.10)	1.17 (0.22-4.22)	1.22 (0.25-4.78)	0.84	0.33
Placebo	0.94 (0.38-4.37)	0.80 (0.22-3.88)	0.81 (0.14-5.22)	0.78	0.44
IL-12p70, pg/ml					
IL-2	1.97 (0.84-9.93)	2.02 (0.82-8.80)	1.91 (0.58-8.94)	0.07	0.54
Placebo	2.32 (0.80-9.92)	1.96 (0.79-8.95)	1.95 (0.97-8.82)	0.81	0.29
IL-15, pg/ml					
IL-2	40.69 (0.38-254.59)	24.03 (0.25-254.82)	26.56 (0.13-225.04)	0.24	0.13
Placebo	42.16 (0.43-280.95)	45.71 (0.30-254.20)	32.16 (0.25-231.08)	0.19	0.23
IL-4, pg/ml					
IL-2	1.32 (0-23.67)	0.92 (0-15.17)	0.92 (0-19.51)	0.14	0.14
Placebo	1.08 (0-20.21)	1.08 (0-21.46)	1.00 (0-18.07)	0.66	0.15
IL-17A, pg/ml					
IL-2	1.64 (0.25-9.73)	1.22 (0-8.42)	1.25 (0-6.47)	0.03	0.39
Placebo	1.04 (0.065-8.86)	1.18 (0.21-7.77)	1.04 (0-6.80)	0.70	0.34
CXCL13, pg/ml					
IL-2	43.66 (5.83-111.18)	39.00 (13.37-106.56)	40.36 (9.48-99.47)	0.65	0.37
Placebo	36.36 (7.47-120.59)	34.67 (9.90-96.91)	35.51 (9.06-228.30)	0.12	0.29
IL-10, pg/ml					
IL-2	3.07 (1.17-13.38)	3.50 (1.09-16.45)	3.13 (1.34-14.88)	0.21	0.13
Placebo	3.01(1.18-12.12)	3.37 (1.29-13.77)	2.96 (1.32-12.09)	0.15	0.18
BAFF, pg/ml					
IL-2	363.80 (208.00-588.78)	378.44 (208.72-595.38)	362.77 (164.04-488.47)	<0.001	0.55
Placebo	369.29 (207.48-677.79)	351.58 (208.80-625.65)	358.07 (138.71-637.85)	0.61	0.84

Data are median (interquartile range). This table excludes 3 patients who were enrolled but did not complete any follow-up visits.

© 2022 He J et al. *JAMA Network Open.*

eTable 11. Changes of B Cell Subsets After LD-IL-2 Therapy

Subsets	Baseline	Week 12	Week 24	P value (Week 0 *vs* 12)	P value (Week 0 *vs* 24)
$CD19^+$ B cells (%)					
IL-2 group (quartile)	0.48 ± 0.39	0.45 ± 0.31	0.35 ± 0.22	0.20	0.22
Placebo group (quartile)	0.64 ± 0.55	0.45 ± 0.23	0.44 ± 0.33	0.78	0.16
$CD24^{hi}CD27^+$ in $CD19^+$ B cells (%)					
IL-2, median (quartile)	4.31 (1.28, 10.10)	6.03 (2.80, 12.25)	4.85 (2.65, 11.20)	0.01	0.05
Placebo, median (quartile)	3.54 (2.16, 8.29)	5.27 (2.55, 7.61)	4.24 (2.95, 9.31)	0.81	0.18
Naïve B in $CD19^+$ cells (%)					
IL-2, median (quartile)	87.70 (76.45, 92.95)	85.50 (67.95, 90.20)	81.10 (75.25, 89.95)	0.08	0.06
Placebo, median (quartile)	87.00 (83.95, 92.73)	86.45 (78.28, 90.25)	86.90 (76.75, 89.85)	0.12	0.04
Memory B cells (%)					
IL-2, median (quartile)	11.44 (6.63, 22.35)	13.01 (8.93, 26.86)	14.20 (8.31, 19.89)	0.29	0.39
Placebo, median (quartile)	11.15 (6.17, 13.12)	11.53 (8.60, 19.21)	10.16 (8.12, 20.20)	0.08	0.12

© 2022 He J et al. *JAMA Network Open.*
