# Supplementary material for: Efficacy and Safety of Low-Dose Interleukin 2 for Primary Sjögren Syndrome: A Randomized Clinical Trial
Source: JAMA Netw Open. 2022 Nov 10;5(11):e2241451. doi: 10.1001/jamanetworkopen.2022.41451 (PMC9650609; doi:10.1001/jamanetworkopen.2022.41451)
Supplement: Supplement 3. — Data Sharing Statement [file jamanetwopen-e2241451-s003.pdf]

Data Sharing Statement

He. Efficacy and Safety of Low-Dose Interleukin 2 for Primary Sjögren Syndrome. *JAMA Netw Open*. Published November 10, 2022. doi:10.1001/jamanetworkopen.2022.41451

Data
Data available: Yes
Data types: Deidentified participant data
How to access data: Data sharing is possible from the principal investigator Jing He. Peking University People's Hospital, China, hejing@pkuph.edu.cn
When available: With publication

Supporting Documents
Document types: None

Additional Information
Who can access the data: For clinical researchers for study purposes after a reasonable request.
Types of analyses: For predefined specified purposes such as individual meta-analysis of the topic.
Mechanisms of data availability: after a signed data access agreement
Any additional restrictions: All data elements that might reveal the identity of the participants directly or indirectly will be removed.
